# Tenting screw (tent pole) technique in vertical and horizontal alveolar ridge augmentations: a systematic review and meta-analysis

**DOI:** 10.1186/s40902-026-00522-y

**Published:** 2026-08-14

**Authors:** Bita Sarfaraz, Reza Amid, Ali Azadi, Mahdi Kadkhodazadeh, Amir-Ali Yousefi-Koma

**Affiliations:** 1 Dentofacial Deformities Research Center, Research Institute of Dental Sciences, Shahid Beheshti University of Medical Sciences, Tehran, Iran, Islamic Republic of; 2 Department of Periodontics, School of Dentistry, Shahid Beheshti University of Medical Sciences, Tehran, Iran, Islamic Republic of; 3 Dental Research Center, Research Institute of Dental Sciences, Shahid Beheshti University of Medical Sciences, Tehran, Iran, Islamic Republic of

**Keywords:** Tenting screw, Tent-pole technique, Tent-pole screw: dental implants, Alveolar ridge augmentation

## Abstract

**Purpose:**

Providing an overview on tenting screws/tent-pole technique for space-maintenance in vertical and horizontal alveolar ridge augmentation.

**Methods:**

The protocol was registered at PROSPERO (CRD420251178321). PRISMA-2020 guidelines were applied. Medline via PubMed, Web of Science, Scopus, Embase, and Cochrane Library were all searched/screened. Randomized and non-randomized human studies with at least 10 patients were eligible. Random-effects model analysis was used in all cases. The risk of publication bias was assessed using a funnel plot and the Egger’s test. All statistical analyses were executed using STATA 17.0.

**Results:**

Four randomized, 7 non-randomized and 13 cohort studies were included; 23 were selected for meta-analysis. Overall vertical and horizontal bone gains using tenting screws/tent-pole as the sole space-maintenance technique were at 5.43 mm (95% CI: 3.79–7.07) and 3.62 mm (95% CI: 3.21–4.02), respectively. One-staged augmentations/dental implantation showed significantly higher vertical gain compared to two-staged procedures (*P* = 0.03). Regarding complementary grafted bone materials/particles, autografts (with/without xenografts) had significantly higher vertical gains than allografts (with/without xenografts) (*P* = 0.00). Five cohorts compared the outcomes of tenting technique against conventional GBR (with no bone-blocks) in horizontal reconstruction; tenting technique significantly outperformed with 0.49 mm (95% CI: 0.00–0.97) superiority (*P* = 0.05); GRADE certainty assessment reported as moderate. There were no signs of publication bias in any of the analyses (*P* = 0.75, *P* = 0.75, and *P* = 0.23, respectively).

**Conclusions:**

Tent-pole technique is desirable for space-maintenance without the complexity and surgical/healing complications of onlay-bone-blocks and titanium meshes. More RCTs are required to reach reliable conclusions.

**Supplementary Information:**

The online version contains supplementary material available at 10.1186/s40902-026-00522-y.

## Introduction

Prevalence of edentulism is anticipated to increase with the aging population; dental implants have been considered the gold standard for their treatment [1, 2]. In the majority of cases, alveolar ridge atrophy in both vertical and horizontal dimensions happen almost immediately after tooth extraction [3, 4], resulting in the loss of alveolar ridge width and height [5, 6]. Implant placement in sites with severe vertical and/or horizontal atrophies remains one of the most challenging procedures in dentistry [7]. Bone augmentation techniques are demanded in order to improve esthetic outcomes and long-term prognoses for dental implant treatment [8, 9]. The need for bone augmentation increases with age, particularly in cases involving traumatic injury or pre-existing bony defects [10].

A major challenge in regeneration of large bony defects is “soft tissue matrix” collapse which results in resorption and migration of the bone graft. Proper surgical management of the expanded soft tissue volume is essential to prevent resorption of graft material by maintaining a space between the periosteum and the underlying bone [11]. Guided bone regeneration (GBR) is a surgical procedure in which membranes act as a barrier to maintain a space which allows the cell growth of the adjacent bone into the space and form new bone; soft tissue will be excluded from the augmented area [12, 13]. GBR aims to regenerate the alveolar bone of atrophic arches and reconstruct large osseous defects of jaws [14]. Space maintenance can be accomplished in different ways using titanium-reinforced non-resorbable membranes (i.e., polytetrafluoroethylene (PTFE) membranes), titanium meshes, bone blocks and even dental implants [15–20]. GBR has become a widely accepted approach; however, achieving and maintaining a stable regenerative space under soft tissue pressure continues to be a critical challenge [21, 22].

Tenting/tent-pole technique for space maintenance was first described by Fugazzotto in 1993, who used a mixture of alloplastic and freeze-dried homologous graft on the alveolar ridge defect, stabilized with standard tenting screws in order to preserve the spatial structure [23]. The tent-pole technique can be performed by either wide-head tenting screws or normal-sized-head screws placed vertically, horizontally or even obliquely above the augmented site; placed with a considerable distance from the defect to allow for the over-grafting of bone particles/materials to reach maximum bone gain. This technique will implement an umbrella effect above the augmented site to maintain the shape and size of the augmentation by minimizing graft mobility and protecting the woven bone from external soft tissue pressure. Tenting screws are commonly applied, being technically simple and well tolerated by patients [11, 13, 24–30]. In this open technique, after insertion of tenting screws into the defect, the bone graft is then packed around the screws followed by covering the defect with a resorbable collagen membrane [31, 32]. Moreover, the tent-pole technique can also be applied by using wide-head tent-pole abutments instead of conventional healing/temporary abutments in dental implants; in these cases, which are mostly one-staged procedures, the head of these tent-pole abutments are placed 2–4 mm above the head of the implant and will act like a tenting screw that has been inserted vertically [33].

Although many original studies have investigated the efficacy of tenting screws/tent-pole technique in alveolar ridge augmentations, to the best of the authors’ knowledge, there are no recent and comprehensive systematic reviews on this subject in the literature. The current systematic review and meta-analysis was designed and executed in order to gather all human randomized and non-randomized studies that have used tenting/tent-pole screws as the sole space-maintenance technique in vertical and/or horizontal alveolar ridge augmentations; if the results of tenting technique were compared against conventional GBR, those comparisons were also considered.

## Methods and materials

This systematic review was conducted in accordance with the Preferred Reporting Items for Systematic Reviews and Meta-Analyses (PRISMA) 2020 guidelines [34]. A detailed protocol was registered in the PROSPERO database under the registration number of CRD420251178321. The research question was established using the PICO framework as follows: how does tenting screw/tent-pole technique (I), in comparison with other conventional methods of bone regeneration (C), affect vertical and/or horizontal ridge augmentation success/failure (O) in human patients with alveolar bone loss (P)?

### Eligibility criteria

Studies were considered eligible based on the predefined PICO inclusion criteria:

- *Population*: Adult human individuals with vertical and/or horizontal alveolar ridge atrophies.
- *Intervention and Outcomes*: Tenting screw utilization in alveolar ridge augmentation and short- (< 12 months) and/or long-term (> 12 months) evaluation of their success/failure.
- *Comparison*: Bone augmentation outcomes compared between tenting screws and other techniques of augmentation.
- *Study Design*: All types of randomized and non-randomized original human clinical studies except for case reports/series with less than 10 patients. Tenting screws integrated into the abutment of dental implants were also considered. Combinations of tenting screws with bone particles, platelet-rich fibrin/plasma (PRF/PRP), collagen membranes and similar adjuncts were also included. However, studies using components that prevent soft tissue collapse, such as bone blocks or titanium mesh were excluded, as the aim of this systematic review was to specifically investigate the effect of tenting screws as space maintainers.

### Search strategy and data sources

An electronic search was carried out across PubMed, Scopus, Embase, Web of Science and Cochrane for studies published up until April 02nd, 2026. No publication date limit was implemented. Only English-language publications were included. The search terms and queries used for each database are detailed in Table 1.

**Table 1:** Search queries

Database	Date	Search Query	Results
PubMed	April 02nd, 2026	(tenting screw) OR (tent pole) OR (space maintaining screw) OR (tent screw) OR (tenting pole)	313
Web of Science	April 02nd, 2026	TS=(“tenting screw” OR “tent pole” OR “space maintaining screw” OR “tent screw” OR “tenting pole”)	70
Scopus	April 02nd, 2026	TITLE-ABS-KEY(“tenting screw” OR “tent pole” OR “space maintaining screw” OR “tent screw” OR “tenting pole”)	119
Embase	April 02nd, 2026	(‘tenting screw’:ti, ab, kw OR ‘tent pole’:ti, ab, kw OR ‘space maintaining screw’:ti, ab, kw OR ‘tent screw’:ti, ab, kw OR ‘tenting pole’:ti, ab, kw)	75
Cochrane	April 02nd, 2026	(“tenting screw” OR “tent pole” OR “space maintaining screw” OR “tent screw” OR “tenting pole”)	22

### Study selection

Two reviewers (BS and AY) independently screened titles and abstracts according to the inclusion criteria. Full texts were then checked in details. Any conflicts were resolved by the judgement of a third reviewer (RA).

### Data extraction

Two reviewers (BS and AY) independently extracted and organized the included studies. Discrepancies between reviewers were addressed through consensus or third-party adjudication (RA). Extracted data items were as follows: author, publication year, study type, sample size, participant demographics (i.e., gender, age), aim, study groups, defect site and size, procedure, tenting screw and dental implant characteristics (i.e., shape, length, and cervical width, etc.), evaluation methods and periods, outcomes (e.g., bone regeneration, implant survival rates, etc.).

### Quality assessment

The Cochrane ROB-2 [35] and ROBINS-I V2 [36] tools were used to assess and detect any potential risks of bias in human RCTs and non-randomized studies/cohorts, respectively. The JBI checklist [37] used for the risk of bias assessment of included case series. Two reviewers independently (BS and AY) evaluated the quality of the studies, and any disagreements were resolved by a third reviewer (RA).

### Certainty assessment

The overall certainty of the meta-evidence and recommendation was evaluated in the comparative studies using the Grades of Recommendations, Assessment, Development, and Evaluation (GRADE) approach [38].

### Data synthesis

Random-effect models with restricted maximum likelihood (REML) method were used to estimate the pooled effect sizes where at least three studies were available to be included in the analysis. I-squared indicator was utilized to evaluate the statistical between-study heterogeneity as values higher than 60% were considered substantial heterogeneity and values between 40% and 60% were considered moderate heterogeneity. Forest plots were created for the demonstration of the effects of the different included studies and the global pooled estimations. Meta-regression models were used to calculate the effect of patients’ age and gender, defect sizes, and evaluation period on the vertical and/or horizontal bone gain. Subgroup analysis was planned to be performed where at least two studies were available in a subgroup; hence, subgroup analyses were conducted on the evaluation period, the type of used grafting materials and membranes, the additional use of PRF/PRP, and the number of stages passed for the implant placement (one- versus two-staged procedures). Egger’s linear regression and Funnel plot were utilized to assess the potential publication bias on the bone gain after ridge augmentation. STATA^®^ 17.0 software (StataCorp LP, Lakeway Drive, College Station, TX, USA) was utilized to conduct all analyses. P-values lower than 0.05 were considered significant.

## Results

### Study selection

**Fig. 1:**
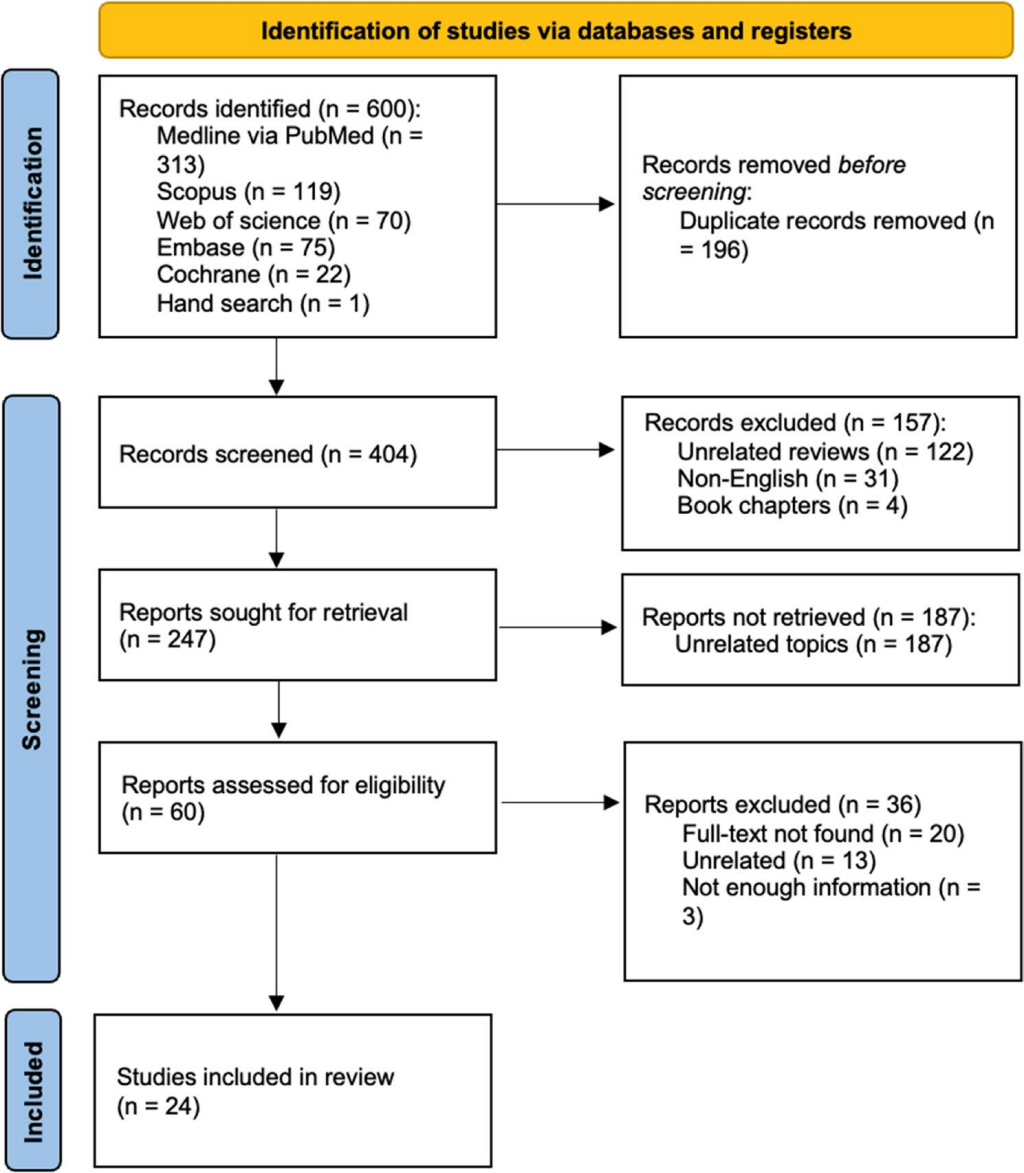
The PRISMA flowchart

The initial electronic search retrieved 600 records. Following title and abstract screening based on predefined eligibility criteria, 60 articles were selected for full-text review. Twenty-four studies met the inclusion criteria and were included in the final synthesis (Fig. 1). A comprehensive summary of each study’s design, methodology, population demographics, exposure characteristics, and outcomes is provided in Table 1. Studies were from 2002 up to 2025: 2002 (*n* = 1) [11]; 2010 (*n* = 1) [32]; 2012 (*n* = 1) [39]; 2015 (*n* = 1) [40]; 2016 (*n* = 1) [41]; 2017 (*n* = 2) [24, 42]; 2019 (*n* = 1) [43]; 2020 (*n* = 5) [44–48]; 2021 (*n* = 2) [49, 50]; 2022 (*n* = 1) [51]; 2023 (*n* = 2) [52, 53]; 2024 (*n* = 4) [54–57]; and 2025 (*n* = 2) [33, 58].

### Study characteristics

#### Study design

Included studies consisted of 4 randomized clinical trials (RCTs) [40, 45, 48, 51], 13 cohorts [11, 32, 33, 39, 41, 42, 49, 50, 53–57], and 7 non-randomized interventional studies [24, 43, 44, 46, 47, 52, 58] (Supplementary Table 1).

#### Demographics

A total of 909 patients were evaluated; in some studies with a total of 111 patients, the gender ratio was not specified and in the rest of the studies 275 males and 523 females with ages ranging from 18 to 81 years old were included. Except for two studies [24, 42], the average of patients’ age were reported as follows; 29.95 [47], 35.1 [55], 36.78 ± 13.9 [41], 36.9 [52], 37.88 ± 13.19 [58], 40.7 ± 13.3 [45], 41.9 [53], 48.2 [57], 50.06 [32], 50.2 ± 12.6 [40], 50.92 ± 13.01 [48], 52.08 ± 5.84 [50], 52.3 ± 5.9 [51], 52.4 [33], 54.14 ± 14.73 [44], 55 [56], 56.8 ± 7.8 [49], 60.59 ± 11.36 [43], 60.96 ± 11.73 [46], 62 [39], and 65 years old [11]. One study separately reported mean ages as 52.32 ± 15.11 years for men and 56.71 ± 13.06 years for women [54].

#### Study groups

Seventeen studies did not compare tenting structure with other methods [11, 32, 33, 39–43, 46, 49, 50, 53–58]. In the remaining seven studies, screw tent-pole technique was compared against other augmentation methods, which included the following materials: autogenous bone block with L-PRF membrane [48]; customized ceramic membrane [51]; standard screw with i-PRF agglutinated DBBM and PRF membrane [45]; tunnel and open PTFE techniques, both consisting of a mixture of mineralized FDBA and particulate bovine-derived hydroxyapatite, with the open PTFE technique additionally using a titanium-reinforced PTFE membrane [24]; Bio-Oss bone substitutes, autogenous bone block and collagen membrane [52]; conventional GBR, including DBBM and particulate autogenous bone with collagen membrane [44]; in situ bone ring using autogenous bone block with absorbable collagen membrane and a mixture of xenograft bone mineral graft and autogenous blood [47].

Among these studies, a variety of materials were utilized in conjunction with screws within the tenting screw groups including: L-PRF membrane and a mixture of particulate allograft with i-PRF [48]; particulate nano bone graft (Alloplast) and collagen membrane [51]; PRF membrane and a mixture of I-PRF agglutinated DBBM and I-PRF [45]; resorbable collagen membrane and a mixture of mineralized FDBA and particulate bovine-derived hydroxyapatite [24]; Bio-Oss bone substitutes, autogenous bone particle and collagen membrane [58]; DBBM, particulate autogenous bone and resorbable collagen membrane [44]; a mixture of autogenous blood and xenograft bone mineral graft [47].

#### Alveolar defect

##### Defect type

Ten studies included only vertical defects [11, 32, 33, 39, 42, 48, 50, 51, 55, 56]. Twelve studies had horizontal defects [24, 40, 41, 43–47, 49, 52, 57, 58]. One study had both vertical and horizontal defects [53]. Additionally, one study reported vertical (*n* = 25), horizontal (*n* = 167) and combined (*n* = 36) defects [54].

##### Defect site

Defect sites of patients were as follows; posterior mandible (*n* = 6) [33, 48–51, 53], anterior mandible (*n* = 1) [39], anterior maxilla (*n* = 4) [41, 45, 47, 58] and anterior region of both mandible and maxilla (*n* = 1) [55]. Some studies gave information regarding defect location only at the jaw level (mandible/maxilla), including only mandible (*n* = 1) [11] and both maxilla and mandible (*n* = 4) [40, 54, 56, 57]. Five studies separately reported the distribution of defects according to location (mandibular, maxillary, anterior and posterior) [32, 40, 44, 46, 52]. Overall, among these studies, seventeen reported mandibular [11, 32, 33, 39, 40, 44, 46, 48–57] and thirteen reported maxillary defects [32, 40, 41, 44–47, 52, 54–58].

##### Defect size

Fourteen studies reported diverse information on defect size including defects more than 5 mm [33, 53]; 5 mm [51], 3 mm [43, 46], 3–5 mm [50, 52], 4 mm [44, 45, 49], 3–6 mm [24]; more than 7 mm [32]; 9–11 mm [11]. One study reported a mean defect size of 7 mm ranging from 5 to 12 mm [55]. Two studies[47, 57] reported defect sizes based on the Cawood-Howell’s classification[59] including horizontal class IV defects with a defect width of 3–6 mm [47], and horizontal bone defects up to Class IV with defect sizes of 3–4 mm [57]; Class IV in the Cawood-Howell’s classification represents a knife-edge ridge form with adequate height but insufficient width [59]. Another study [58] included horizontal defects classified by Benic [60] as class 2 and 3 with defect size of 4 mm.

#### Surgical procedure

Five studies used a one-staged process; bone augmentation and dental implant placement happened at the same time [11, 33, 39, 41, 58]. The remaining nineteen studies applied a two-staged protocol for the subsequent implantation after ridge rehabilitation. The average time between the two stages were diverse as follows: 4 months (*n* = 1) [49], 4–5 months (*n* = 1) [32], 4–8 months (*n* = 1) [52], 6 months (*n* = 3) [40, 47, 48], 9 months (*n* = 1) [45], 9.3 months (*n* = 1) [55] and 10 months (*n* = 1) [53]. One study reported a 6 months gap for some groups and 9 months for other groups based on the kind of graft material [54]. Nine studies did not report an exact time between the two steps [24, 42–44, 46, 50, 51, 56, 57].

#### Tenting screws

Five studies reported the total number of tent-pole structures; two of them used 493 tenting screw [45, 54] and 3 studies used 517 dental implant abutments as tenting structures (11,39,58). Seventeen studies gave information on the brand of screws which were as follows; Meisinger BTX100 Screw System [48], Salvin Dental Specialties [43], Antonhib [50], Neodent (*n* = 4) [44, 45, 52, 58], Artfix Implants [49], Stryker [57], Pro-fix (*n* = 3) [24, 40, 47], WY Biomedical Co [56], Ustomed-Schirmschrauben, Geistlich [54], Biotem Implant [33], KLS Martin, Jacksonville, FL [32] and LeForte system, Jeil medical [55]. Three studies reported the brand of dental implants used as tent-pole including; Straumann (*n* = 2) [39, 58], Xive (Friadent GmbH) [39], Biotem Implant [33], Nobel [58], and Astra [58].

Regarding the size of tent-pole structures, head diameter of screws was reported in ten studies including: 1.5 mm [52, 58], 3 mm [56], 3.5 mm [44], 3.84 ± 0.02 mm [45], 4 mm, 5 mm [54], 6 mm [53, 54], 8 and 10 mm [53], 6–8 mm [33] for screws; and 3.8 mm [39], 4.1 mm [39], 4 mm [11] for tent-pole abutments. Fourteen studies gave information about length of structures which were as follows: 1.2 mm [55], 6 mm [43, 46], 7 mm [45, 53], 7.9 mm [50], 8 mm and 10 mm [44, 54], 9 mm [51, 53], 11 mm [53], 12 mm [54], 13 mm [53, 54], 15 mm [53], 7–10 mm [52, 58], 12–14 mm [33], 5–12 mm [57] for screws; and 2 mm [33, 58], 12–14 mm and 13 to 15 mm [39], 15 mm [11] for tent-pole dental implants and/or their abutments.

#### Complementary augmentation materials

A wide range of materials were used alongside screw tent-pole technique in non-comparative studies which were as follows: deproteinized bovine bone mineral (DBBM) and absorbable collagen membrane [53]; particulate mineralized freeze-dried bone allograft (FDBA) and bovine xenograft with resorbable collagen membrane or acellular dermal matrix alone in some cases or alongside adjunctive biologic agents (rhPDGF-BB or PRP) in other cases [46]; mineralized FDBA, particulate bovine-derived hydroxyapatite xenograft, allograft and resorbable collagen membrane or acellular dermal matrix or bovine collagen sponge with synthetic particulate graft in patients with and without use of steroids [43]; resorbable collagen membrane, L-PRF membrane and a mixture of xenograft bone substitute material and segmented PRF membrane [49]; buffy coats, particulate allograft (DFDBA), resorbable collagen membrane and A-PRF membrane (autologous fibrin membrane) [57]; allograft FDBA and acellular dermal matrix GBR membrane alone or with particulate autogenous bone in some cases [40]; cross-linked collagen membrane with bone substitutes including mineralized allograft (FDBA) or xenograft or a mixture of allograft and xenograft (DBBM) [56]; native collagen membrane, a mixture of A-PRF membrane with I-PRF, and particulate graft material including autogenous bone alone, or autogenous and xenogeneic bone, or allogenic max graft [54]; resorbable collagen membrane and a mixture of particulate mineralized allograft and autogenous blood [32]; a mixture of beta-tricalcium phosphate synthetic bone graft and autogenous blood [42]; particulate mineralized allograft bone and resorbable xenogeneic pericardium membrane [41]; cross-linked collagen membrane and a combination of autogenous bone and DBBM [55].

Four studies used implants as tent-pole: in two of them only implants were used as tenting structures but other components were also used including autogenous cancellous bone and Tissel fibrin glue [39]; autogenous bone and PRP [11]. In one study, tenting implants were used in normal edentulous ridges, wide head tenting screws were applied in the most severe defects where implant placement was avoided to prevent inferior alveolar nerve injury, and other materials were as follows: sticky bovine bone, resorbable collagen membrane, concentrated growth factor (CGF) membrane and sticky autogenous bone consisting of autogenous bone and CGF-derived fibrin glue [33]. Another study compared conventional GBR to tenting screw technique, with both groups having healing abutment as tent-like support; The GBR group consisted of DBBM, particulate autogenous bone and collagen membrane; and the tenting screw group applied the exact same material with the addition of tenting screws [58]. One study applied mini screws, alloplastic bone graft, PRF membrane and Hypro-Sorb absorbable collagen membrane [50].

#### Dental implants

In general, eleven studies reported the exact number of dental implants which was a total of 1,039 implants [11, 32, 39, 40, 42, 48, 49, 52–54, 58]. Data on implant brands were available in nine studies and were as follows: Bio3 Implants [48], Zimmer (*n* = 2) [32, 49], Straumann (*n* = 4) [32, 39, 47, 58], Xive (Friadent GmbH) [39], Biotem Implant [33], BioHorizons (*n* = 2) [32, 55], Nobel [58], Astra (*n* = 3) [32, 54, 58], and MIS [55].

### Outcomes

#### Complications

Seventeen studies reported complications [11, 32, 33, 39–45, 47–49, 51, 52, 55, 58]; including healing (*n* = 15) [11, 32, 33, 39–45, 49, 51, 52, 55, 58], surgical (*n* = 2) [47, 48] and dental implant complications (*n* = 4) [11, 39, 41, 58]. Among them, only two cases were reported to be excluded of the study because of graft loss [50].

#### Bone gain

The reported mean vertical bone gain ranged between 1.72 mm and 10.2 mm. One study reported percentage of height changes. The horizontal bone gain ranged from 2.35 ± 1.64 mm to 4.72 ± 2.06 mm. The detailed reported bone gains and their confidence intervals (CI) will be further displayed in the meta-analyses section.

### Quality assessment

**Fig. 2:**
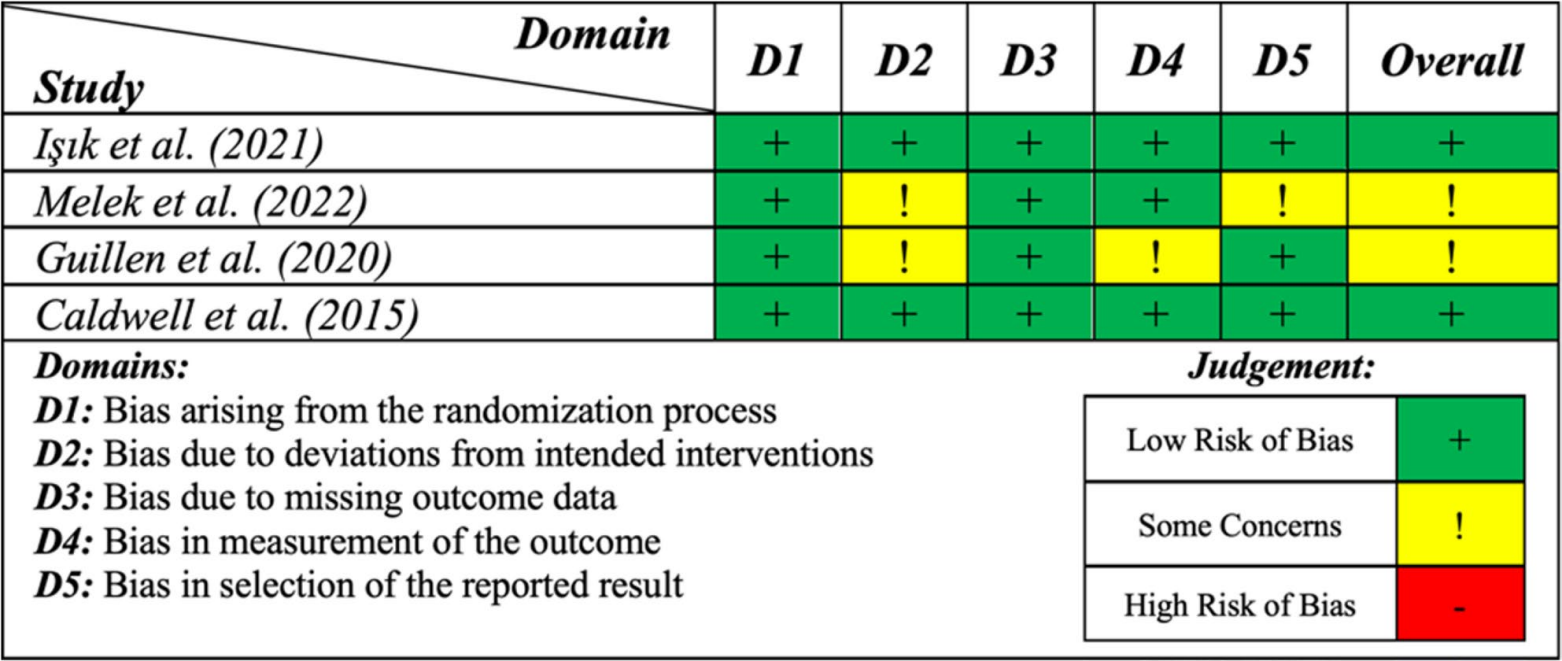
The ROB-2 assessment tool for randomized studies

**Fig. 3:**
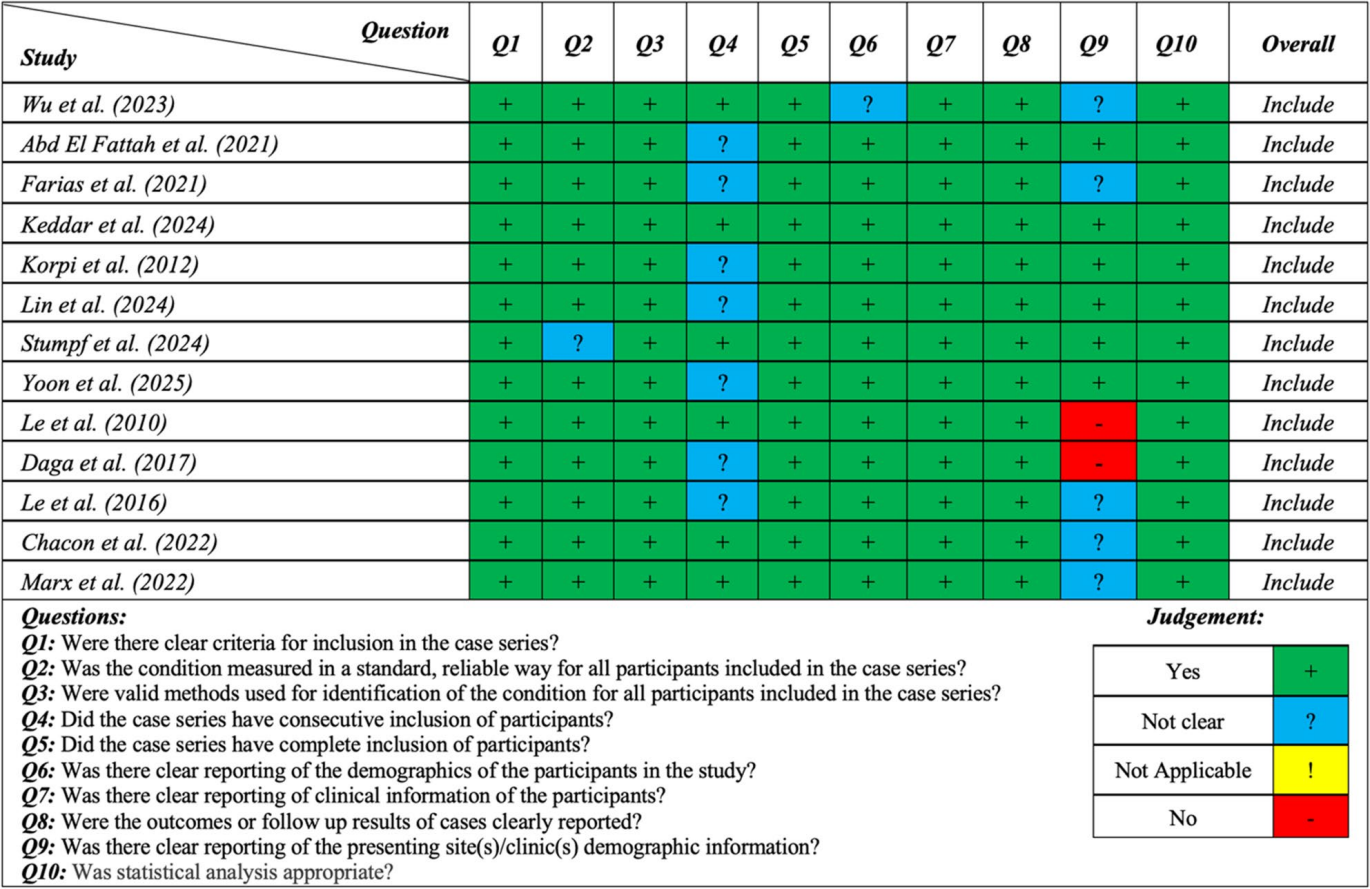
The JBI checklist for case series

**Fig. 4:**
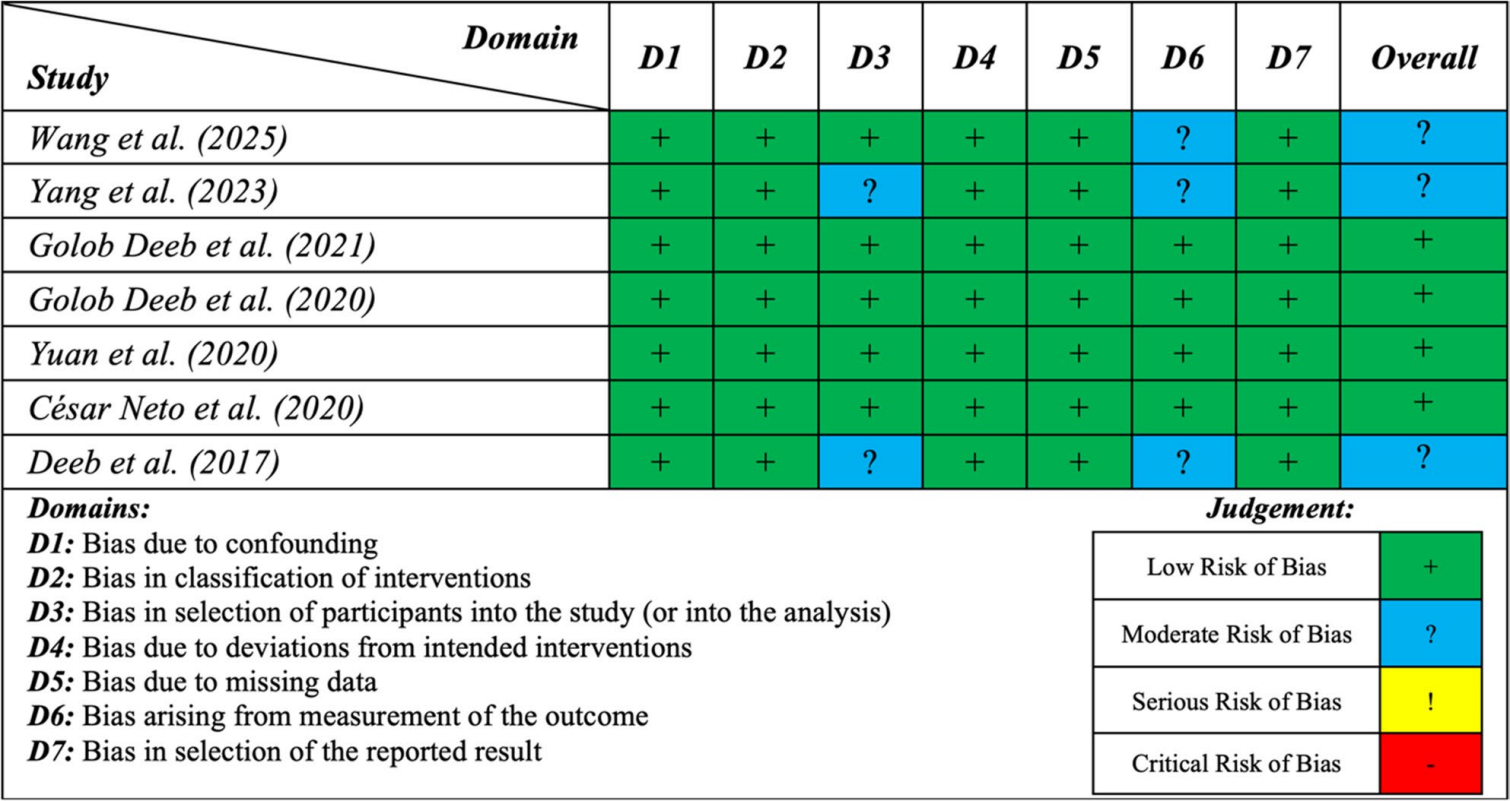
The ROBINS-I V2 assessment tool for non-randomized and cohort studies

Amongst the four randomized clinical trials, the ROB-2 assessment showed 2 of the studies to have low risks of bias while the other two had some minor concerns (Fig. 2). The JBI checklist was used for 13 case series and all of the studies met the minimum bias assessment eligibility to be included in this systematic review (Fig. 3). The ROBINS-I V2 tool was used for 7 cohorts and non-randomized clinical studies and 3 of them had an overall moderate risk of bias while the other 4 had no signs of overall bias (Fig. 4).

### Certainty assessment

The report of the GRADE assessment for the 5 cohort studies that had compared the outcomes of tenting screws/tent-pole technique versus conventional GBR (with no bone blocks) regarding horizontal alveolar bone gain showed a moderate level of certainty due to no signs of serious risk of bias, inconsistency, or indirectness; however, there were serious signs of imprecision (Supplementary Table 2).

### Meta-analysis

#### Vertical bone gain

**Fig. 5:**
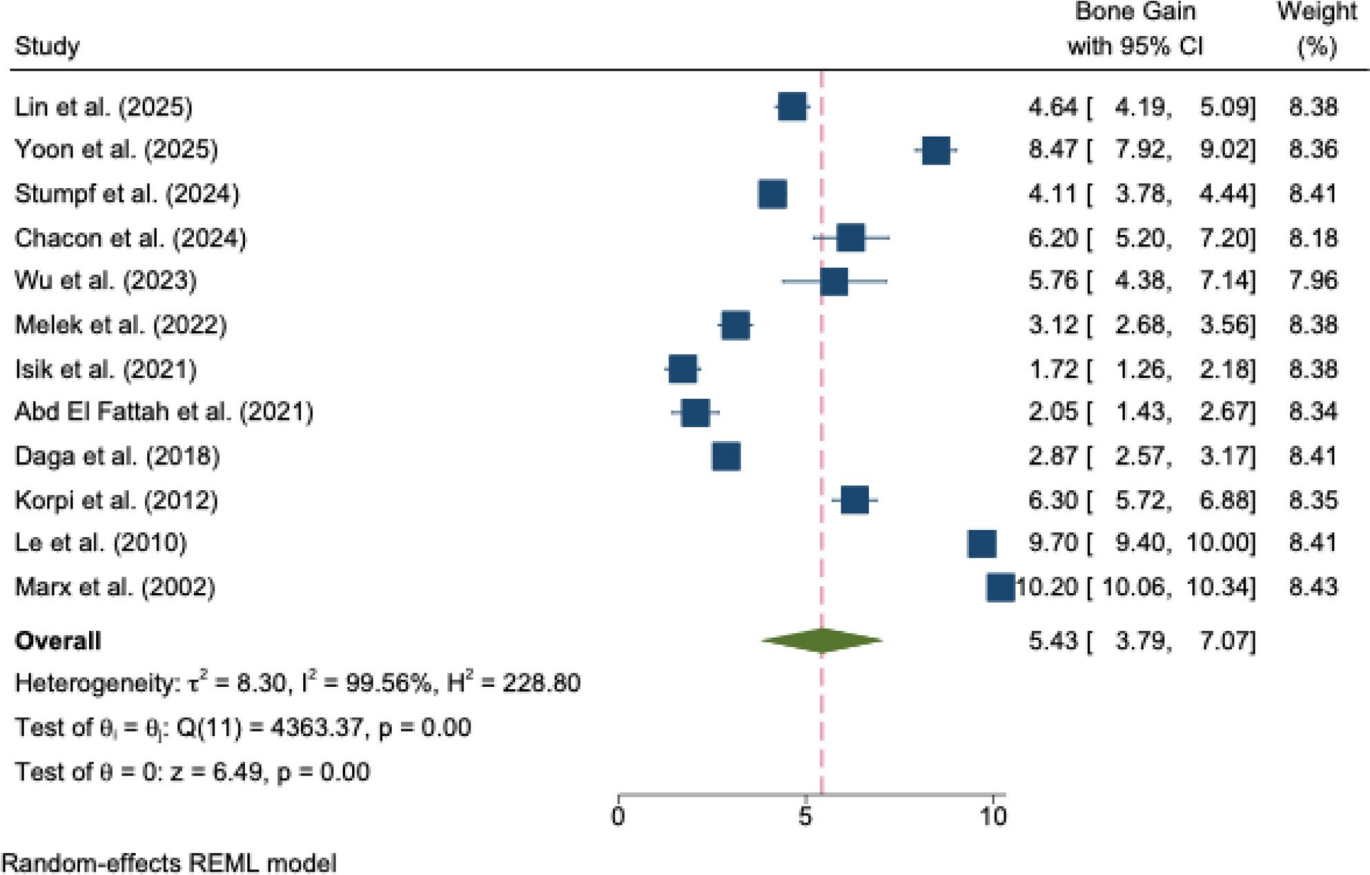
Means of vertical bone gain using tenting screws

**Fig. 6:**
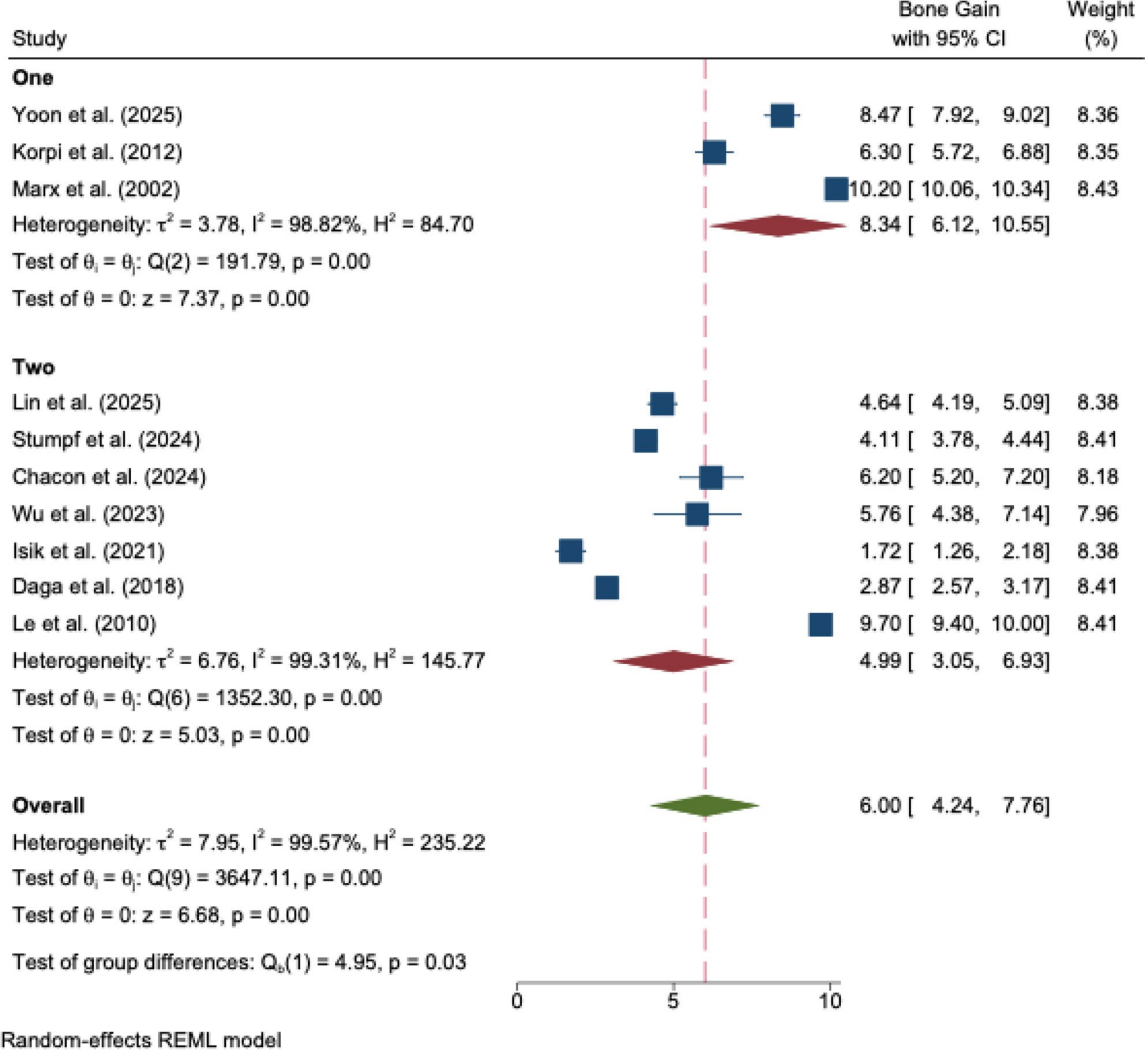
Means of vertical bone gain using tenting screws based on the number of surgical stages

**Fig. 7:**
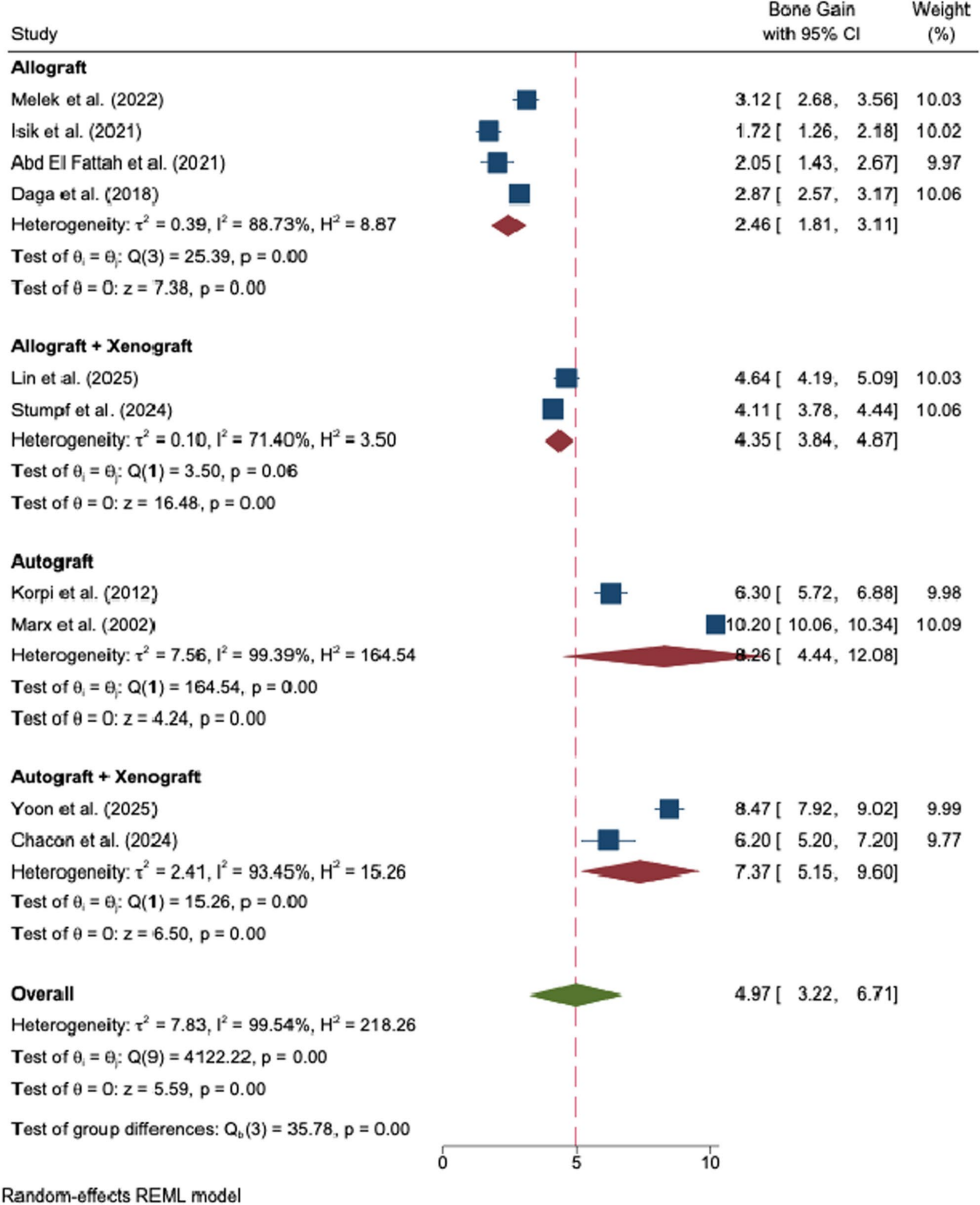
Means of vertical bone gain using tenting screws based on the type of graft used

Twelve studies [11, 32, 33, 39, 42, 48, 50, 51, 53–56] were selected for further analysis regarding vertical alveolar bone gain using tenting screws based on the initial defect size, number of defect sites and final means and SDs of vertical bone gain measurements in mm. Overall, the results showed that the mean vertical bone gain was at 5.43 mm (95% CI: 3.79–7.07, *P* = 0.00) (Heterogeneity: τ² = 8.30, I^2^ = 99.56%) (Fig. 5). The random-effects meta-regression showed no significant correlations for age and gender with vertical bone gain (*P* = 0.31, and *P* = 0.06, respectively); whereas defect size (Coefficient: 1.05, 95% CI: 0.99–1.12) and evaluation periods (Coefficient: 0.39, 95% CI: 0.04–0.73) were both significant indicators for vertical bone gain (*P* = 0.00, and *P* = 0.027, respectively). Based on the number of surgical stages, vertical bone gain was significantly higher in one-staged procedures (*P* = 0.03) (Heterogeneity: τ² = 7.95, I^2^ = 99.57%) at 8.34 mm (95% CI: 6.12–10.55) compared to two-staged procedures at 4.99 mm (95% CI: 3.05–6.93) (Fig. 6). Based on the type of bone particle grafting used in the procedures, using allografts alone had the significantly weakest vertical bone gain at 2.46 mm (95% CI: 1.81–3.11) (*P* = 0.00) (Fig. 7). Autograft alone (8.26 mm, 95% CI: 4.44–12.08) and autograft + xenograft (7.37 mm, 95% CI: 5.15–9.60) both significantly outperformed allograft alone (2.46 mm, 95% CI: 1.81–3.11) and allograft + xenograft (4.35 mm, 95% CI: 3.84–4.87) in means of vertical bone gain (*P* = 0.00) (Heterogeneity: τ² = 7.83, I^2^ = 99.54%) (Fig. 7).

**Fig. 8:**
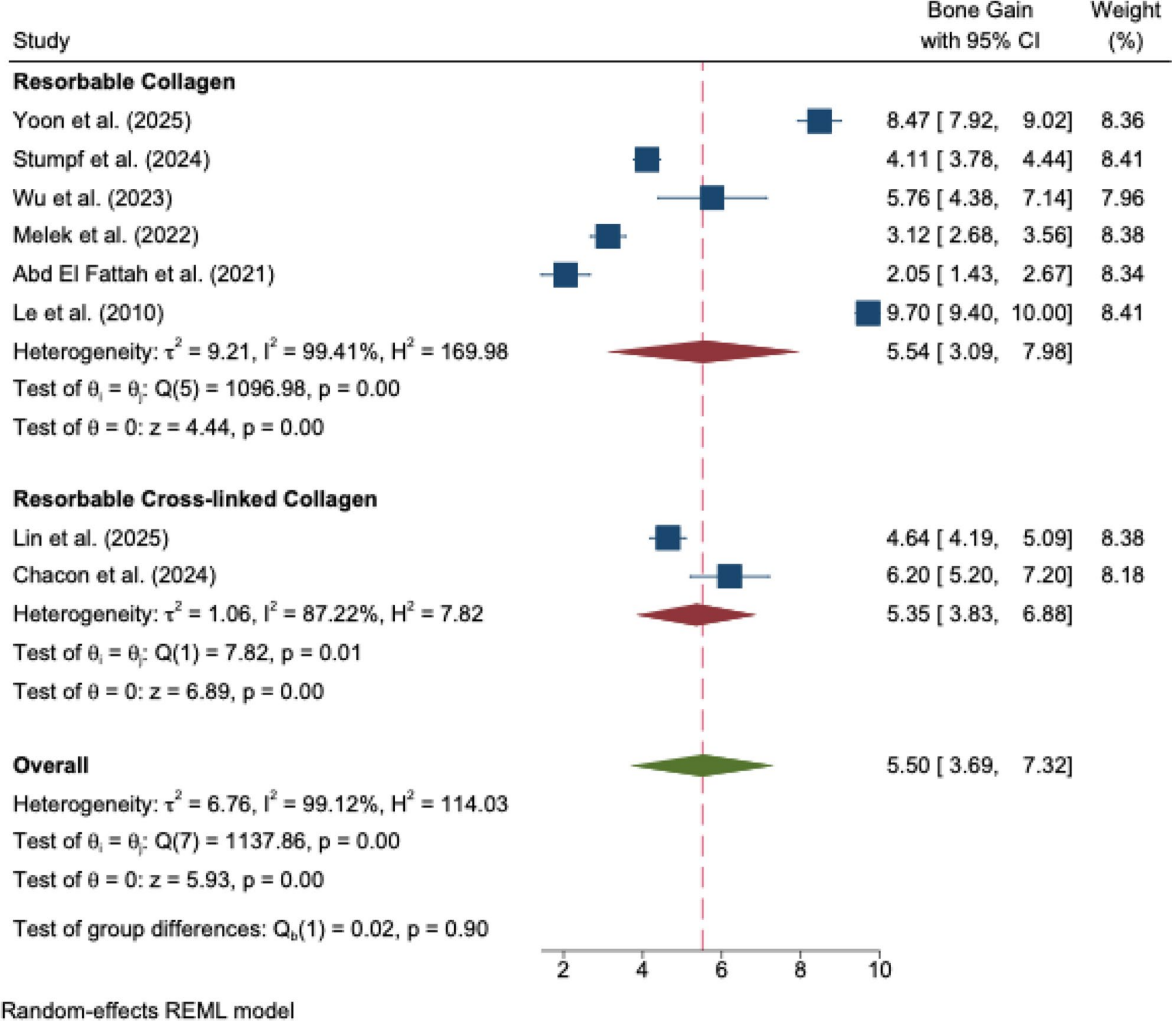
Means of vertical bone gain using tenting screws based on the type of collagen membrane

**Fig. 9:**
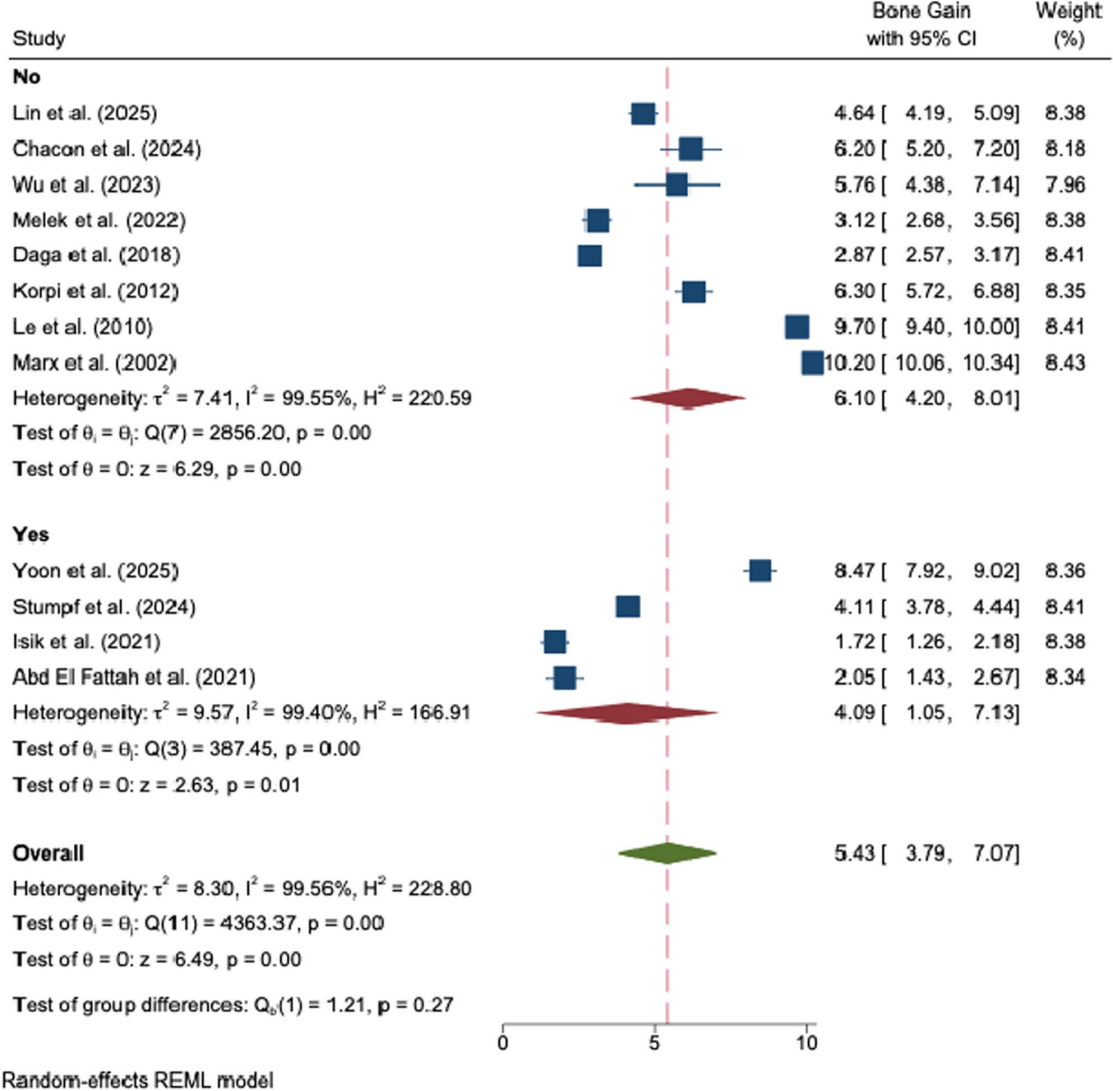
Means of vertical bone gain using tenting screws based on using complementary PRF/PRP

**Fig. 10:**
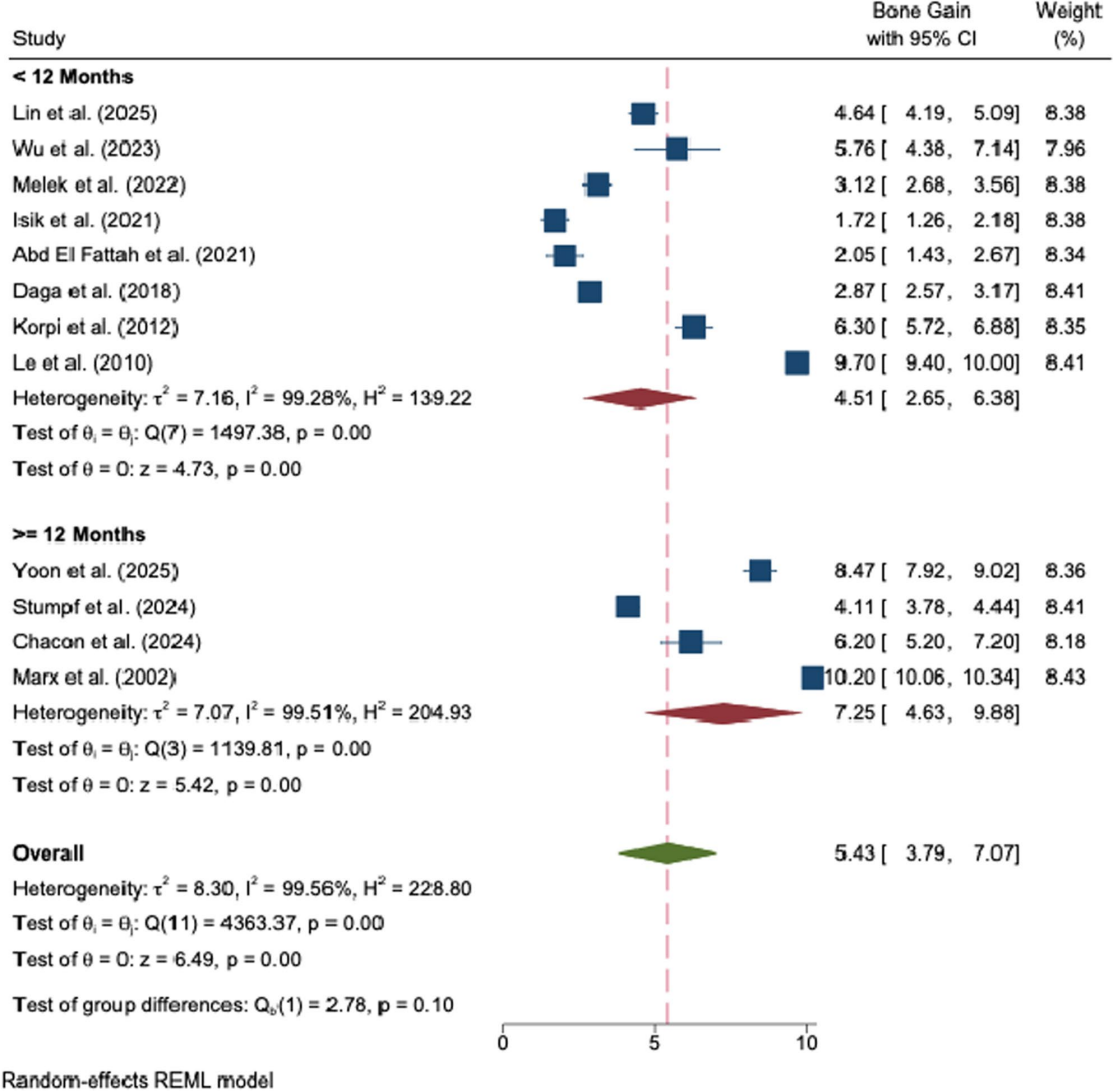
Means of vertical bone gain using tenting screws based on the evaluation period

In regards to using collagen membranes on top of the augmented site, there were no significant differences in the means of final vertical bone gain between using non-cross-linked (5.54 mm, 95% CI: 3.09–7.98) and cross-linked resorbable collagen membranes (5.35 mm, 95% CI: 3.83–6.88) (*P* = 0.90) (Heterogeneity: τ² = 6.76, I^2^ = 99.12%) (Fig. 8). Regarding using complementary PRF/PRP in vertical bone augmentation, there were no significant difference between using (4.09 mm, 95% CI: 1.05–7.13) and not using them (6.10 mm, 95% CI: 4.20–8.01) (*P* = 0.27) (Heterogeneity: τ² = 8.30, I^2^ = 99.56%) (Fig. 9). Based on the evaluation period, there were no significant differences in the final vertical bone gains reported in the first 12 months (4.51 mm, 95% CI: 2.65–6.38) or after the initial 12 months post-operation (7.25 mm, 95% CI: 4.63–9.88) (*P* = 0.10) (Heterogeneity: τ² = 8.30, I^2^ = 99.56%) (Fig. 10).

##### Publication bias

**Fig. 11:**
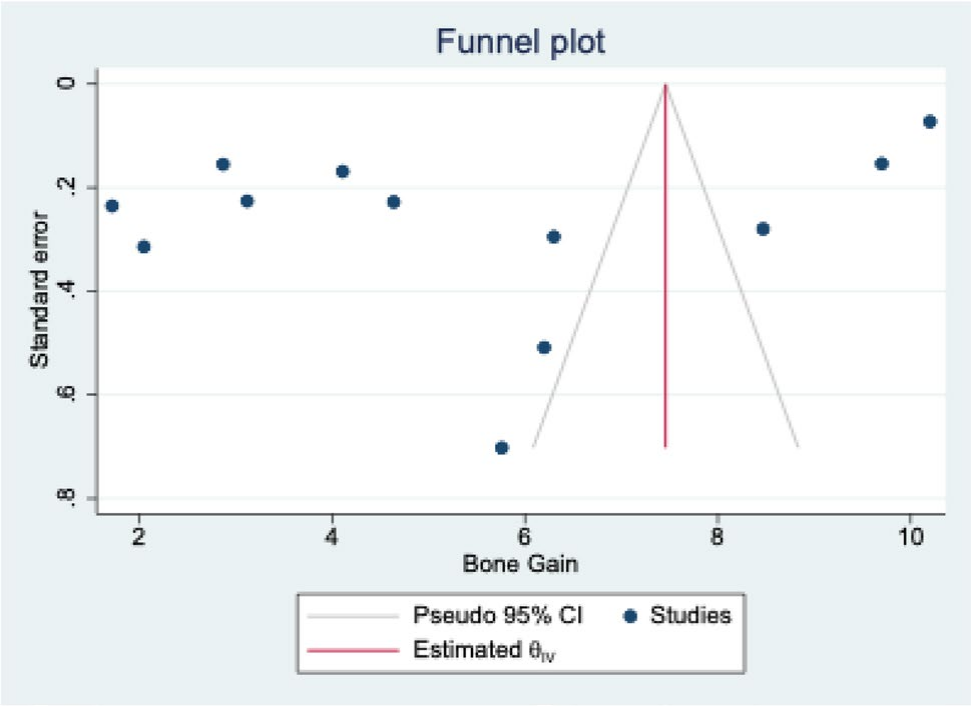
Funnel plot of standard error in studies selected for vertical bone gain using tenting screws

There were no signs of publication bias observable in selected studies for assessment of tenting screws/tent-pole technique in vertical alveolar ridge augmentations (*P* = 0.75) (Fig. 11).

#### Horizontal bone gain

**Fig. 12:**
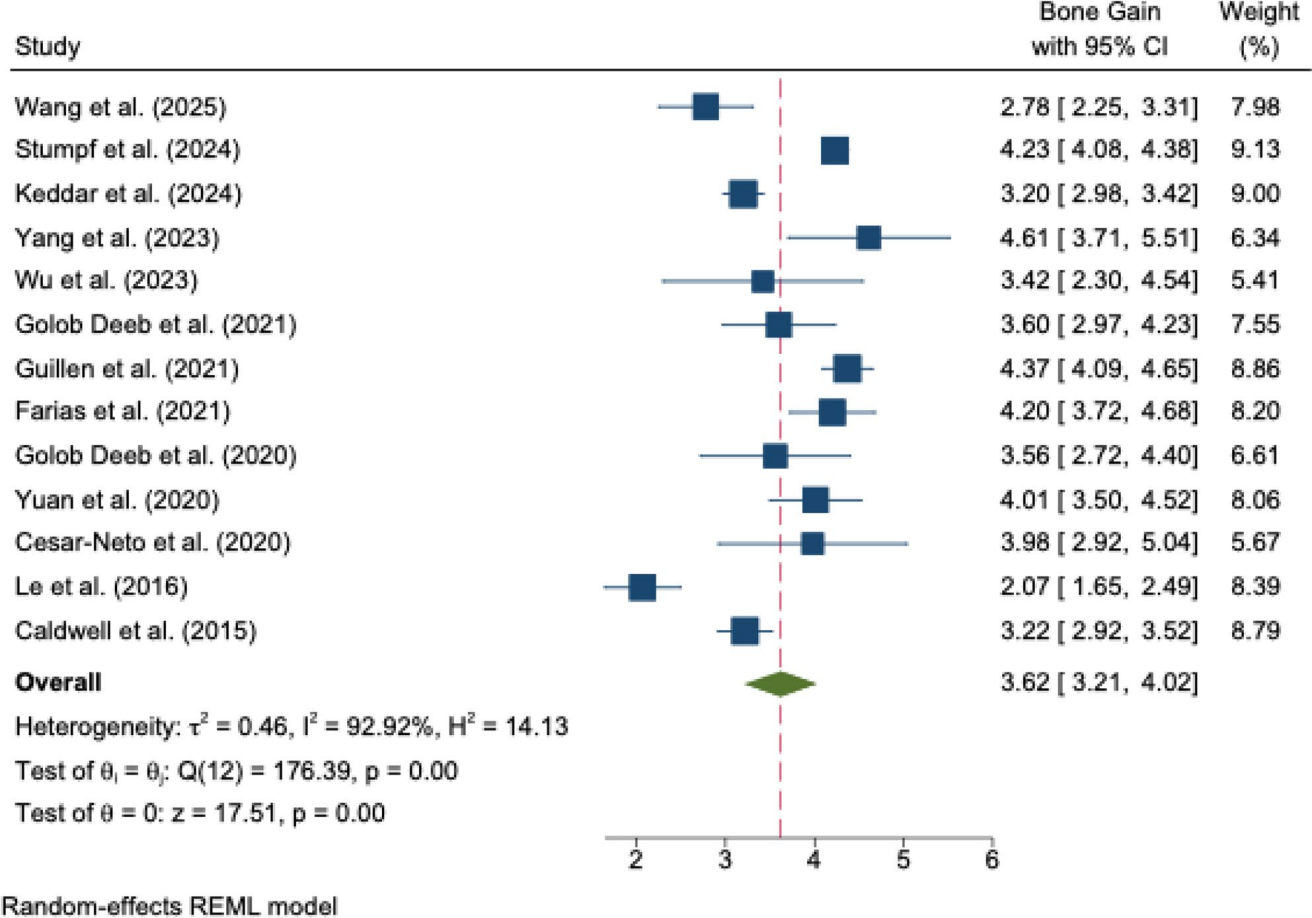
Means of horizontal bone gain using tenting screws

**Fig. 13:**
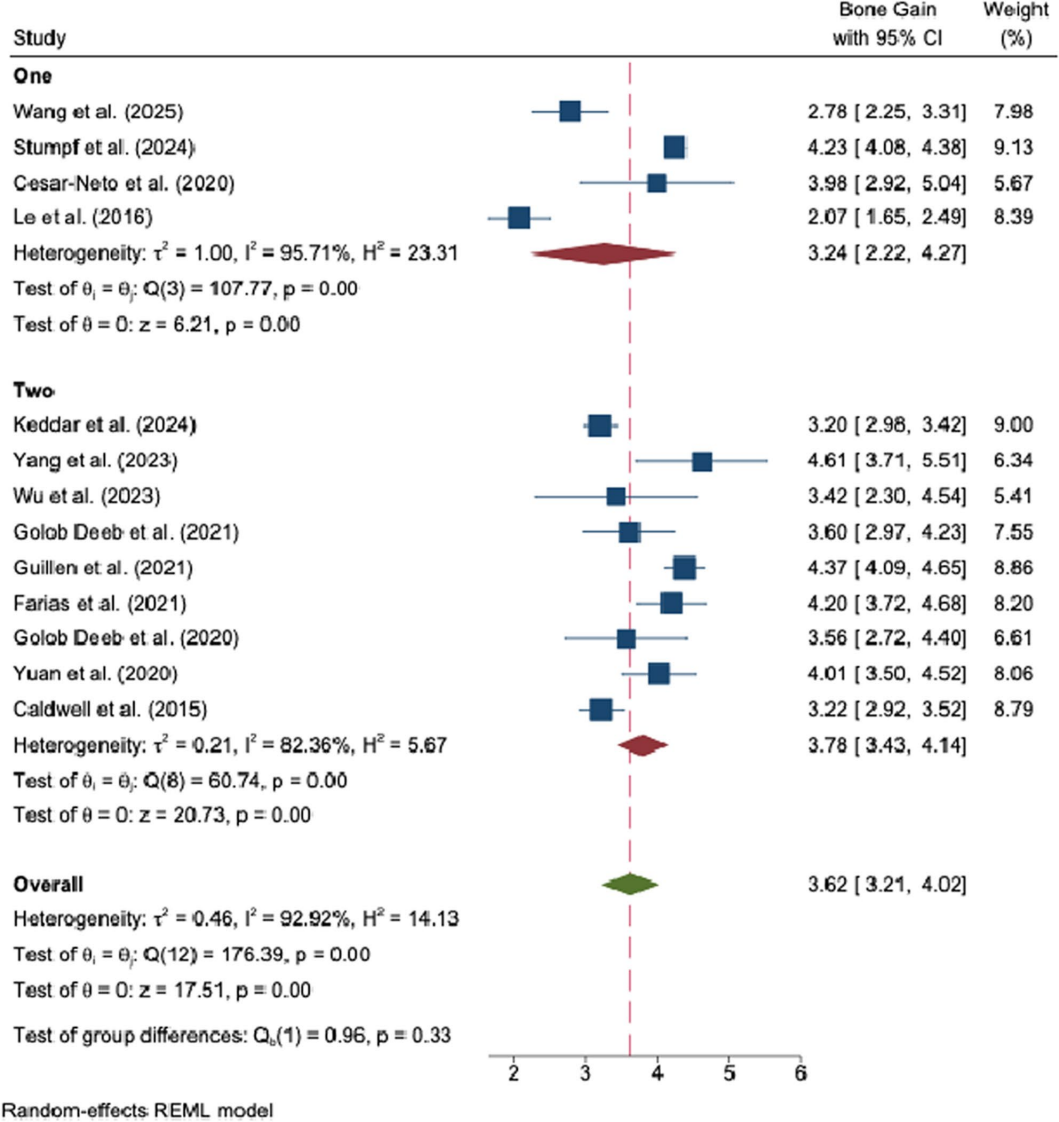
Means of horizontal bone gain using tenting screws based on the number of surgical stages

**Fig. 14:**
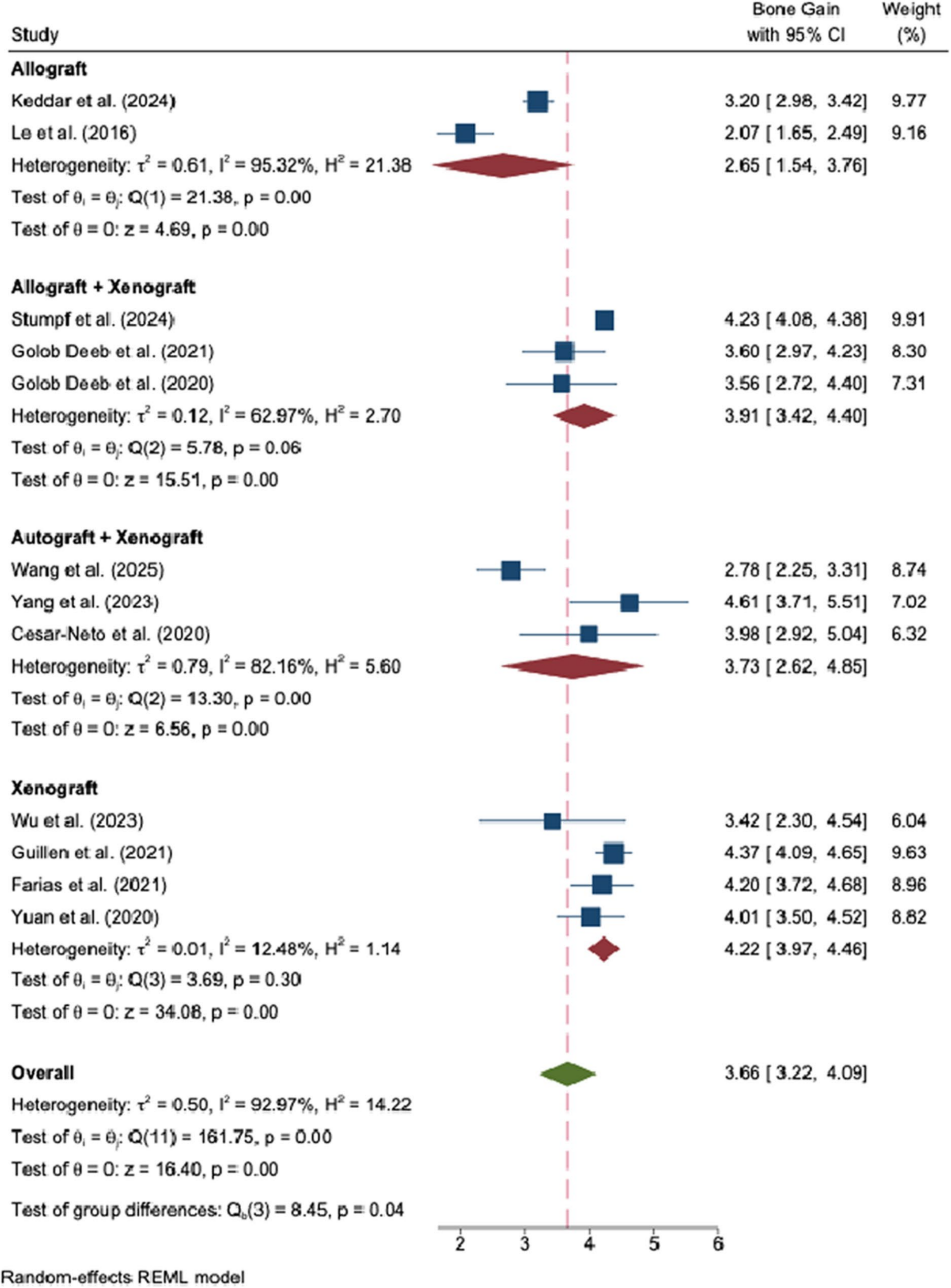
Means of horizontal bone gain using tenting screws based on the type of graft

Thirteen studies were selected for analysis on the means of horizontal bone gain using tenting screws [40, 41, 43–47, 49, 52–54, 57, 58]. The overall mean horizontal bone gain was 3.62 mm (95% CI: 3.21–4.02) (*P* = 0.00) (Heterogeneity: τ² = 0.46, I^2^ = 92.92%) (Fig. 12). The random-effects meta-regression showed no significant correlation for age, gender and evaluation period with horizontal bone gain (*P* = 0.50, *P* = 0.75, and *P* = 0.10, respectively); however, defect size (Coefficient: 0.51, 95% CI: 0.30–0.72) was a significant indicator (*P* = 0.00). There were no significant differences in horizontal bone gains between one-staged (3.24 mm, 95% CI: 2.22–4.27) and two-staged surgical procedures (3.78 mm, 95% CI: 3.43–4.14) (*P* = 0.33) (Heterogeneity: τ² = 0.46, I^2^ = 92.92%) (Fig. 13). Regarding the type of bone particles used in the horizontal augmentation, xenografts (4.22 mm, 95% CI: 3.97–4.46) led to significantly higher horizontal bone gain compared to allografts (2.65 mm, 95% CI: 1.54–3.76) (*P* = 0.04) (Heterogeneity: τ² = 0.50, I^2^ = 92.97%) (Fig. 14).

**Fig. 15:**
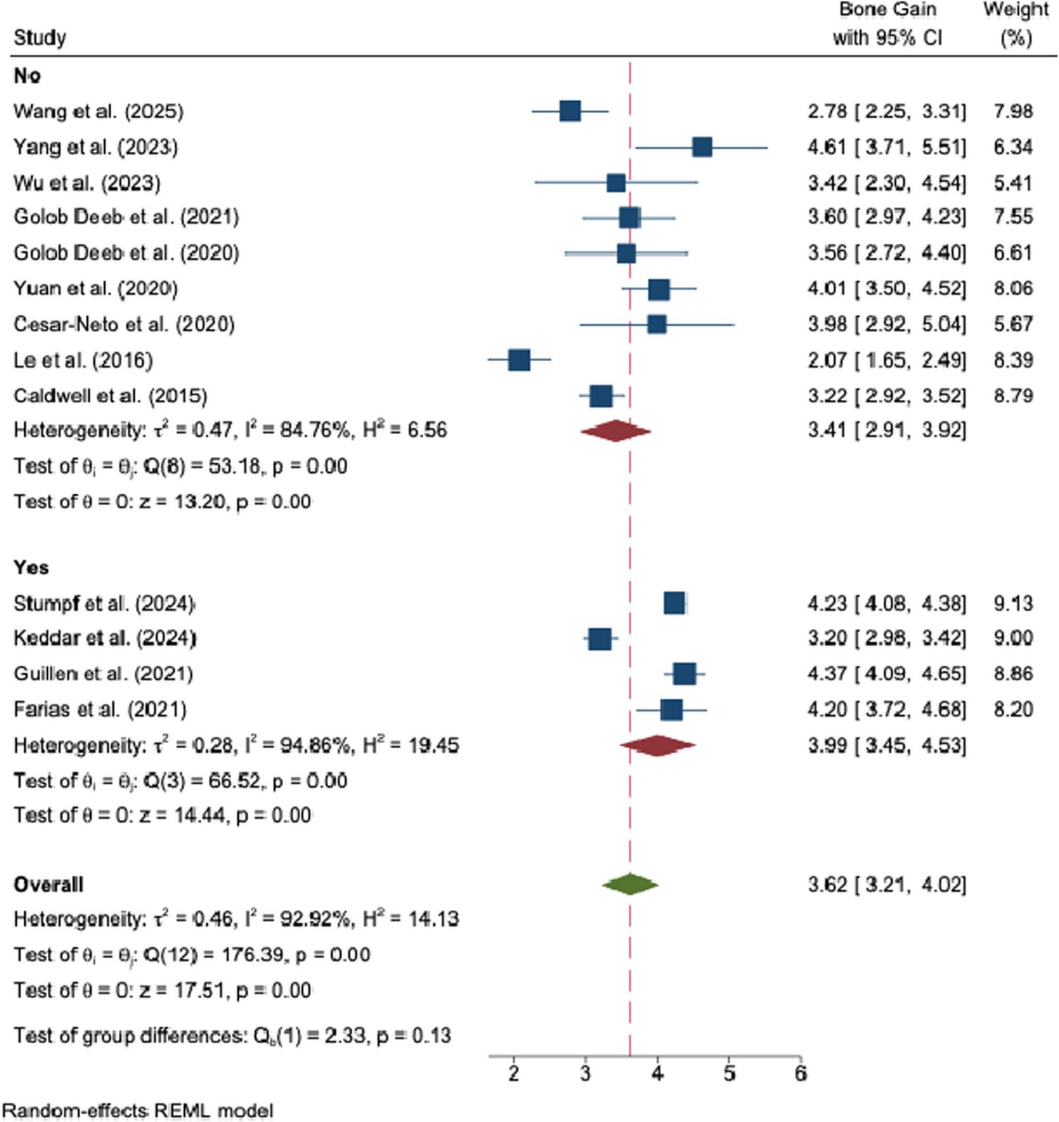
Means of horizontal bone gain using tenting screws based on using complementary PRF/PRP

**Fig. 16:**
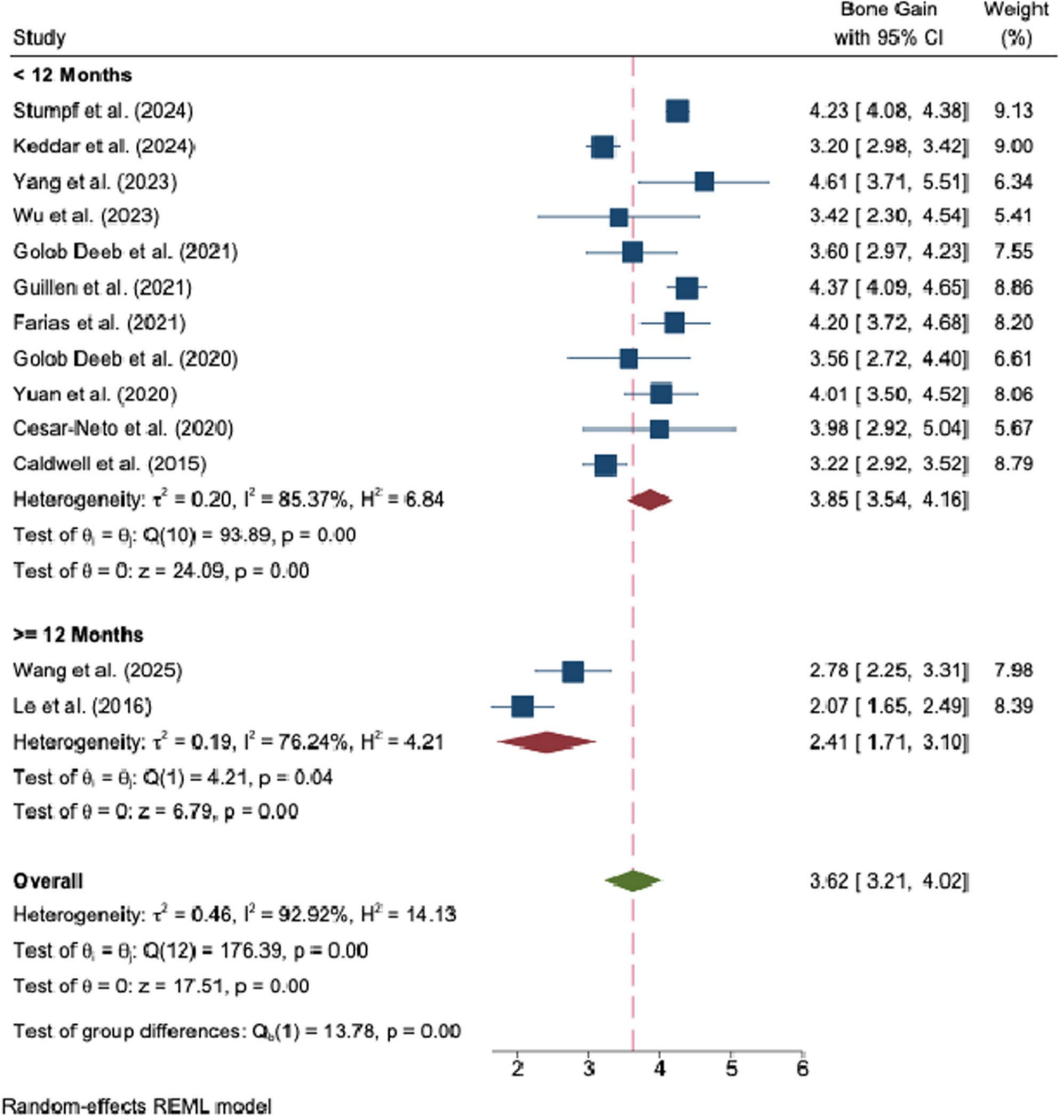
Means of horizontal bone gain using tenting screws based on the evaluation period

In regards to using complementary PRF/PRP in horizontal augmentations, horizontal bone gain was not significantly different when PRF/PRP was used (3.99 mm, 95% CI: 3.45–4.53) versus when it was not used (3.41 mm, 95% CI: 2.91–3.92) (*P* = 0.13) (Heterogeneity: τ² = 0.46, I^2^ = 92.92%) (Fig. 15). Based on the time the bone gain was reported, horizontal bone gain was significantly higher in the first 12 months (3.85 mm, 95% CI: 3.54–4.16) of observation compared to after the initial 12 months post operation (2.41 mm, 95% CI: 1.71–3.10) (*P* = 0.00) (Heterogeneity: τ² = 0.46, I^2^ = 92.92%) (Fig. 16).

##### Publication bias

**Fig. 17:**
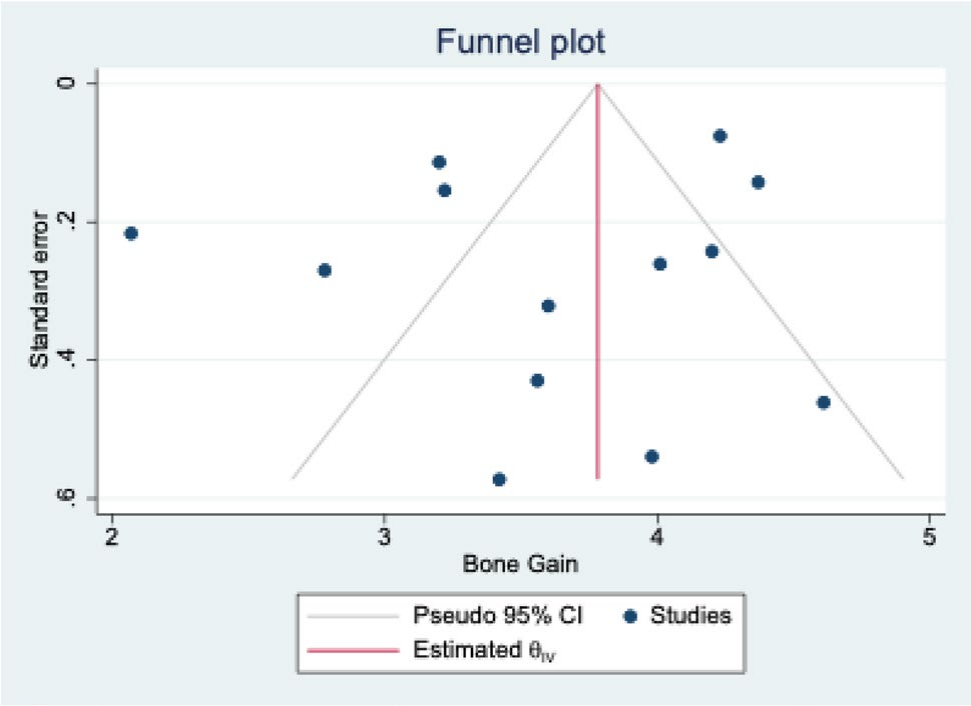
Funnel plot of standard error in studies selected for horizontal bone gain using tenting screws

There were no signs of publication bias amongst studies selected for meta-analysis using the tenting technique for horizontal reconstruction (*P* = 0.75) (Fig. 17).

#### Tenting technique vs. conventional GBR

**Fig. 18:**
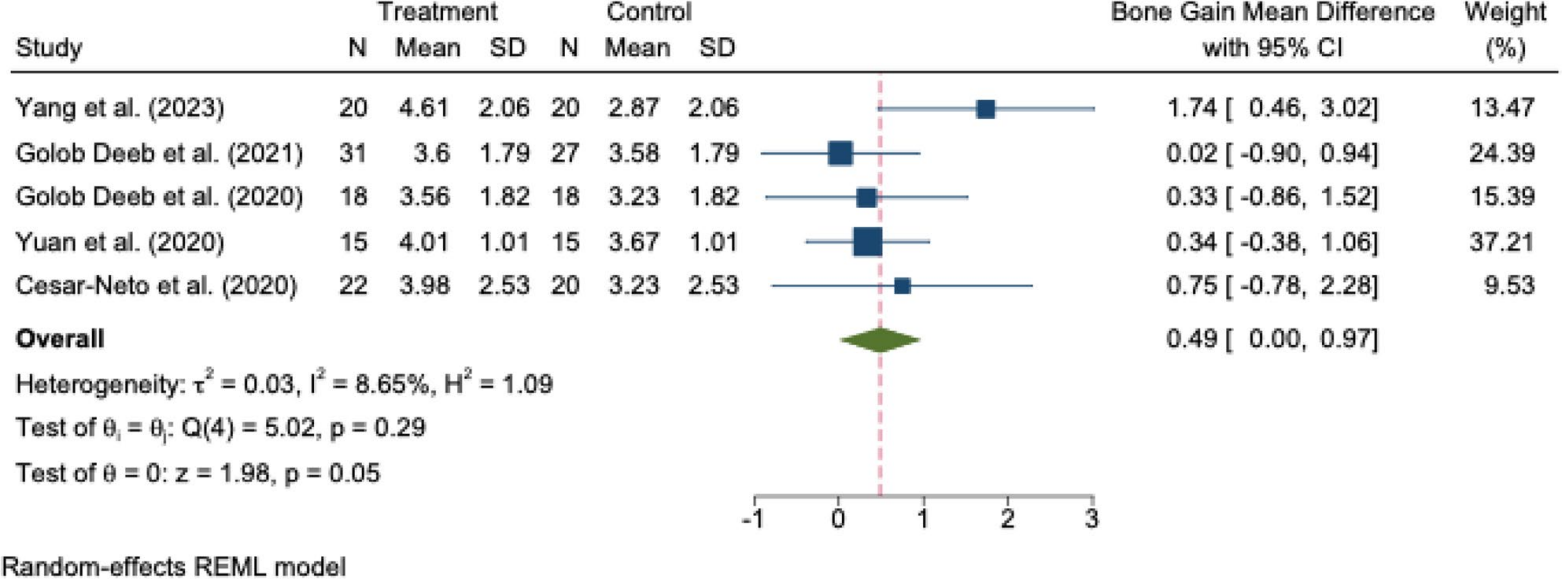
Means of horizontal bone gain in studies comparing tenting screws to conventional GBR

**Fig. 19:**
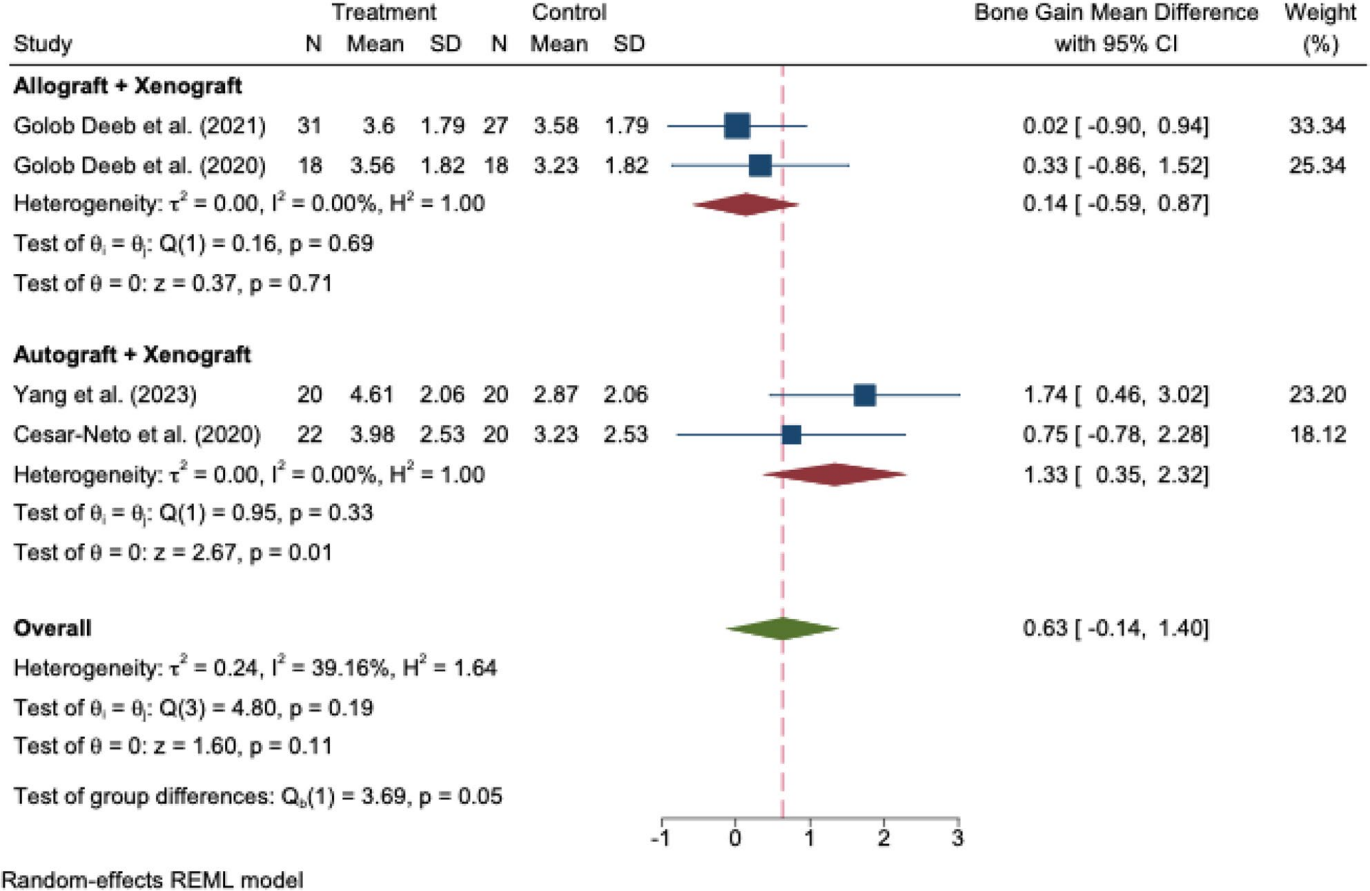
Means of horizontal bone gain in studies comparing tenting screws to conventional GBR, based on the type of graft

In 5 of the included studies, horizontal bone gain was compared between two groups [43, 44, 46, 47, 52]; (A) horizontal alveolar augmentation using tenting screws, and (B) horizontal augmentation using conventional GBR using bone particles (with no grafted bone block) and conventional titanium screws. The final horizontal bone gain difference between the two groups was statistically significant at 0.49 mm (95% CI: 0.00–0.97) with a mean effect favoring the tenting screw/tent-pole technique group (*P* = 0.05) (Heterogeneity: τ² = 0.03, I^2^ = 8.65%) (Fig. 18). Regarding the differences in final horizontal bone gain based on the type of bone particles used, there were no significant differences between allograft + xenograft, and autograft + xenograft (95% CI: -0.14–1.40, *P* = 0.05) (Heterogeneity: τ² = 0.24, I^2^ = 39.16%) (Fig. 19).

##### Publication bias

**Fig. 20:**
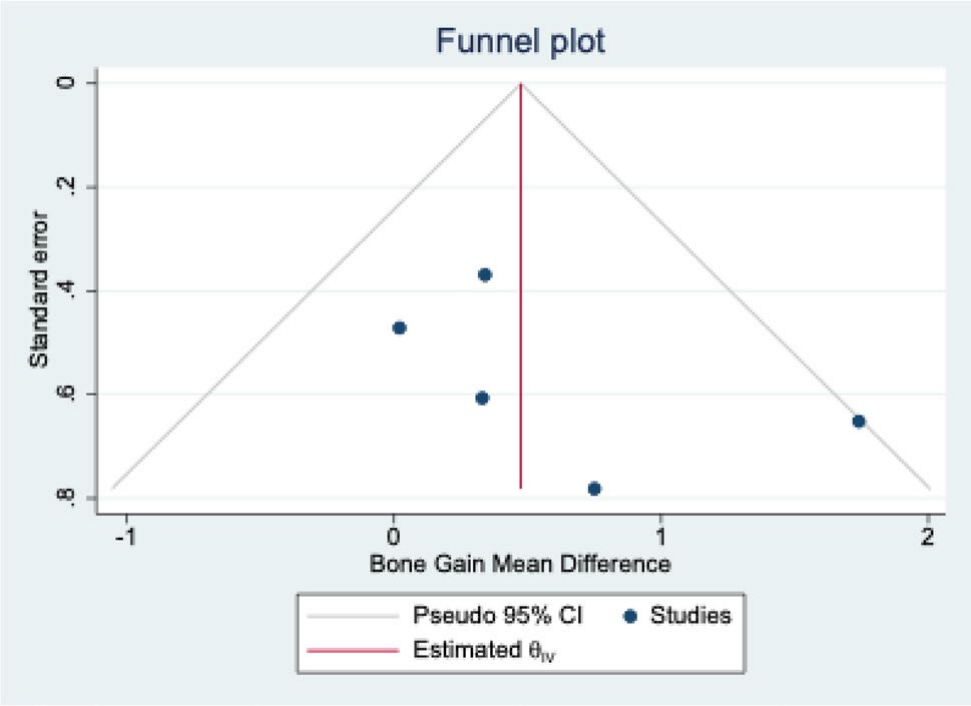
Funnel plot of standard error in studies selected for horizontal bone gain differences in comparison of tenting screws versus conventional GBR

There were no signs of publication bias amongst the 5 studies that had compared the tenting technique against conventional GBR in horizontal reconstruction (*P* = 0.23) (Fig. 20).

## Discussion

This systematic review and meta-analyses were designed and executed in order to evaluate the effects of using tenting screws and tent pole techniques in both vertical and horizontal alveolar ridge augmentations. The last time a systematic review was conducted on this topic was back in 2017 with only 13 studies included from more than a decade ago and without executing a meta-analysis [61].

**Fig. 21:**
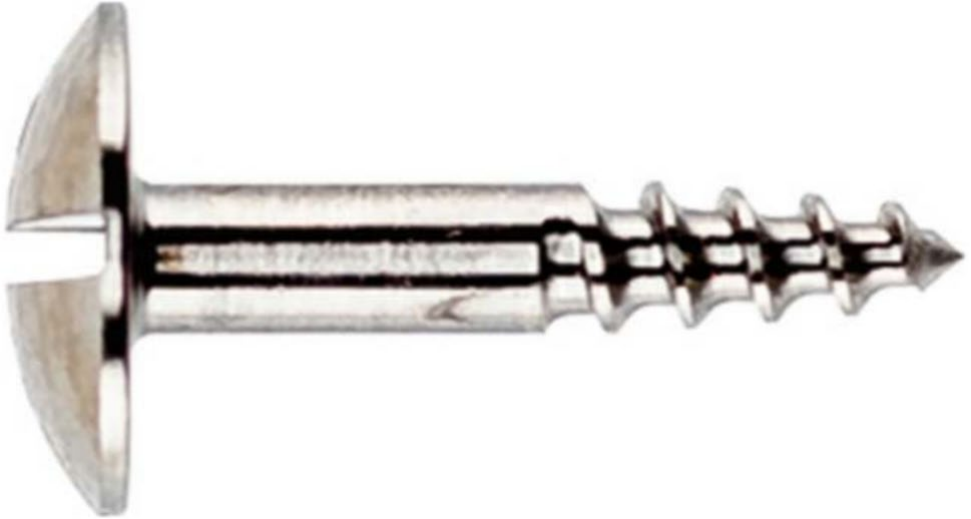
The anatomy of a wide-head tenting screw. Original image borrowed from Abdullrahman et al. from their open-access study titled “Tent-pole technique for alveolar ridge width preservation with a compromised buccal plate: a prospective cohort study” [63]

**Fig. 22:**
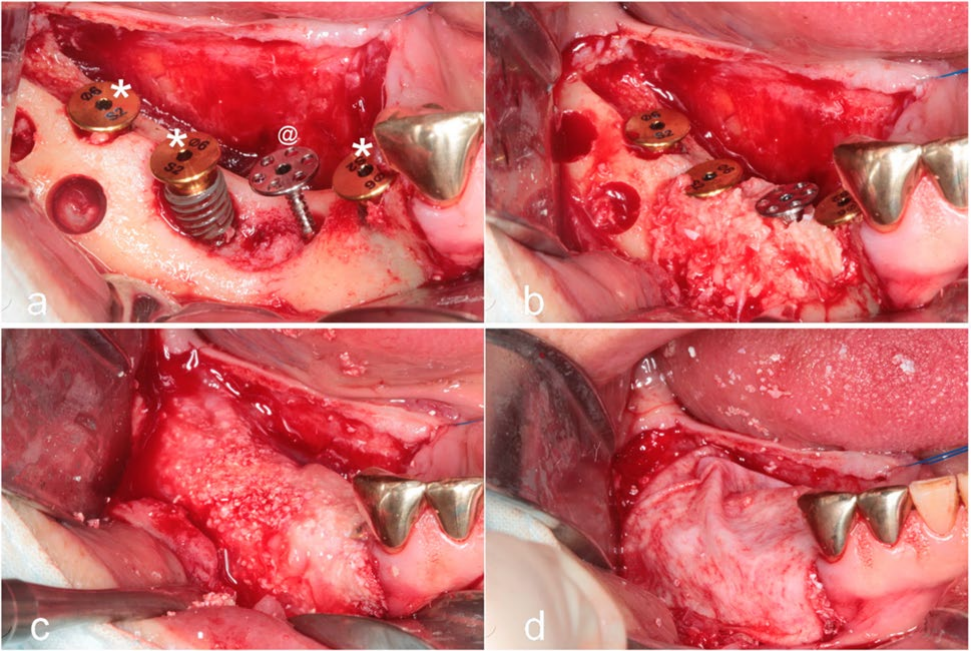
The simultaneous use of wide-head tenting screws (6 mm wide × 12 mm long) (@) and wide-head tent-pole implant abutments (*) in alveolar ridge augmentation (**a**). A combination of sticky autogenous bone material and sticky bovine bone was applied (**b** and **c**). A resorbable collagen membrane was placed on top of the augmented site without the help of any membrane tacks or fixation sutures (**d**). Original images borrowed from Yoon et al. from their open-access study titled “Retrospective Radiographic Evaluation of Ridge Dimensional Changes After Vertical Augmentation Using the Novel Wide-Head Tent Pole Screw Technique” [33]

Tenting screws, also referred to as tent-pole screws, are temporary titanium screws inserted vertically, horizontally, or obliquely inside the alveolar ridge in order to mechanically maintain a regenerative space beneath the soft tissue or membranes (e.g., collagen membranes, PTFE, etc.) during GBR procedures [62]. Ideally, with the help of complementary bone particles, new bone formation will occur up to the level of the head of these screws [62, 63]. Tenting screws have a variety of sizes in their length and the width of their head; the right size of the screws for each procedure will be determined by the depth, width and the extend of the bone defect. The head diameter for tenting screws varies from 2 to 3 mm, which is slightly wider than the shaft, up to 6–8 mm, which is significantly wider than the shaft of the screws which typically range from 1.3 to 1.8 mm [62, 63]. The tenting screws with wide umbrella-shaped heads are usually only threaded in the lower half (apical section) of their shaft (i.e., the only place that gets inserted into the augmented site) since the upper half will not have the need for insertion and its length will only contribute to space maintenance [64]. Moreover, the smooth surface of the upper half of the shaft of these screws will prevent integration [63]. The length of these screws can be up to 14 mm for severe vertical atrophies. The tent-pole technique is not entirely dependent on specificized wide-head tenting screws; it can also be performed by a number of normal-sized-head screws placed above the height- and/or width-extend of the defect in order to give the augmented site enough space to regenerate and to also prevent the collapse of the soft tissue (Fig. 21) [63]. However, the wide head of the tenting screws (i.e., 6–8 mm in head diameter) has the advantage of using a limited number of screws compared to normal-sized titanium screws (i.e., 2–3 mm in head diameter) which makes the procedure easier for both the practitioners and the patients. Additionally, some practitioners perform the tent-pole technique using the abutment (i.e., a healing abutment or a temporary abutment) of the newly-inserted dental implants as the tenting device (i.e., tenting screw) [11, 33, 39, 58]. This will create a vertical projection above the implant platform, acting like a tent-pole (Fig. 22) [33]. These tent-pole abutments, that are usually placed 2–4 mm above the head of the implant fixture, will be able to support over-grafting above the implant platform.

One of the many advantages of tenting screws, and tent-pole technique in general, is how they prevent the complexity and complications of conventional GBR techniques; the wide-head tenting screws can act like an umbrella above the augmented area with less than 10% of cases suffering from perforations and exposures in the soft tissue above; whereas titanium meshes and titanium-reinforced membranes (i.e., titanium-reinforced dense PTFE membranes) lead to tissue exposures and infections in almost 30% of cases [22, 24, 40, 42, 65, 66]. Moreover, conventional techniques are typically more difficult to execute; onlay bone block graft, which is considered the gold standard for most GBR procedures, requires to be sculpted and well-fitted on the alveolar ridge. In addition, bone blocks are prone to relatively-high resorption rates ranging from 25% to 40% within the first year, especially in cases with vertical ridge defects [67–69]. Similarly, titanium meshes and titanium-reinforced membranes, which are commonly used in augmentation procedures, need to be either customized or folded/cut to the shape of the atrophic ridge [65, 66, 70, 71]. In addition, autogenous bone blocks carry the risk of donor site morbidity that can be a limiting factor specially in older patients with significantly lower capabilities in bone regeneration and soft- and hard-tissue wound repair [72, 73].

There was a total of 24 human clinical studies included in this study; 23 of which were selected for further analysis. Of the selected studies, 10 of them had only investigated vertical bone gain, 11 had only reported horizontal bone gain, and 2 had reported both vertical and horizontal gains. Moreover, 5 of these studies had compared the outcomes of tenting screws and tent-pole technique against conventional GBR procedures (without bone blocks).

The results of the meta-analyses showed that there was an overall vertical bone gain of 5.43 mm (95% CI: 3.79–7.07) amongst studies when using tenting screws/tent-pole technique. The meta-regression showed that defect size (Coefficient: 1.05, 95% CI: 0.99–1.12, *P* = 0.00) and evaluation periods (Coefficient: 0.39, 95% CI: 0.04–0.73, *P* = 0.027) were both significant indicators for vertical bone gain. The analyses showed that one-staged procedures (bone augmentation + dental implant insertion) had significantly higher means of vertical bone gain at 8.34 mm (95% CI: 6.12–10.55) (*P* = 0.03) compared to two-staged procedures (bone augmentation first and implant insertion in the coming weeks/months) at 4.99 mm (95% CI: 3.05–6.93).

The analyses showed an overall horizontal bone gain of 3.62 mm (95% CI: 3.21–4.02) when using tenting screws/tent-pole technique. The meta-regression concluded that defect size (Coefficient: 0.51, 95% CI: 0.30–0.72, *P* = 0.00) was a significant indicator for horizontal bone gain. The analyses showed no significant differences in horizontal bone gain between one-staged (3.24 mm, 95% CI: 2.22–4.27) and two-staged (3.78 mm, 95% CI: 3.43–4.14) procedures (*P* = 0.33). The clinical decision making for choosing between one- or two-staged augmentation procedures can be based on multiple factors including: defect morphology (i.e., horizontal, vertical, or combined), risk of complications (e.g., exposure, and resorption), and soft tissue management [74]. In most cases of vertical defects, practitioners prefer the two-staged procedure due to vertical defects being more biologically demanding with higher complication rates and lower regenerative predictability; hence, most vertical augmentations (e.g., bone blocks, distraction osteogenesis, sandwich osteotomy, pedicled segmental rotation, etc.) are two-staged [75, 76]. There are multiple factors that contribute to vertical defects being more biologically demanding than horizontal defects: the lack of native bone support (on either the buccal or lingual sides) results in poor primary stability for both the grafted bone particles and implants, gravity and soft-tissue pressure which leads to graft instability, and central vascularization challenges. However, the results of this current systematic review and meta-analysis showed that tenting screws can prohibit soft tissue pressures from the augmented site, which can potentially explain the one-staged procedure significantly outperforming the two-staged procedure. On the other hand, horizontal bone augmentations are highly predictable and reliable compared to vertical defects. One-staged reconstruction procedures are widely acceptable in horizontal reconstructions due to the morphological features that allow the anchorage of both regenerative materials and dental implants inside the residual native bone (e.g., the lingual/palatal side of the horizontal defect) [77]. The results of the current meta-analyses showed no significant differences in horizontal bone gain between one- and two-staged reconstructions which is aligned with the reports in the literature; practitioners can expect decent results in one-staged horizontal reconstructions, and with the help of tenting screws/tent-pole technique they can further-prevent any soft tissue collapse and pressure on the augmented site.

In regards to the type of bone particles/nanoparticles used as regenerative materials, the analyses showed autografts (8.26 mm, 95% CI: 4.44–12.08) and autografts + xenografts (7.37 mm, 95% CI: 5.15–9.60) significantly outperformed allograft (2.46 mm, 95% CI: 1.81–3.11) and allograft + xenograft (4.35 mm, 95% CI: 3.84–4.87) in means of vertical bone gain when using tenting screws/tent-pole technique (*P* = 0.00). Moreover, the addition of xenogeneic bone particles to allogeneic bone particles (allograft + xenograft) significantly increased the mean vertical bone gain (*P* = 0.00). In horizontal reconstructions, the results of the analyses showed that xenogeneic bone particles (4.22 mm, 95% CI: 3.97–4.46) significantly outperformed allogeneic bone particles (2.65 mm, 95% CI: 1.54–3.76) (*P* = 0.04). However, there were no significant differences between allograft (2.65 mm, 95% CI: 1.54–3.76), or allograft + xenograft (3.91 mm, 95% CI: 3.42–4.40), or autograft + xenograft (3.73 mm, 95% CI: 2.62–4.85) in terms of final horizontal bone gain. A number of reports in the literature have investigated the outcome differences of autografts, allografts, and xenografts against one another and there is still no consensus as to which option is superior/inferior since no significant differences have been reported in human RCTs; even though autografts are still considered the gold standard by practitioners because of its osteogenic, osteoconductive and osteoinductive capabilities. The results of the current meta-analysis showed that autografts, with or without xenogeneic particles, significantly outperformed allogeneic particles, with or without xenogeneic particles; even though most studies in the literature were not able to report any significant differences, these results show why most practitioners still view autografts (either bone blocks or bone particles) as the gold standard. Even in long-term human studies, no significant difference was reported in both bone regeneration rates and dental implants’ success and stability [78].

In another human RCT, allogeneic and xenogeneic particles were compared against each other and no statistically significant differences were observed in dimensional changes and/or histologic outcomes in alveolar ridge augmentations [79]. However, it is worth noting that reports indicate that xenografts resorb slower and consequently, might provide a proper space maintenance for a longer period [79, 80]. The results of the current meta-analysis also showed that in horizontal reconstruction, xenogeneic particles significantly outperformed allogeneic particles which can be due to the differences in their space maintenance capabilities. One of the more interesting findings of this part of the current meta-analysis, was the fact that in vertical reconstruction, adding xenogeneic bone particles did not increase the mean vertical bone gain; meanwhile, the addition of xenogeneic bone particles to the allogeneic particles (allograft + xenograft) (4.35 mm, 95% CI: 3.84–4.87) significantly increased the vertical bone gain compared to allogeneic particles alone (2.46 mm, 95% CI: 1.81–3.11) (*P* = 0.00). The results of a human RCT showed that adding xenogeneic bone particles does not add any significant value to autografts in terms of alveolar ridge augmentations [81]; the analyses of the current systematic review are aligned with these reports. In another human histomorphometry investigation, it was concluded that there are no significant differences between autograft + allograft and autograft + xenograft in terms of final bone gain in alveolar ridge reconstruction [82].

Amongst the 12 selected studies for meta-analysis in vertical bone gain, 3 of which had not reported whether they had used complementary collagen membranes or not. In the remaining 9, 6 had used resorbable non-cross-linked collagen membranes while the other 3 had used the cross-linked version. The results of the meta-analyses showed no significant differences in terms of final vertical bone gain between non-cross-linked and cross-linked resorbable collagen membranes (*P* = 0.90). In regards to final bone gain volumes and the quality of the regenerated bone, multiple RCTs and systematic reviews in the literature suggest there are no significant differences between non-cross-linked and cross-linked resorbable collagen membranes [83–85]. However, in terms of space maintenance and mechanical behavior, the literature states that cross-linked membranes have a slower degradation, high mechanical strength, high tear resistance, and better volume stability [86, 87]. Interestingly enough, the results of the current meta-analysis showed no significant differences between these two variants; this can be interpreted by the fact that using tenting screws/tent-pole technique has such a strong space maintenance influence that the superiorities of cross-linked membranes will simply cease to exist when such strong space maintenance power (i.e., tenting screws/tent-pole technique) has also been applied in the augmented site. Regarding horizontal augmentation, all of the included studies reportedly used non-cross-linked resorbable collagen membranes, hence, a comparative meta-analysis was not feasible.

The application of PRF/PRP and its influence on final bone gain was investigated amongst included studies; in both vertical and horizontal alveolar ridge reconstructions, there were no significant differences in final bone gain when PRF/PRP was (4.09 mm, 95% CI: 1.05–7.13, and 3.99 mm, 95% CI: 3.45–4.53 for vertical and horizontal reconstructions, respectively), or was not used (6.10 mm, 95% CI: 4.20–8.01, and 3.41 mm, 95% CI: 2.91–3.92, for vertical and horizontal reconstructions, respectively) during the augmentation procedure (*P* = 0.27, and *P* = 0.13, for vertical and horizontal reconstructions, respectively). When particulate grafting (i.e., bone particles/nanoparticles), barrier membranes (i.e., collagen membranes), and rigid space maintenance (e.g., tenting screws, conventional titanium screws, titanium meshes, titanium-reinforced membranes, etc.) are all combined to execute an alveolar ridge augmentation, the key determinants of bone regenerations have all been addressed: clot stability, soft tissue exclusion, and space maintenance [88–91]. In this situation, PRF/PRP can only contribute to faster angiogenesis, improved early vascularization, and enhanced soft tissue healing [66, 92–94]. However, the literature suggests no statistically significant differences in final vertical and/or horizontal bone gain when PRF/PRP is applied adjacent to space maintenance and barrier membranes and bone grafts [93–95]; the results of the current meta-analyses are completely aligned with these reports and confirm that in complicated multi-factorial alveolar ridge augmentations, PRF/PRP have not shown any meaningful contribution to the final bone gain.

Out of the 23 selected studies for meta-analysis, most of them (*n* = 19) had reported the final measurements of their bone gains (measured through radiographies) in the 9–12 months post-operation evaluation period; whereas only 6 of the studies had reported data beyond the 12 month post-operation period (i.e., 1–3 years post-operation). The results of the analyses showed no significant differences in reported vertical or horizontal bone gains between studies reporting in the first 9–12 months post-op or in the first 1–3 years post-op (*P* = 0.10, and *P* = 0.13, for vertical and horizontal reconstructions, respectively). Most RCTs tend to report their outcomes for alveolar bone gains in the 4–9 months period, since practitioners believe the maximum bone gain happens in this window; this is also the frame in which the re-entry phase of two-staged studies gets executed since it is believed that there is more than enough newly-formed bone to support the insertion of dental implants [96, 97]. Beyond the first-12-months post-op period, there is consensus in the literature regarding bone volume decreases; in a network meta-analysis of human RCTs in 2025, it was reported that premature bone resorption ranged 6% – 44% (mean: ~ 6%) after initial healing [98].

RCT-based reports in the literature indicate a significant post-healing vertical volume reduction in alveolar ridge reconstructions [98]; however, in the current meta-analysis the differences in vertical bone volume means were not significant between studies reporting in the first 9–12 months (4.51 mm, 95% CI: 2.65–6.38) and studies with reports beyond the 12 months period (7.25 mm, 95% CI: 4.63–9.88) (*P* = 0.10). The reason for significant changes in vertical bone volume beyond the 12 month-period is mainly due to initial pressure put on top of the augmented area by the soft tissue; which in case of tenting screws/tent-pole technique this initial pressure on the augmented site was significantly reduced. Hence, the grafted particles/nanoparticles were able to integrate into the residual bone walls. In most conventional vertical GBRs, a considerable portion of the grafted particles will not be able to integrate into the original bone and will be left out in the remodeling process and will be referred to as residual bone particles. The horizontal bone gain reported significant differences between the first 9–12 months and beyond the first 12 months post-op period (3.85 mm, 95% CI: 3.54–4.16, and 2.41 mm, 95% CI: 1.71–3.10, for the 9–12 months and beyond the 12 months post-op periods, respectively) (*P* = 0.00); this is aligned with the literature in the sense that horizontal alveolar reconstructions are way more predictable and stable in the first 12 months post-op compared to years after surgery [99].

Five cohort studies among the included studies had compared the horizontal bone gain outcomes of tenting screws/tent-pole technique versus conventional alveolar ridge reconstruction (using grafted bone particles and no bone blocks, with the help of conventional normal-sized-head screws) [43–47, 52]. The meta-analysis showed a statistically significant increase of 0.49 mm (95% CI: 0.00–0.97) in horizontal bone gain when tenting screws/tent-pole technique was used compared to conventional GBR technique (with no bone blocks) (*P* = 0.05). The primary stability of the grafted bone particles is a prerequisite for the long-term success of alveolar ridge reconstruction and subsequent dental implantation [100–102]. As mentioned earlier, the main advantage of the tenting screw/tent-pole technique is its space maintenance capability and prevention of any soft tissue pressures on top of the augmented site; this technique is able to achieve this goal without causing any donor site morbidity (e.g., in bone blocks, onlay bone grafting, sandwich osteotomy, etc.), surgical complication (e.g., sandwich osteotomy, inferior alveolar nerve lateralization, pedicled segmental rotation, etc.) or healing complications (i.e., considerably lower rates of mesh and membrane exposure compared to conventional GBR using titanium meshes and titanium-reinforced membranes). Included studies have stated that this superiority in horizontal gain might be due to manipulation of tenting screws’ location. The amount of augmentation/regeneration has been correlated with the predetermined protruding distance of tenting screws; graft material resorption took place no further than the heads of these screws [32, 52, 103].

### Study limitations and suggestions

(A)  The analytical reports showed a significant superiority in vertical bone gain using the tenting technique in cases with a one-staged surgical procedure (i.e., simultaneous bone augmentation and dental implantation); however, the number of these one-staged studies were relatively limited, hence, more human RCTs are required to compare the long-term bone gain differences of one-staged and two-staged procedures.
(B)  The reports showed a significant superiority in tenting-based vertical bone gain in cases with autogenic or autogenic + xenogeneic bone particles/materials compared to allogenic or allogenic + xenogeneic bone materials; however, since most reports in the literature indicate no significant difference between grafting materials, more human RCTs are required to compare these materials in a randomized setting to reach reliable conclusions.
(C)  Most alveolar ridge GBR procedures use a combination of bone blocks and bone particles with or without the help of the tenting technique. In this current study, the combined application of tenting screws/technique with bone blocks was not considered for inclusion since the main objective was to evaluate the individual effect of the tenting technique without the help of other space maintenance techniques. The analytical reports of this study showed a significant difference between tenting technique and conventional GBR (without bone blocks) in horizontal bone gain. On the other hand, bone blocks and onlay grafting have shown reliable regenerative capabilities. Therefore, future researchers are highly recommended to compare the outcome differences of tenting technique alone versus tenting technique combined with bone blocks.
(D)  Most studies had focused on the 9–12 months evaluation period post-operation to report their final bone gains; however, as indicated in the literature, there could be a significant change in bone volume after the initial 12 months post-operation. It is highly suggested that future researchers report their bone gains and volumes both in the initial healing phase (i.e., 3–9 months post-operation) and also beyond the 12-months period to assess the changes in bone volume over time.
(E)  Five cohort studies had compared the outcome differences of tenting technique against conventional GBR (with no bone blocks); the reported analytical results showed a borderline superiority to tenting technique (*P* = 0.05), which is still statistically significant but cannot be fully relied on. Moreover, due to the non-randomized nature of these studies, future human RCTs are required to reach reliable conclusions.
(F)  Most of the included studies had chosen to report the mean changes/gains in bone volume through mean vertical and/or horizontal bone gains and did not choose to report the three-dimensional changes to bone volume over time. Hence, a meta-analysis based on the three-dimensional changes was not feasible. Three-dimensional bone volume assessments can better reflect the maintenance and stabilization influence of the tenting screws/tent-pole technique. Future researchers are highly encouraged to report both the vertical and/or horizontal changes/gains as well as the overall three-dimensional bone volume changes.
(G)  Egger’s test did not detect statistically significant asymmetry amongst the studies investigating horizontal bone gain; however, given the small number of included studies, this finding should be interpreted with caution and does not exclude the possibility of publication bias.
(H)  Differences in the underlying disease conditions, or other treatment-related factors can affect the reported gains in bone height and width and should be acknowledged as potential confounding variables. The authors of the current systematic review and meta-analysis have included all patient-related and treatment-related factors that might influence the outcomes of bone augmentation/regeneration using the tent-pole technique and tenting screws in this study. Basic factors such as age, gender, and evaluation periods were analyzed as possibly-influential factors with analytical reports in previous sections of the paper. None of the studies had reported any systemic diseases amongst their patients. Moreover, the authors tried to best categorize different treatment methods to better judge their outcomes without having to compromise any potential effect of technique-based differences between studies.

### Conclusions

The tenting/tent-pole technique using wide-head tenting screws and/or wide-head tent-pole implant abutments can result in desirable vertical and horizontal alveolar ridge augmentation results, without the help of onlay bone grafting and/or titanium meshes. The tenting technique can be executed much easier and faster with wide-head structures; however, it can also be performed with normal-sized-head screws placed above the augmented site. In horizontal augmentation, tenting technique was able to significantly outperform conventional GBR (with no bone block). More human RCTs are required to reach reliable consensus regarding the superiority/inferiority of tenting/tent-pole technique against other space maintenance techniques.

## Supplementary Information

Supplementary Material 1. Extracted data.

Supplementary Material 2. Certainty assessment for comparative studies.

## Data Availability

All of the generated data are available in the paper and its supplementary materials.

## Abbreviations

GBR: Guided bone regeneration
PTFE: Polytetrafluoroethylene
RCT: Randomized clinical trial
PRF: Platelet-rich fibrin
PRP: Platelet-rich plasma
L-PRF: Leukocyte-PRF (solid clot)
I-PRF: Injectable-PRF (liquid form)
A-PRF: Advanced-PRF (with higher growth factors)
CGF: Concentrated growth factor
DBBM: Deproteinized bovine bone mineral
FDBA: Freeze-dried bone allograft
CI: Confidence interval
SD: Standard deviation
