# Supplementary material for: Tenting screw (tent pole) technique in vertical and horizontal alveolar ridge augmentations: a systematic review and meta-analysis
Source: Maxillofac Plast Reconstr Surg. 2026 Aug 14;48(1):22. doi: 10.1186/s40902-026-00522-y (PMC13476430; doi:10.1186/s40902-026-00522-y)
Supplement: Supplementary file 1 [file 40902_2026_522_MOESM1_ESM.docx]

**Supplementary Table 1.** Extracted data.

| ***Author (Year)/***  ***Study Type*** | ***A) Number of Cases***  ***B) Age***  ***C) Gender (M:F)*** | ***Aim*** | ***Groups*** | ***A) Defect Site***  ***B) Defect Size*** | ***Procedure*** | ***A) Tenting screws***  ***B) Dental Implants*** | ***Evaluation Method(s) (Evaluation Period(s))*** | ***Outcome*** |
| --- | --- | --- | --- | --- | --- | --- | --- | --- |
| Jing Wu et al (2023) /  Case series | A) 20  B) 41.9 years (Average) (Range 27–56 years)  C) NS  *Inclusion criteria:  -Minimum 18 years  -Systemically healthy  -Had at least 4 mm linear alveolar ridge augmentation vertically/horizontally in at least one implant site  *Exclusion criteria:  -Heavy smokers (>10  cigarettes/day)  -Consuming more alcohol | Investigating the clinical effect of GBR combined with modified large-head tenting screws in the reconstruction of a mandibular advanced vertical bone defect | - | A.1.) Posterior mandible  A.2.) Vertical and horizontal  B) Medium to large-sized bone defects  *At least 5 mm threads exposed above the alveoli | 1. Large-head tenting screw-enforced GBR for ridge augmentation (T0) (Months):  1.1. Implanting tent pegs (2 to 3 large-head tenting screws as per the size of bone defects (diameters—6, 8, and 10 mm; lengths—7, 9, 11, 13, and 15 mm) placed vertically (At least 5 mm threads exposed above the alveoli))  1.2. Fully mixed deproteinized anorganic bovine bone mineral (DBBM) alone (Bio-Oss) underneath the large-head tenting screws and on the bone graft  1.3. Placing absorbable Bio-Gide collagen membrane and securing on the buccal aspect of the ridge with 3 nails (At least 2 mini pins)  1.4. Folding the Bio-Gide collagen membrane over the bone defect (T0)  2. Evaluations + staged implant placement (T10) (mean ± SD: 10.05 ± 0.76 months) (Range: 9–12 months) | A.1.) n=2-3 tenting screws as per the size of bone defects A.2.) Large-head  A.3.) Size:  -Diameters: 6, 8, and 10 mm  -Lengths: 7, 9, 11, 13, and 15 mm  B) n= 36  *No information regarding regarding size or brand of implants | 1. CBCT (Linear and volume changes) (Pre (T0) and post-operative (T10))  2. Biopsy (micro-CT and histological analysis) (Bone remodeling after the healing) (T10)  3. Masson’s trichrome stain (Bone remodeling after the healing) (T10)  *Height: Prosthetic-driven virtual implant site, from the alveolar ridge to inferior alveolar nerve  *Width: 2 mm underneath the alveolar crest | 1. No postoperative complications, adverse events or failure during healing of all 36 implants.  2. After 10 months:  2.1. Bone volume/total volume (BV/TV): 27.55 ± 4.59%  2.2. Bone mineral density (BMD): 0.28 ± 0.05 g/cm3  2.3. New bone percentage: 25.95 ± 6.07 (17.4–36.7%)  2.4. Percentage of the remaining scaffold: 30.95 ± 3.79% (23.1–37.0%)  3. New bone gain (vertical and horizontal) around virtual implants in augmented sites with the merging of pre and postoperative CBCT:  3.1. Postoperative bone height (mm): Medium (10.28), Minimum (2.16), Maximum (16.50), Quartiles 1 & 3 (6.08 and 12.32), CI (7.30– 11.28), Mean (9.29), SD (4.24)  3.2. Postoperative bone Width (mm): Medium (8.8), Minimum (4.83), Maximum (18.86), Quartiles 1 & 3 (7.60 and 10.63), CI (7.90–10.92), Mean (9.41), SD (3.23)  3.3. Linear vertical bone gain (mm): Medium (6.56), Minimum (0.05), Maximum (11.77), Quartiles 1 &3 (3.11 and 7.68), CI (4.29–7.23), Mean (5.76), SD (3.14), (Statistically significant difference compared to baseline)  3.4. Linear horizontal bone gain (mm): Medium (3.21), Minimum (0.56), Maximum (8.91), Quartiles 1&3 (2.16 and 4.20), CI (2.22–4.62), Mean (3.42), SD (2.56)  4. Micro-CT analysis of alveolar bone volumetric parameters of bone harvest (T10)  4.1. Tb. N: Medium (3.34), Minimum (2.25), Maximum (4.01), Quartiles 1 &3 (2.82 and 3.82), CI (3.01–3.55), Mean (3.28), SD (0.58)  4.2. BV/TV: Medium (26.84), Minimum (19.50), Maximum (36.73), Quartiles 1 & 3 (21.26 and 31.27), CI (25.36–29.75), Mean (27.55), SD (4.69)  4.3. Tb. Th: Medium (0.078), Minimum (0.070), Maximum (0.104), Quartiles 1 &3 (0.073 and 0.091), CI (0.078–0.089), Mean (0.083), SD (0.012)  4.4. BMD: Medium (0.27), Minimum (0.22), Maximum (0.40), Quartiles 1& 3 (0.23 and 0.31), CI (0.25–0.30), Mean (0.28), SD (0.05)  5. Combination of large-head tenting screws with deproteinized bovine bone and collagen membrane as a promising and exceptional technique in the reconstruction of advanced vertical bone defects in the posterior mandible.  6. Preoperative height:  6.1. Pre-op height: 8.98 mm  6.2. Pre-op width: 9.78 mm |
| Gözde Is ¸ık et al (2020) / Split-Mouth  Randomized Controlled Study | A) Initial= 13  Final=11  B) 50.92 ± 13.01 years (Mean for 11) (Range 20-69)  C) 4:7  *Inclusion criteria:  -Bilateral partial  edentulism with vertical bone loss in the posterior mandible  -Indication for a fixed implant-retained prosthesis  -Insufficient bone height for insertion of short dental implants  -Absence of previously treated periodontal disease  *Exclusion criteria:  - <18 years old  -Radiation therapy  within the last 2 years  -Pregnancy or lactating  -Smoking habit | Evaluating the effect on vertical bone augmentation of the screw tent-pole technique using particulate allograft in  combination with injectable platelet-rich fibrin, and to compare this with autogenous block bone graft | G1) Screw tent-pole technique + particulate allograft with i-PRF + L-PRF membrane (Test side) (Initial=13, Final=11)  G2) Autogenous block bone graft harvested from ramus + L-PRF membrane (Control side) (Initial=13, Final=11) | A.1.) Posterior mandible  A.2.) Vertical  B) NS | 1. Augmentation surgery (T0) (Months)  1.1. Midcrestal incision on either side  G2 side:  1.2.1. Posterior releasing incision over the external oblique ridge to provide access to the donor site  1.2.2. Vertical and horizontal osteotomies  1.2.3. Placing the graft parallel to the posterior border of the horizontal osteotomy  1.2.4. Making screw holes on both the recipient site and the block graft  1.2.5. Fixing the grafts with screws  1.2.6. Covering the periphery of the graft and the underlying gap between the screws and the recipient site with a mixture of particulate allograft (DFDBA)  G1 side:  1.2.1. Making screw holes on the recipient site using a 1.3 mm drill  1.2.2. Fixing screws of 7-mm length on the recipient site and stabilized by a compatible driver  1.2.3. Applying mixture of i-PRF and allograft to the underlying gap between the screws and the recipient site  Both G1 and G2 sides:  1.3. Decortication using a 2-mm diameter round carbide bur and forming six to eight cortical perforations on the recipient beds  1.4. Covering all augmented sites by L-PRF membrane  2. Implant placement surgery (Bio3 Implants) (T6):  2.1. Each group received a type of tapered implant, with triangular thread and a hydrophilic microporous surface  *Additional grafting was required around six implants in G2 | A.1.) Size:  -Thickness: 1.3 mm  -Length: 7-mm  A.2.) Meisinger BTX100 Screw System  B.1.) n=48  G1 (n=24)  G2 (n=24)  B.2.) Bio3 Implants  B.3.) Tapered  B.4.) Triangular thread  B.5.) Hydrophilic microporous surface | 1. CBCT (Pre (T0) and post augmentation surgery (T6))  *Analysis of alveolar bone height in two anatomical planes: The alveolar bone crest and the mandibular canal in the atrophic mandible  *Residual bone height was set at 2 mm from the mandibular canal for both study groups before the augmentation surgery  2. Biopsy (Histological and histomorphometrical analysis) (T6)  3. Implant survival rate (T6 and T12): Absence of pain, erythema or any other signs of infection; absence of exposed implant surface leading to implant mobility and absence of peri-implant radiolucency | 1. Participants and complications:  1.1. 11 of 13 patients completed the study (Reason: Graft failures in 2 surgical sides)  1.2. Complications (In initial number of 13 for each group):  1.2.1. Surgical (Screw fracture): G1 (n=1, 7.7%) and G2 (n=1, 7.7%)  1.2.2. Postoperative:  1.2.2.1. Screw exposure: G1 (n=2, 15.3%)  1.2.2.2. Graft exposure: G2 (n=3, 23%)  1.2.2.3. Graft failure: G2 (n=2, 15.3%)  In 11 patients:  2. Vertical bone gain (mean ± SD): G2 (2.83 ± 0.89 mm) >> G1 (1.72 ± 0.78 mm), (P = .008)  3. Newly formed bone (mean ± SD): G1 (18.08% ± 2.17%) >> G2 (14.26% ± 1.76%), (P < .001)  4. Implant survival rates for G1 & G2 (T12):100%  5. Residual bone height (mean ± SD): G1 (8.14 ± 1.47) > G2 (6.99 ± 1.88), (P = .120)  6. Augmented bone height (mean ± SD): G1 (9.87 ± 1.59 mm) > G2 (9.55 ± 2.38 mm), (P = .59)  7. Histology:  7.1. G1:  7.1.1. Graft material in various portions, with empty osteocyte lacunae  7.1.2. Newly formed bone was analyzed throughout for graft material, and the interface between those portions of biopsy was clearly identified by the presence of increased osteoblastic activity  7.2. G2:  7.2.1. Graft material was not in direct contact with the newly formed bone in some portions  7.2.2. Bone remodeling was evident from the presence of perivascular mesenchymal stem cells, which differentiated into osteoblasts  7.3. G1&2: No evidence of infiltration of acute or chronic inflammatory cells, or of foreign body reactions  8. Residual bone (mean ± SD): G2 (29.75 ± 9.45 %) > G1 (29.73 ± 13.96 %), (P = 1.00)  9. Graft material (mean ± SD): G2 (21% ± 13.12%) >> G1 (11.82% ± 4.28%), (P = .01)  10. Osseointegration period was completed without infection, marginal bone loss, or implant failure |
| Shuai Yuan et al. (2020) / Retrospective cohort | A) 30 patients and sites  B) Mean:  B.1.) G1: 29.5 years  B.2.) G2: 30.4 years  C.1.) G1: 11:4  C.2.) G2: 9:6  *Inclusion criteria:  -Cawood-Howell class IV alveolar ridge defects in anterior maxilla  - >18 years old  -Demanding horizontal bone augmentation at anterior site in the maxilla  *Exclusion criteria:  -Periodontitis  -Pregnancy  -Alcohol and smoke abuse  -Radiation above neck within 5 years | Comparing in-situ bone ring technique with tent-pole technique in order to evaluate their horizontal bone gain, resorption, and postoperative perception | G1) In-situ bone ring (n=15 patients and sites)  G2) Tent-pole (n=15) | A.1.) Anterior maxilla  A.2.) Horizontal  A.3.) n=15 sites for each group  B.1.) Cawood-Howell class IV alveolar ridge defects  B.2.) 1.1. Inserting 1 or 2 titanium screws (Nearly 3 to 6 mm above the alveolar crest)?!  5. Mean horizontal bone gain width (T0+ VS T0):  5.1. 0 mm level: G1 (4.27 ± 0.08 mm) >> G2 (0.07 ± 0.02 mm), (P < .05)  5.2. 3 mm level: G1 (2.44 ± 0.05 mm) >> G2 (2.08 ± 0.06 mm), (P < .05)  5.3. 5 mm level: G1 (2.48 ± 0.03 mm) > G2 (2.47 ± 0.07 mm)  5.4. 7 mm level: G2 (2.97 ± 0.09 mm) > G1 (2.91 ± 0.15 mm)  6. Mean bone resorption (T6 VS T0+):  6.1. 0 mm level: G1 (0.27 ± 0.04 mm) >> G2 (0.03 ± 0.01 mm), (P < .05)  6.2. 3 mm level: G2 (0.84 ± 0.03 mm) >> G1 (0.52 ± 0.06 mm), (P < .05)  6.3. 5 mm level: G2 (0.42 ± 0.05 mm) > G1 (0.38 ± 0.06 mm)  6.4. 7 mm level: G1 (0.05 ± 0.02 mm) > G2 (0.03 ± 0.02 mm) | 1. Bone augmentation (T0) (Months)  G1:  1.1. Bone decortication  1.2. Fixation screw placement in bone ring  1.3. Harvesting bone ring and installing into recipient site  1.4. Covered with xenografts (Organic bovine bone mineral graft) mixed with patient's own blood  1.5. Covered with two-layered absorbable collagen membrane  G2:  1.1. Inserting 1 or 2 titanium screws (Nearly 3 to 6 mm above the alveolar crest)  1.2. Covered with xenografts (Organic bovine bone mineral graft) mixed with patient's own blood  1.3. Covered with two-layered absorbable collagen membrane  Both G1 and G2:  2. Implant placement (T6):  2.1. Removing fixation screws  2.2. Implant placement (Primary stability: ≥ 35 N/cm)  2.3. Implants were submerged for another healing time of 4-6 months  3. Rehabilitation with zirconia crowns (T10-12)  *Source of bone material:  -G1: Autogenous bone  -G2: Xenogenous particulated bone | A.1.) n= 1 or 2 per site  A.2.) Brand: Pro-fix tenting screws  A.3.) Titanium screw  B.1.) Diameter: 3.3 mm  B.2.) Brand: Straumann  B.3.) Primary stability (Torque) : ≥ 35 N/cm | 1. CBCT (Pre augmentation surgery (T0), immediate post augmentation surgery (T0+), post augmentation surgery (T6) and immediate post implantation (T6+))  *For radiographic evaluation, the bone width was measured by dividing the bone defective site into four equal parts in the axial plane as 0 mm, 3 mm, 5 mm, and 7 mm from the ridge crest to determine the amount of horizontal bone mass in each of their own parts  2. Harvesting specimens (H&E and AB (Aniline Blue)) (T6)  3. Postsurgical pain and swelling assessment (VAS  (Visual analog scale)) (D7 postoperative):  -Pain severity: Slight (0-3), moderate (4-6) and severe (7-10)  -Swelling score: 0 (No obvious swelling in both size and shape) to 10 (Distinct swelling)  4. Complication:  -Swelling, hematoma, infection and wound exposure  -Infection symptoms: Redness, swelling, pain, and heat  5. Implant failure (T16-18) | 1. The nasal floor mucosa was exposed within 1 case in G1, but CGF membrane was applied to cover the exposure site which led to favorable clinical outcome with no further complications shown.  2. Complication:  2.1. Swelling:  -The most common  -n=14  -G1 (n=8) > G2 (n=6), (P = .464)  2.2. Hematoma:  -n=9  -G1 (n=5) > G2 (n=4), (P = .599)  2.3. Infection:  -n=3  -G1 (n=2) > G2 (n=1), (P = 1.000)  2.4. Wound exposure:  -n=5  -G1 (n=3) > G2 (n=2), (P = 1.000)  3. VAS ratings (0-10):  3.1. Pain score: G1 (3.10 ± 0.10) > G2 (2.90 ± 0.10), (P = 0.07)  3.2. Swelling score: G1 (2.50 ± 0.15) > G2 (2.30 ± 0.20), (P = 0.18)  4. The whole treatment processes of both groups were successfully performed among all patients. No implant failures were reported after 6 months of loading (T16-18)  5. Mean horizontal bone gain width (T0+ VS T0):  5.1. 0 mm level: G1 (4.27 ± 0.08 mm) >> G2 (0.07 ± 0.02 mm), (P < .05)  5.2. 3 mm level: G1 (2.44 ± 0.05 mm) >> G2 (2.08 ± 0.06 mm), (P < .05)  5.3. 5 mm level: G1 (2.48 ± 0.03 mm) > G2 (2.47 ± 0.07 mm)  5.4. 7 mm level: G2 (2.97 ± 0.09 mm) > G1 (2.91 ± 0.15 mm)  6. Mean bone resorption (T6 VS T0+):  6.1. 0 mm level: G1 (0.27 ± 0.04 mm) >> G2 (0.03 ± 0.01 mm), (P < .05)  6.2. 3 mm level: G2 (0.84 ± 0.03 mm) >> G1 (0.52 ± 0.06 mm), (P < .05)  6.3. 5 mm level: G2 (0.42 ± 0.05 mm) > G1 (0.38 ± 0.06 mm)  6.4. 7 mm level: G1 (0.05 ± 0.02 mm) > G2 (0.03 ± 0.02 mm)  7. Histology (G1):  7.1. H&E: Obvious increase of bone thickness in horizontal  7.2. AB: Outline showed new bone was generated to bridge the native bone and bone ring, which suggested prominent bone fusion at the ring-ridge interface. A majority of xenograft particles was absorbed as to be replaced by the newly formed lamellar bone |
| Nabil Melek L. (2022)/ Randomized prospective controlled clinical  trial | A) 14  B) 52.3 ± 5.9 years (Mean) (Range 41-60)  C) 5:9  *Ratio: 1:1.8  *Inclusion criteria:  -Having about 7 mm of vertical bone height in the posterior mandible above the inferior alveolar canal  -Both genders with age range from 40 to 60 years  *Exclusion criteria:  -Heavy smokers (> 25 cigarettes/day)  -Undergoing radiotherapy or chemotherapy  -Having infection or local lesions in the area of surgery  -Diabetes mellitus  -Bone diseases | Comparing two pre-prosthetic surgical techniques for  augmentation of vertical mandibular ridge defects in view of implant placement | G1) Customized ceramic membranes (n=7)  G2) Tenting screws (n=7) | A.1.) Posterior mandible  A.2.) Vertical  B) 5 mm  (Leaving 5 mm projecting out of the bone) | Bone augmentation (T0) (Weeks):  1. Decortication  G1:  2. Applying milled customized ceramic membranes of zirconia with a vertical height 5 mm above the crest of the ridge  3. Fixing each membrane with a titanium screw  G2:  2. Fixation of 2 tenting screws (Leaving 5 mm projecting out of the bone)  3. Packing particulate bone graft  *The vial used comprises 0.6 ml of nanobone graft formed in the ratio of 76% hydroxylapatite and 24% silica  4. Covering with Hypro-Sorb® F collagen membrane (A double-layered structure with one smooth side toward the soft tissue and another rough side toward the bone)  *Collagen membrane was inserted on the lingual surface of the defect then the membrane was rolled, adapted over the screws/graft and fixed on the buccal side with bone tacks (Fixed by bone tacks (modified sausage technique)) | A.1.) Size:  2.0 mm  -Length: 9 mm  A.2.) Self-tapping  A.3.) Titanium tenting screws  A.4.) n=2 per defect  B) NA | 1. CBCT (Increase in vertical ridge height) (Pre (T0) and postoperative (T24))  *Distance from the crestal bone to the inferior alveolar nerve was calculated to be compared with the baseline preoperative height of bone  2. Complications (T1 and T2):  2.1. Infection (Swelling, redness, hotness, pus discharge, and pain)  2.2. Wound healing disturbance (Wound dehiscence and hardware exposure) | 1. Clinical follow up:  1.1. Uneventful healing:  G2 (n=5) > G1 (n=4)  1.2. Wound dehiscence:  -G1 (T2) (n=3 (Of 7), 42.9%)  *Re-suturing for 1 and regular antiseptic mouth rinse for 2  -G2 (T1) (n=2 (Of 7), 28.6%)  *Re-suturing for both (One of them has shown screw head re-exposure through the wound and conservative treatment was implemented)  2. Mean bone height from the crest of the ridge to the inferior alveolar nerve:  -Preoperative (T0): 7.146 ± 0.24 mm  -Postoperative (T24): 10.266 ± 0.97 mm  3. Mean percentage of change of bone height:  G1 (52.8204 ± 8.76720%) >> G2 (34.4978 ± 9.17603%), (p = 0.002) |
| Janina Golob Deeb et al (2020) / Retrospective chart review | A) 43  B) 60.96 ± 11.73 years (Mean) (Range 31-79)  C) (n=12, 28%): (n=31, 72%)  *Exclusion criteria:  -Simultaneous horizontal ridge augmentation and implant placement  -Use of graft materials other than particulate bone grafts  -Use of non-resorbable membranes  -Augmentations involving techniques other than tenting screws  -Patients without a preoperative and  postoperative CBCT scan  -Edentulous | Investigating if the addition of biologic agents to a particulate bone graft enhances horizontal ridge augmentation outcomes  in terms of bone dimensions, bone density, and successful implant placement | G1) Particulate bone graft and adjunctive biologic agents (rhPDGF-BB (n=7) or PRP (n=14)) (n=21 sites, 40%)  G2) Particulate bone graft (n=31 sites, 60%) | A.1.) Jaw (Sites):  -Maxilla (n=17, 33%)  -Mandible (n=35, 67%)  A.2.) Position (Sites)  -Anterior (n=10, 19%)  -Posterior (n=42, 81%)  A.3.) Horizontal  A.4.1.) n=52  A.4.2.) M:F (Sites):  (n=13, 25%):(n=39, 75%)  B) The number of implant sites ranged 1 - 4 with an average of 1.2 sites per patient  3 mm of screws supported the particulate bone graft) | 1. Ridge augmentation procedure (T0) (Months):  1.1. Applying a mixture of various proportions of particulate mineralized FDBA and bovine-derived xenograft (Biologic agents (rhPDGF-BB or PRP) were mixed into the particulate bone graft in 21 cases in G1)  1.2. Supporting bone graft by tenting screws (3 mm served for intraosseous retention and 3 mm to support the particulate bone graft)  1.3. Covering with a resorbable collagen membrane or an acellular dermal matrix for GBR  2. Implant placement | A) Length: 6mm  B) NS | 1. CBCT (Gains in alveolar ridge bone width) (Pre (T0) and postoperative (T7.3 (On average)))  2. CareStream CBCT scan software (Horizontal measurements and density)  *The horizontal dimensions of the alveolar ridge in the desired implant sites were measured before and after augmentation of 3 mm apical to the alveolar crest at three set points 3 mm apart. The distal surface of the nearest adjacent tooth served as a reproducible reference point for the measurements. | 1. Baseline measures:  1.1. Baseline bone level (width) (mm):  -Overall (5.39 ± 1.32)  -G2 (5.62 ± 1.48) > G1 (5.06 ± 0.99), (p = .1411)  1.2. Follow-up bone level (width) (mm):  -Overall (8.88 ± 2.03)  -G2 (9.03 ± 2.00) > G1 (8.64 ± 2.09), (p = .5094)  1.3. Average augmentation (mm):  -Overall (3.59 ± 1.84), (p < .0001)  -G2 (3.60 ± 1.79) > G1 (3.58 ± 1.96)  1.4. Postop bone graft density:  -Overall (1,025.15 ± 515.94)  -G1 (1,130.81 ± 384.80) > G2 (953.58 ± 583.70)  1.5. Time between grafting and postop CBCT (months):  -Overall (7.33 ± 4.19)  -G2 (8.00 ± 5.03) > G1 (6.33 ± 2.27), (P = .1128)  2. G1 was similar to G2 in terms of age (p = .1388), gender (p = .3325) and jaw (p = .2378)  3. Frequency and type of biologics used:  3.1. Anterior: G1 (PRP (n=5, 50.0%), rhPDGF-BB (n=4, 40%)) > G2 (n=1, 10.0%)  3.2. Posterior: G2 (n=30, 71.4%) > G1 (PRP (n=9, 21.4%), rhPDGF-BB (n=3, 7.1%))  3.3. G1: Anterior (90.0%) >> Posterior (28.5%), (p = .0006)  3.4. G1: Selection of rhPDGF versus PRP was not associated with implant site (rhPDGF-BB: anterior (57%) > Posterior (43%), (p = .3972))  4. Augmented sites were able to receive implants in 98% of the cases (n = 51). 1 patient did not return for implant placement and the outcome is unknown  *One patient did not return for implant placement and the outcome is unknown  5. Initial association between bone level, bone density, and use of biologics (Mean, 95% CI) (Mean adjusted for time since bone graft and baseline bone level (For postoperative bone level)):  5.1. Postoperative bone level (width):  -G2 (9.09 mm, 8.42 to 9.76) > G1 (8.73 mm, 7.93 to 9.54)  -Difference (−0.35, −0.72 to 1.43), (P = .5094)  5.2. Bone graft density:  -G1 (1,143.87, 757.08 to 1,132.39) > G2 (944.73, 915.01 to 1,372.74)  -Difference (199.14, −99.62 to 497.90), (P = .1866)  6. Average initial width of the alveolar ridge:  -Overall: 5.39 mm  -G2 (5.62) > G1 (5.06), (p = .1411)  7. Horizontal bone gain:  7.1. Average: Overall (3.6 mm, SD = 1.84), A statistically significant gain (p value < .0001; 95% CI: 3.04–4.07)  7.2. G2 (3.60 mm, SD = 1.79) > G1 (3.58 mm, SD = 1.96)  8. Average baseline alveolar ridge width, bone gain, and postoperative alveolar ridge width (mm):  G2 (Baseline (5.62 mm), Gain (3.60 mm), Post (9.03 mm)) > G1 (Baseline (5.06 mm), Gain (3.58 mm), Post (8.64 mm)), (p = .5094)  9. Adjusted model for association between bone level and biologics (Estimated mean postoperative bone level, 95% CI):  9.1. Baseline bone level (width) (1 mm increase): (0.45, 0.05 to 0.84), (p = .0294)  9.2. Biologics: G1 (8.57, 7.79 to 9.35) > G2 (8.29, 7.37 to 9.20), (p = .6196)  9.3. Healing time (Months from surgery (1 month increase)): (−0.10, −0.22 to 0.02), (p = .0961)  9.4. Location: Posterior (9.31, 8.73 to 9.90) > Anterior (7.54, 6.33 to 8.76), (p = .0172)  9.5. For each 1 mm of increase in baseline bone width, there was an associated postoperative gain of 0.45 mm  9.6. Factors that were not statistically significant were removed with backwards elimination in the following order: Jaw (p = .6117), gender (p = .5324) and age (p = .3829)  10. Adjusted model for association between bone graft density and use of biologics  (Estimated bone density, 95% CI):  10.1. Biologics:  -G1 (1,106.19, 877.19 to 1,335.18)) > G2 (839.64, 617.68 to 1,061.60), (p = .0841)  -Average difference (266.55), 95% CI (−37.27, 570.38)  -The use of biologics was not associated with a significant difference in bone density (p = .1866)  10.2. Healing time (Months from surgery (1 month increase)): (27.47, −11.06 to 66.00), (p = .1582)  10.3. Gender: Female (1,128.65, 953.79 to 1,303.52) > Male (817.17), 513.30 to 1,121.04), (p = .0944)  10.4. Mean bone density: PRP (1,184.36) > rhPDGF-BB (1,023.71)  10.5. Factors that were not statistically significant were removed with backward elimination in the following order: Location in the mouth (p = .5678), jaw (p = .6165), and age (p = .5439) |
| Janina Golob Deeb et al (2019) / Retrospective chart review | A.1.) Total (n=73)  A.2.) Radiographic analysis (n=53)  B) 60.59 ± 11.36 years (Mean)  C) (n=17, 23%): (n=56, 77%)  *Exclusion criteria:  -Patients treated in Graduate Periodontics with simultaneous horizontal ridge augmentation and implant placement  -Grafting materials other than particulate bone graft  -Non-resorbable membranes  -Without tenting screws | Investigating if the prescription of oral postoperative steroids has an effect on clinical outcomes of horizontal ridge augmentation including implant placement and characteristics of the grafted bone | G1) Steroids (n=48, 66%)  G2) No steroids (n=25, 34%) | A) Horizontal  B) NS  3mm of screw outside of bone) | 1. Ridge augmentation procedure (T0) (Months):  1.1. Placing various mixtures of mineralized FDBA, particulate bovine-derived hydroxyapatite xenograft and allograft putty materials (Exactech Optecure +CCC)  1.2. Supporting bone graft by tent screws (Secured to depths of 3 mms into the recipient site bone and 3mm outside of bone)  1.3. Placing a variety of resorbable collagen membranes or acellular dermal matrix for GBR (Alloderm GBR), or a bovine collagen sponge with synthetic particulate graft  1.4. Securing membranes with tacks, sutures or tucked  2. Implant placement | A.1.) Length: 6 mm  A.2.) Brand: Salvin Dental Specialties  B) Implant placement: -Yes: n=67, 93%  -No: n=3, 4%  -In Progress: n=2, 3% | 1. CBCT (Grafted bone volumetric changes and density) (Pre (T0) and postop (T1-24 before implant placement)  *Horizontal measurements were made 3 mm apical to the alveolar crest in the area of future implant placement to account for any potential discrepancies, and reference points on adjacent dentition served as fiducial landmarks | 1. Radiographic analysis (n=53): 1.1. Baseline bone level: 5.42 ± 1.33  1.2. Follow-up bone level: 8.92 ± 2.03  1.3. Average growth: 3.56 ± 1.82 mm, (P < 0.05)  1.4. Months between (Growth): 10.58 ± 5.09  1.5. Follow-up density: 1033.04 ± 514.17  1.6. Time between (Density): 7.32 ± 4.15  2. Post-operative clinical measures (n=73)  2.1. Graft exposure (Yes vs No: p = 0.7070):  -Yes (n=8, 11%): G1 (n=6, 13%) > G2 (n=2, 8%), (p >0.05)  -No: G1 (n=42, 88%) > G2 (n=23, 92%)  2.2. Postoperative visits (Medium, Range): G1 (2, 1-6), G2 (2, 1-3), (p = 0.1494)  * G1 (2.3) > G2 (1.8)  2.3. Courses of antibiotics (Median, Range): G1 (1, 1-2), G2 (1, 0-3), (p = 0.9291)  3. Almost all of the cases included have seen implants successfully placed (93%)  4. G1 was not significantly different from G2 in terms of age (p >0.05) or gender (p >0.05)  5. The use of steroids was not associated with a significant increase in bone level after graft (p > 0.05) |
| Asmaa M. Abd El Fattah et al (2021) / Clinical and radiographic study (Case series) | A) 12  B) 52.08 ± 5.84 (Mean) (Range 43-59)  C) 4:8  *Ratio: 1:2  *Inclusion criteria:  -40 and 60 years old  -Missing lower tooth or teeth  -Having a related large vertical bony defect, 7mm residual bone height (from the crest of the ridge to the inferior alveolar nerve)  -Requiring about 5 mm rise in the vertical height of the mandibular alveolar ridge for future prosthetic rehabilitation  *Exclusion criteria:  -Infection or local lesions  -Parafunctional habits  -Current chemotherapy or radiotherapy  -Heavy smoking  -Alcohol or drug abuse  -Medically compromised patients | Evaluating clinically and radiographically the efficiency of "Tent- Pole" grafting using titanium mini-screws with GBR for augmentation of mandibular ridge vertical defects | - | A.1.) Posterior mandible  A.2.) Vertical defect or saucer shaped depression  B.1.) Requiring about 5 mm rise  B.2.) Having a related large vertical bony defect, 7mm residual bone height (from the crest of the ridge to the inferior alveolar nerve)  (Exposing 3-5mm of the screw length)  2. Bone height (Mean ± SD):  2.1.  -T0: 7.18 ± 0.49 (Mean), (Range 6.3 – 7.0 mm)  -T0+: 10.40 ± 1.68 (Mean), (Range 7.0 – 12.5 mm)  -T24: 9.23 ± 1.25 (Mean), (Range 7.0 – 11.1 mm)  -T0+ >> T0, (p < 0.001)  -T24 >> T0, (p < 0.001)  -T0+ >> T24, (p < 0.001) | 1. Guided bone regeneration (T0) (Weeks):  1.1. Screw fixation (n=2) over the alveolar ridge (Exposing 3-5mm of the screw length)  1.2. Decortication  1.3. Fixing collagen membrane (Hypro-Sorb® F, rigid, absorbable, bovine, sterile atelo-collagen, double-layered) lingual to the defect  1.4. Synthetic bone graft material (NanoBone®, alloplastic  material with no animal or human elements) completely covering the screw heads  *Overcorrecting with particulate material  1.5. Placing PRF membrane on top of the grafting material  1.6. Rolling collagen membrane and fixing on the buccal side with bone tacks 3.5 mm | A.1.) Osteosynthesis mini-  Screws  A.2.) Self-tapping titanium mini screws  A.3.) Size:  -Width: 2.0 mm  -Length: 7.9 mm  A.4.) n=2 per site  A.5.) Brand: Antonhib  B) NS | 1. CBCT (Bone height and density) (Pre (T0), immediate post (T0+) and postoperative (T24))  *From the alveolar crest till the inferior alveolar nerve  2. Clinical evaluation (D1, T1 and T2):  -Pain (VAS (Visual analogue scale)) (0-10): No (0), Worst (10)  -Edema (Pitting capability):  +1 (trace) insignificant indentation rapid returns to normal  +2 (mild) the indentation returns to normal in few seconds  +3 (moderate) 6 mm indentation rebounds in 10-20 seconds  +4 (sever) 8 mm indentation rebounds in more than 30 seconds  -Wound healing | 1. Clinical evaluation:  1.1. Pain value:  -D1 (All patients): 8.00 ±1.60 (Mean), (Range 6.0 - 10.0)  -T1 (All patients): 3.50±1.51 (Mean), (Range 2.0 - 6.0)  -T2 (All patients): Mild, 1.17±0.39 (Mean), (Range 1.0 – 2.0)  1.2. Edema value:  -D1 (All patients): 3.0 – 4.0 (Range)  -T1 (All patients): 1.0 – 2.0 (Range)  -T2: 1.0  1.3. Wound healing complications:  T2: Incomplete wound healing (Wound dehiscence) (n=4):  -Exposed screw heads (Partial dehiscence) (n=2, 16.67%)  -Graft loss (Complete dehiscence) (n=2, 16.67%)  *Complete wound healing in 8 cases (66.67%)  1.4. Infection: None  2. Bone height (Mean ± SD):  2.1.  -T0: 7.18 ± 0.49 (Mean), (Range 6.3 – 7.0 mm)  -T0+: 10.40 ± 1.68 (Mean), (Range 7.0 – 12.5 mm)  -T24: 9.23 ± 1.25 (Mean), (Range 7.0 – 11.1 mm)  -T0+ >> T0, (p < 0.001)  -T24 >> T0, (p < 0.001)  -T0+ >> T24, (p < 0.001)  3. Gray density value (Mean ± SD):  -T24 (353.25±53.75) >> T0+ (270±68.92), (p = 0.00)  -Improvement rate: 30,67% |
| G. A. Guillen et al. (2020) / Single-centre, prospective, randomized, split-mouth (Clinical trial) | A) 10  B) 40.7 ± 13.3 years (Mean) (Range 16 – 58)  C) 6:4  *Inclusion criteria:  -Simultaneous loss of the 4 upper incisors only  -Bone height of 10 mm or more  -Insufficient alveolar bone width (Range 2 - 3.5 mm)  -Requiring a bone graft for implant placement  *Exclusion criteria:  -Having health conditions that precluded surgical interventions  -Bone defects that were not exclusively horizontal  -Absent canines as well as less than four upper incisors loss  -Alveolar width less than 2 mm or more than 3.5 mm | Evaluating the use of the screw tent-pole technique in the repair of width defects of the premaxilla by comparing standard tenting screws and wide-head tenting screws | G1) Standard screw (n=3 per site)  G2) Wide-head screw (n=3 per site) | A.1.) Anterior maxilla  A.2.) Horizontal  A.3.) n=20 sites  B.1.) Loss of 4 upper incisors  B.2.) Insufficient alveolar bone width (Range 2 - 3.5 mm)  4 mm protruding above the maxillary surface | 1. Augmentation (T0) (Months):  1.1. Perforating graft recipient bed  1.2. Placing 3 standard tenting screws on one half + 3 wide-head tenting screws on the other half  *All screws were positioned in a triangular arrangement (2.0 mm x 10 mm) with the base of the triangle towards the occlusal region and with 4 mm protruding above the maxillary surface  1.3. Positioning protein-free bovine bone substitute (Lumina Porous, particle size 1000–2000 μm) (Chemically treated and agglutinated with i-PRF) between the tenting screw regions (0.5 g on each side)  1.4. Covering by 4 PRF membranes  2. Screw removal + implant placement (T9): Using a 2-mm diameter trephine to collect material for histological analysis from the site where the implants were to be installed  3. Prosthetic rehabilitation (T15) | A.1.) Number:  -n=6 per site  -G1 (n=3 per site)  -G2 (n=3 per site)  A.2.) Head diameter:  -G1: 2.28 ± 0.03 mm  -G2: 3.84 ± 0.02 mm  A.3.) Head area:  -G1: 4.08 mm2  -G2: 11.58 mm2  A.4.) Brand G1 & G2: Neodent  B) NS | 1. CBCT (Bone width) (Pre (T0) and postoperative (T9))  2. H&E (Vital bone tissue, remaining bone substitute particles, and medullary space/connective tissue) (T9)  3. Clinical evaluation (T0 and T9):  3.1. Alveolar surface (Based on observation of its buccal surface):  -Flat  -Concave  3.2. Bone width  *Clinical and CBCT bone width:  -Measurements on the alveolar ridge and at 5 mm and 10 mm from the ridge using a calibrated Backhaus-shaped caliper  -The screw placed at the vertex of the pyramidal distribution was the one used as a reference for all measurements and was positioned at 10 mm from the alveolar ridge | 1. No patient loss during the follow-up period and no clinical or CBCT analysis data missing  2. Excluding 3 of the 20 histological samples due to damage during surgical collection or histological processing  3. Complications:  3.1. n=2 patients  3.2. Week 1 (n=1): Wound dehiscence + membrane exposure (Resolved with uneventful secondary intention healing on a PRF fibrin-like surface)  3.3. T7 (G2) (n=1): Partial screw exposure  3.4. Neither of these complications resulted in failure of the grafting procedure  4. Clinical measurements (mm):  4.1. Alveolar ridge:  4.1.1. Initial bone width: G1 (2.25 ± 0.71) > G2 (2.00 ± 0.97)  4.1.2. Final bone width: G2 (3.50 ± 1.50) > G1 (3.30 ± 1.16)  4.1.3. Ridge width gain: G2 (1.50) > G1 (1.05), (P = 0.97)  4.2. 5 mm from the ridge:  4.2.1. Initial bone width: G1 (4.05 ± 0.95) > G2 (3.70 ± 1.16)  4.2.2. Final bone width: G2 (6.80 ± 1.87) > G1 (6.50 ± 1.78)  4.2.3. Ridge width gain: G2 (3.10) > G1 (2.45), (P = 0.79)  4.3. 10 mm from the ridge:  4.3.1. Initial bone width: G2 (6.15 ± 1.27) > G1 (6.10 ± 1.17)  4.3.2. Final bone width: G2 (9.60 ± 2.01) > G1 (8.80 ± 1.54)  4.3.3. Ridge width gain: G2 (3.45) > G1 (2.70), (P = 0.44)  5. CBCT measurement (mm):  5.1. Alveolar ridge:  5.1.1. Initial bone width: G2 (2.55 ± 1.12) > G1 (2.39 ± 0.45)  5.1.2. Final bone width: G2 (3.46 ± 1.24) > G1 (3.13 ± 0.68)  5.1.3. Ridge width gain: G2 (0.91) > G1 (0.74), (P = 0.68)  5.2. 5 mm from the ridge:  5.2.1. Initial bone width: G2 (5.26 ± 1.64) > G1 (5.26 ± 1.00)  5.2.2. Final bone width: G2 (9.31 ± 2.04) > G1 (9.14 ± 1.24)  5.2.3. Ridge width gain: G2 (4.05) > G1 (3.88), (P = 0.96)  5.3. 10 mm from the ridge:  5.3.1. Initial bone width: G1 (9.04 ± 2.13) > G2 (9.03 ± 3.07)  5.3.2. Final bone width: G1 (13.76 ± 3.07) > G2 (13.40 ± 3.11)  5.3.3. Ridge width gain: G1 (4.72) > G2 (4.37), (P = 0.95)  6. Histomorphometric evaluation (Percentage area values):  6.1. Vital bone: G2 (32.64 ± 20.28) > G1 (30.99 ± 26.43), (P = 0.95)  6.2. Bone graft residue: G1 (11.32 ± 9.68) > G2 (9.73 ± 5.82), (P = 0.71)  6.3. Connective tissue: G1 (57.66 ± 21.85) > G2 (57.61 ± 20.15), (P = 0.85)  7. Ridge:  7.1. Concave (n=8 patients)  7.2. Flat (n=2)  7.3. Patients with a flat ridge showed poorer histomorphometric outcomes following the screw tent-pole technique when compared to patients with a concave-shaped alveolar ridge  8. Etiology of tooth loss: Extraction due to tooth decay (n=5) and trauma injury (n=5) |
| Daniel farias et al. (2021) / Prospective (Case series) | A) 14  B) 56.8 ± 7.8 years (Mean) (Range 45-73)  C) 4:10  *Inclusion criteria:  -American society of anesthesiologists Class I or II men or women undergoing dental implant treatment in posterior mandible  *Exclusion criteria:  -Osteoporosis  -Bone metabolism related drug users  -Smokers  -Active periodontal disease  -History of cancer, chemotherapy or radiation therapy | Analyzing:  1. Horizontal bone augmentation in the posterior edentulous mandible using the tenting screw technique with allograft, and leukocyte and L-PRF  2. Subsequent primary and secondary stability of the implants | - | A.1.) Posterior mandible  A.2.) Horizontal  A.3.) Implant sites:  -Canine level (n=1)  -First premolar (n=1)  -Second premolar (n=10)  -First molar (n=11)  -Second molar (n=4)  B) Tooth loss in premolar or molar areas with a minimum height of 9 mm from the mandibular canal and a maximum bone width of 3 mm  Inserting 2-4 screws (1.5 mm) 45 degrees from the occlusal plane (Maintaining 4 mm of extraosseous space) | 1. Bone augmentation surgery (T0) (Months):  1.1. Superficial milling of the cortical bone + monocortical perforation on the buccal area  1.2. Inserting 2-4 screws (1.5 mm) 45 degrees from the occlusal plane (Maintaining 4 mm of extraosseous space)  1.3. Filling the defect with L-PRF block (A mixtures of segmented PRF membrane and bone substitute material)  *The entire 4 mm space under the screws made intimate contact with the cortical bone  1.4. Covering with resorbable collagen membrane (Biomed Extend)  1.5. Installing L-PRF membrane over the resorbable membrane  2. Implant surgery (T4):  2.1. Removing screws  2.2. Drill with a 3.75 mm diameter and 8-10 mm height  3. Prosthetic rehabilitation (Fixed) (T8): Loading implants with a single screw or splint prosthetic system | A.1.) Width: 1.5 mm  A.2.) Brand: Artfix Implants  A.3.) n=2-4 screws per site  B.1.) n=27  B.2.) Drill with a 3.75 mm diameter and 8-10 mm height  B.3.) Brand: Zimmer tapered Screw-Vent MTX surface  B.4.) Insertion torque:  55.6 ± 10.17 Ncm (Range 35 – 70 Ncm)  B.5.) ISQ:  -T4: 69.34 ± 7.12 (Range 55.5 - 81)  -T8 (Prior to loading): 75.9 ± 3.29 (Range 69 – 80)  -ISQ T8 > ISQ T4, (p > 0.05) | 1. CBCT (Horizontal bone gain) (Pre (T0) and postoperative (T4, T8 and T12)  *Measurements were performed from the coronal area using the landmark of teeth  2. Insertion torque and ISQ (Implant stability quotient) (Implant primary stability) | 1. Horizontal bone width:  1.1. Preoperative (T0):  -Range: 1.2 – 4 mm  -Average ± SD: 2.95 ± 0.59 mm  1.2. Postoperative (T4):  -Range: 3.79 – 10.05 mm  -Average ± SD: 7.15 ± 1.57 mm  1.3. Difference (Gain):  -Average ± SD: 4.2 ± 1.26 mm  1.4. Bone width T4 >> Bone width T0, (p = 0.03)  2. Bone gain (Volume):  -Range: 1.12 – 167.75 mm2  -Average ± SD: 46.9 ± 32.79 mm2  3. Complications:  3.1. No intraoperative complications were associated with the surgical technique.  3.2. No suture dehiscence or membrane exposure  3.3. Head exposure of screws:  -4 sites (T2) (Thin periodontal biotype)  3.4. Paresthesia: 1 patient (Improving at 2 weeks post surgery)  4.2.2.  5. All implants were maintained in function at 3-months and 6-months follow-ups |
| Mehdi Keddar et al (2024) / Prospective interventional clinical study | A) 42  B) 48.2 years (Average) (Range 23-65)  C) 17:25  *Inclusion criteria:  -Insufficient alveolar bone width for dental implant  Placement  -Defects up to Cawood and Howell class IV (knife edge of adequate height but insufficient width)  *Exclusion criteria:  -Age < 18 years  -Current pregnancy  -Current smoking status (>10 cigarettes/day)  -Undergoing treatment that alters bone quality (e.g. bisphosphonates, denosu-  mab)  -Uncontrolled systemic bone disease (e.g. hyperparathyroidism,  chronic renal failure)  -Having received radiotherapy or chemo-  therapy within 12 months prior to treatment | Radiologically evaluation of the horizontal bone gain obtained using a GBR procedure combining a particulate allograft, PRF, resorbable collagen membrane, and screw tents | - | A.1.)  -Maxilla (n=29, 46%)  -Mandible (n=34, 54%)  -Embedded sectors (n= 27, 42.8%)  -Terminal sectors (n=36, 57.2%)  A.2.) Horizontal  A.3.) n=63 defects  B) NS  3-4 mm between the bone level and the head of the predetermined screws) | 1. GBR (n=63 bone defects) (T0) (Weeks):  1.1. Drilling bone  1.2. Placing screw (3-4 mm between the bone level and the head of the predetermined screws)  1.3. Covering by buffy coats and a mixture of particulate allograft DFDBA (Particle size of 300−500 μm 2/3 and 500−1000 μm 1/3) until head of screws  1.4. Covering by resorbable collagen membranes (Geistlich Bio-Gide)  1.5. Covering by autologous fibrin membrane (A-PRF)  2. Prosthesis rehabilitation (T2-4)  3. Implant placement + mini-screw removal | A.1.) Stryker titanium osteosynthesis mini screws  A.2.) Size:  -Diameter: 1.2 mm  -Length: 5−12 mm  B) NS | 1. CBCT (Bone volume) (Pre (T0) and postoperative (T28-36), (9.1 ± 2.0 Months))  *First, the grafted area and the inferior alveolar nerve canal or another landmark anatomical structure that is clearly identifiable and stable over time were identified on the pre-and post-operative images. Ridge thickness was measured using the pre-operative scanner. Next, the distance between the reference structure and the measured ridge was measured. On the post-operative scanner, a line was traced whose length corresponded to the distance measured on the post-operative scanner. The grafted ridge thickness was measured at this level  2. Control examination (Wound healing and complication) (T1, T2, T4 and T16-20)  3. Preoperative examination: Any hypovitaminosis D (25-OH-vitamin D < 30 ng/mL) was corrected pre-operatively with the aim of improving bone healing,  which is crucial for long-term implant | 1. Bone increase after 9.1 ± 2.0 months:  1.1. Mean ± SD: 3.2 ± 0.9 mm, (p < 0.001)  1.2. Range: 1.1 – 5.6 mm  *Regardless of bone defect location  2. Influence of qualitative variables on mean bone gain:  2.1. Age: 50 years and older (3.3 ± 1.0 mm) > Younger patients (3.0 ± 0.7 mm), (p = 0.139)  2.2. Sex: Female (3.2 ± 0.8 mm) > Male (3.1 ± 0.9 mm), (p = 0.892)  2.3. Type of edentulism: Terminal (3.2 ± 0.9 mm) > Embedded (3.0 ± 0.8 mm), (p = 0.478)  3. No complications, such as dehiscence, membrane exposure, or infection |
| George R. Deeb et al (2017) / Retrospective cohort | A) 35  B) NS  C) NS  *Inclusion criteria:  -18 years or older  -Undergone horizontal ridge augmentation with tent-screw pole technique  *Exclusion criteria:  -A component of vertical ridge augmentation also was performed  -Horizontal augmentation was combined with simultaneous implant placement, or adjunctive methods such as PRP or BMP had been used | Evaluating efficacy of the tent-screw pole technique for horizontal ridge augmentation and to compare the results to the tunnel technique and open ridge augmentation (From previous studies) | G1) Tent-screw pole (n=35)  G2) Tunnel (n=20) (Data from previous studies)  G3) Open PTFE (Titanium-reinforced) (n=31) (Data from previous studies) | A) Horizontal  B) NS  Approximately 3 - 6 mm of the screw exposed above the alveolus) | 1. Ridge augmentation (T0) (Weeks):  G1:  1.1. Placing 2 – 4 titanium screws in the alveolar ridge deficiency area (Approximately 3 - 6 mm of the screw exposed above the alveolus)  1.2. Placing particulate bone graft (1:1 mixture of mineralized FDBA (Puros) and particulate bovine-  derived hydroxyapatite( into the site until only the surface of the screws was visible  1.3. Placing resorbable collagen membrane over the screws and grafted site without retention with screws or tacks  G2 (From previous studies):  1.1. Creating subperiosteal tunnel  1.2. Injection of graft material (1:1 mix of mineralized FDBA (Puros) and particulate bovine-derived hydroxyapatite)  G3 (From previous studies):  1.1. Securing titanium-reinforced PTFE (Cytoplast, Osteogenics) membrane on the palatal aspect of the maxilla or lingual aspect of the mandible first with at least 1 titanium screw  1.2. Placing bone graft (1:1 mix of mineralized FDBA (Puros) and particulate bovine-derived hydroxyapatite  1.3. Membrane folded over the graft and secured on the buccal aspect of the ridge with 2 titanium screws  2. Implant placement + tenting screw removal | A.1.) Number:  -G1 (n=2-4 per defect)  -G3 (n=3 minimum per case)  A.2.) Pro-fix™ osteogenics titanium tenting screws  A.3.) Width: 1.5 mm  A.4.) Self-drilling tip, polished neck, and broad head  B) NS | 1. CBCT (Ridge topography) (Pre (T0) and postoperative (T24))  2. Follow-up (T1, T4 and T24) | 1. Patients with dehiscence in the absence of infection were managed with chlorhexidine and wound cleansing  2. Sufficient alveolar ridge width for successful implant placement (T24):  2.1. Implant placement rate:  -G1 (n=33 of 35, 97%) >> G2 (n=18 of 21, 86%) >> G3 (n=22 of 31, 71%), (p = 0.0536)  -G1 >> G3 (Adjusted p = 0.0593)  -G2 > G3 (Adjusted p = 0.4583)  -G1 > G2 (Adjusted p = 0.3347)  3. Membrane Exposed/Wound Dehiscence:  -G3 (n=16 of 31, 52%) >> G2 (n=4 of 21, 19%) > G1 (n=4 of 35, 11%), (p = 0.0033)  -G3 >> G2, (Adjusted p = 0.0447)  -G3 >> G1, (Adjusted p = 0.0027)  -G2 > G1, (Adjusted p = 0.7458)  4. Graft loss:  -G3 (n=12 of 31, 39%) > G2 (n=4 of 21, 19%) > G1 (n=3 of 35, 9%), (p = 0.0256)  -G3 >> G1, (Adjusted p = 0.0275)  -G3 > G2, (Adjusted p = 0.3205)  -G2 > G1, (Adjusted p = 0.5033) |
| Gregory R. Caldwell et al (2015) / Nonmasked randomized prospective clinical trial | A.1.) Initial: 27  Final: 24  B) 50.2 ± 12.6 years (Mean) (Range 25-73)  C) 4:20  *Inclusion criteria:  -Systemically healthy  -Partially edentulous ridge  -Inadequate buccolingual dimension for standard diameter implant placement  *Exclusion criteria:  -Heavy smoking ( > 10 cigarettes/day)  -Active periodontal disease  -Pregnancy  -Infectious disease  -History of bone-altering medications that could interfere with the treatment  -Uncontrolled systemic disease | Evaluating the clinical benefits, or lack thereof, of using an allograft alone or in combination (1:1) with particulated autogenous bone for horizontal ridge augmentation and subsequent implant placement | G1) Autogenous + FDBA (n final excluding GBR failures =11 sites)  G2) FDBA (n final excluding GBR failures=12) | A.1) Horizontal  A.2.) Maxilla and mandible  A.3.) Anterior and posterior  B) n=33 implants in 23 sites of 21 patients  2. Average ridge width of tenting screw technique with GBR membrane (T6):  2.1. Including the 3 sites with postop infections (n=26 sites):  2.1.1. Average gain: 2.92 ± 1.08 mm (Range: 0.50 – 5.00 mm)  2.1.2. Initial: 3.63 ± 1.40 mm (Range: 1.0 – 6.5 mm)  2.1.3. Attempted gain: 3.77 ± 0.57 mm (Range: 3.0 – 5.0 mm)  2.1.4. At reentry: 6.58 ± 1.34 mm (Range: 3.0 – 9.0 mm)  2.1.5. Graft resorption: 0.85 ± 1.06 mm (Range: 0.0 – 3.5 mm)  2.2. Excluding the 3 sites with postop infections (n=23 sites):  2.2.1. Average gain: 3.22 ± 0.74 mm (Range: 2.0 – 5.0 mm)  2.2.2. Initial: 3.50 ± 1.31 mm (Range: 1.0 – 6.0 mm)  2.2.3. Attempted gain: 3.76 ± 0.60 mm (Range 3.0 – 5.0 mm)  2.2.4. At reentry: 6.72 ± 1.18 mm (Range: 4.0 – 9.0 mm)  2.2.5. Graft resorption: 0.54 ± 0.66 mm (13.89% ± 16.0%) (Range: 0.0 – 2.0 mm)  4. Clinical measurements excluding GBR failures (n total=23) (G1 (n=11), G2 (n=12)):  4.1. Age (y):  4.1.1. Mean ± SD:  -Overall: 48.95 ± 12.59  -G2 (49.50 ± 11.80) > G1 (47.45 ± 14.51), (Mean: -2.05), (p = 0.71)  4.2. Healing time (wk):  4.2.1. Mean ± SD:  -Overall: 24.34 ± 2.91  -G1 (24.65 ± 3.39) > G2 (23.85 ± 2.77), (Mean: 0.80), (p = 0.54)  4.3. Initial ridge width (mm):  4.3.1. Mean ± SD:  -Overall: 3.50 ± 1.31  -G1 (3.82 ± 1.40) > G2 (3.21 ± 1.20), (Mean: 0.61), (p = 0.27)  4.4. Attempted gain (mm):  4.4.1. Mean ± SD:  -Overall: 3.76 ± 0.60  -G1 (3.77 ± 0.61) > G2 (3.75 ± 0.62), (Mean: 0.02), (p = 0.93)  *Measurements made relative to the head of the reference tenting screw  4.5. Actual gain (mm):  4.5.1. Mean ± SD:  -Overall: 3.22 ± 0.74  - G2 (3.33 ± 0.83) > G1 (3.09 ± 0.63), (Mean: -0.24), (p = 0.44)  *Measurements made relative to the head of the reference tenting screw  4.6. Resorption (mm):  4.6.1. Mean ± SD:  -Overall: 0.54 ± 0.66  -G1 (0.68 ± 0.72) > G2 (0.42 ± 0.60), (Mean: 0.27), (p = 0.34)  4.7. Graft resorption (%):  4.7.1. Mean ± SD:  -Overall: 13.89 ± 16.0  -G1 (16.91 ± 17.10) > G2 (11.12 ± 15.11), (Mean: 5.79), (p = 0.40) | *The same augmentation technique was used in both treatment groups, with the exception of the grafting material.  1. Ridge augmentation (T0) (Months):  1.1. Placing titanium reference screw  1.2. Measuring the attempted horizontal gain, to the nearest 0.5 mm, from screw head to bone.  1.3. Placing additional screws every 4-6 mm  1.4. Cortical perforation  G1:  1.5.1. Placing particulate graft consisted of a 1:1 combination of FDBA and particulated autogenous bone (Harvested from external oblique ridge, ramus or symphysis)) using a bilayer technique with the autogenous particles placed against the native bone (Placing particulate bone graft around supporting tenting screws to the level of screw heads)  1.5.2. Covering with acellular dermal matrix GBR membrane (Alloderm) (Fixed to bone with titanium tacks)  G2:  1.5. Placing particulate graft (FDBA)  2. Surgical reentry procedure (T6):  Making clinical study measurements + Implant placement | A.1.) Diameter: 1.5  A.2.) Pro-fix (Osteogenics)  A.3.) Titanium  B.1.) Standard-diameter implant  B.2.) n=33 implants in 23 sites of 21 patients | 1. CBCT:  1.1. Buccolingual dimension (Preoperative (T0))  1.2. Postop (T6)  2. Clinical evaluation:  2.1. Buccolingual dimension (Preop (T0))  2.2. Wound healing (Postop (W1, W3, T3 and T4))  3. Implant placement at desired location (Success of GBR) (T6)  4. Using a ridge-mapping caliper after reflection of flap for measuring the greatest horizontal alveolar ridge defect present at a point 3 mm apical to the alveolar crest (During first and second surgery) | 1. Patient information:  1.1. 3 of 27 patients were withdrawn from the study due to non-compliance (Final=24)  1.2. 2 patients received split-mouth treatment for 26 sites  1.3. G2 (n=2) and G1 (n=1) experienced acute postop infections (W3 postop) that resulted in loss of majority of graft and inability to place implant.  1.4. 33 implants were successfully placed in 23 sites of 21 patients  2. Average ridge width of tenting screw technique with GBR membrane (T6):  2.1. Including the 3 sites with postop infections (n=26 sites):  2.1.1. Average gain: 2.92 ± 1.08 mm (Range: 0.50 – 5.00 mm)  2.1.2. Initial: 3.63 ± 1.40 mm (Range: 1.0 – 6.5 mm)  2.1.3. Attempted gain: 3.77 ± 0.57 mm (Range: 3.0 – 5.0 mm)  2.1.4. At reentry: 6.58 ± 1.34 mm (Range: 3.0 – 9.0 mm)  2.1.5. Graft resorption: 0.85 ± 1.06 mm (Range: 0.0 – 3.5 mm)  2.2. Excluding the 3 sites with postop infections (n=23 sites):  2.2.1. Average gain: 3.22 ± 0.74 mm (Range: 2.0 – 5.0 mm)  2.2.2. Initial: 3.50 ± 1.31 mm (Range: 1.0 – 6.0 mm)  2.2.3. Attempted gain: 3.76 ± 0.60 mm (Range 3.0 – 5.0 mm)  2.2.4. At reentry: 6.72 ± 1.18 mm (Range: 4.0 – 9.0 mm)  2.2.5. Graft resorption: 0.54 ± 0.66 mm (13.89% ± 16.0%) (Range: 0.0 – 2.0 mm)  3. Horizontal ridge width gain was not affected by arch (Maxillary vs mandibular, (p = 0.31)), location (Anterior vs posterior, (p = 0.10)), edentulous space size (Small ( $\leq$ 13 mm) vs large ( > 13 mm), (p = 0.62)) or presence of adjacent teeth (Tooth bound vs non-tooth bound edentulous space, (p = 0.89))  4. Clinical measurements excluding GBR failures (n total=23 sites) (G1 (n=11), G2 (n=12)):  4.1. Age (y):  4.1.1. Mean ± SD:  -Overall: 48.95 ± 12.59  -G2 (49.50 ± 11.80) > G1 (47.45 ± 14.51), (Mean: -2.05), (p = 0.71)  4.2. Healing time (wk):  4.2.1. Mean ± SD:  -Overall: 24.34 ± 2.91  -G1 (24.65 ± 3.39) > G2 (23.85 ± 2.77), (Mean: 0.80), (p = 0.54)  4.3. Initial ridge width (mm):  4.3.1. Mean ± SD:  -Overall: 3.50 ± 1.31  -G1 (3.82 ± 1.40) > G2 (3.21 ± 1.20), (Mean: 0.61), (p = 0.27)  4.4. Attempted gain (mm):  4.4.1. Mean ± SD:  -Overall: 3.76 ± 0.60  -G1 (3.77 ± 0.61) > G2 (3.75 ± 0.62), (Mean: 0.02), (p = 0.93)  *Measurements made relative to the head of the reference tenting screw  4.5. Actual gain (mm):  4.5.1. Mean ± SD:  -Overall: 3.22 ± 0.74  - G2 (3.33 ± 0.83) > G1 (3.09 ± 0.63), (Mean: -0.24), (p = 0.44)  *Measurements made relative to the head of the reference tenting screw  4.6. Resorption (mm):  4.6.1. Mean ± SD:  -Overall: 0.54 ± 0.66  -G1 (0.68 ± 0.72) > G2 (0.42 ± 0.60), (Mean: 0.27), (p = 0.34)  4.7. Graft resorption (%):  4.7.1. Mean ± SD:  -Overall: 13.89 ± 16.0  -G1 (16.91 ± 17.10) > G2 (11.12 ± 15.11), (Mean: 5.79), (p = 0.40)  5. Similar mean graft resorption between G1 and G2 (Linearly measured) (p = 0,36) and as a percentage of attempted gain in ridge width (p = 0.37)  6. Graft healing time: 24.4 ± 2.83 weeks (Mean) |
| Jarkko T. Korpi et al (2012) / Case series | A) 22  B) 62 years (Mean) (Range 51-72)  C) 3:19 (86%)  *Inclusion criteria:  -Having a resorbed edentulous mandible of 6-mm vertical height or less at the shortest dimension (OPG)  -Edentulous  -History of conservative prosthodontic treatment failures  *Exclusion criteria:  -History of head and neck cancer, radiotherapy, chemotherapy, tobacco use,  or oral infections | Investigating the results of edentulous patients with severely resorbed mandibles who were treated with a modified tent pole procedure | - | A.1.) Anterior mandible  A.2.) Vertical  B) Vertical height $\leq$ 6 mm | 1. Ridge augmentation + implant placement (T0) (Months):  1.1. Placing dental implants in the alveolar ridge  1.2. Covering with cancellous bone (Harvested from the posterior iliac crest without the addition of PRP to the bone grafts)  1.3. Layering the bone graft with Tisseel fibrin glue  2. Uncovery (T6-8):  -Removing mucosa over each implant  -Loading with overdenture-retained prosthesis | A) No screws  B.1.) Dental implant as tent pole  B.2.) Number:  -n=4 implant per patient  -86 fixtures in total  B.3.) Endosseous dental implants  B.4.) Brand:  -Straumann ITI (n=18 patients)  -Xive (Friadent GmbH) (n=4)  B.5.) Size:  -Straumann ITI: 4.1 x 12 to 14 mm  -Xive: 3.8 x 13 to 15 mm | 1. OPG or OPTG (Height and density) (Preop (T0), immediate postop (T0+), T3 and T12)  *Vertical alveolar bone heights were determined at the shortest part of the mandible  2. Follow-up (3 to 9 years)  3. Postop visits (W2, T3, T6, T12 and annually)  4. 10-cm linear visual analog scale (D1): Acute pain level | 1. Complications:  1.1. The average acute pain level (D1): 2.5 (Range 1 - 4.5)  1.2. Uneventful postoperative courses of the patients  1.3. No surgical infections of the mandible or iliac crest sites  1.4. Patients’ oral mucosa: A normal color without ulceration or break-down after the operation  1.5. No bony dehiscences  1.6. No resorption of bone around the dental implants during follow-up  1.7. All patients had mental nerve paresthesia (T0+)  *Only 2 patients reported mild long-term changes consisting of unilateral nerve sensibility disturbances for longer than 1 year after surgery  1.8. 2 of the 88 dental implants (2.3%) were removed during the follow-up period because of over-labial positioning and immediately reimplanted (86 had correct axial inclination)  1.9. T6-8: 5 of the 88 sites (5.6%) required removal of hyperplastic soft tissue for optimal abutment placement and hygiene  2. T3:  2.1. Grafted bone density was close to that of the surrounding alveolar bone  2.2. Total bone height: Same height as the height of the dental implants at the crest of the alveolar ridge (Range 12 - 15 mm)  2.3. Alveolar ridge augmentation: 6.3 ± 1.59 mm (Mean ± SD) (Range 4 - 10 mm)  3. Uncovery (T6-8):  3.1. Ridge form: Consistently broad and wide, similar to the residual mandibular basal bone that supported the bone grafts and dental implants  3.2. No peaked or knife-edge ridges  4. Vertical mandible height (mm) (Mean (SD and 95% CI)): T0+ > T12 > T0 |
| Jerry Chin-Yi Lin et al (2024) / Retrospective | A) 44  B) 55 years (Mean) (Range 38 - 77)  C) 14 (One-third): 30 (Two-third)  *Inclusion criteria:  -Had received early VRA procedures 6-16 weeks post-extraction  *Exclusion criteria:  -Diabetic patients  -Uncontrolled systemic  diseases  -Drugs that may affect bone metabolism | A novel surgical technique of early vertical ridge augmentation for post-dental extraction | - | A.1.) Vertical  A.2.) n=50 sites  A.3.) Jaw (Sites):  -Maxilla (n=27)  -Mandible (n=23)  B) Missing teeth:  -Single missing teeth (n=30)  -Multiple adjacent missing teeth (n=20) | *Healing time between extraction and GBR: 6-16 weeks  1. GBR (T0) (Months):  1.1. Intra-marrow penetration  1.2. Inserting titanium tenting screws when indicated (At least 3 mm into the bone within the defect with the screw head flushing the future bone level)  1.3. Grafting with bone substitutes with different combinations, including mineralized allograft alone (FDBA), xenograft alone (Bovine), or allograft with a thin layer of xenograft (DBBM) on top of the allograft  1.4. Covering with cross-linked collagen membrane  2. Implant placement | A.1.) Size:  -Diameter: 1.8 mm  -Head diameter: 3 mm  A.2.) Titanium tenting screws  A.3.) Brand: WY Biomedical Co  B) NS | 1. CBCT (Vertical bone gain) (Pre (T0) and postop (T6-9))  *A line was drawn between the existing residual mesial and distal bone peaks adjacent to the defect to calculate the expected vertical bone gain (EVBG) | 1. Uneventful healing with no postoperative complications in all patients  2. Vertical bone gain (T6-9):  2.1. Total mean true vertical bone gain for all: 4.64 ± 1.67 mm  2.2. Compared to EVBG:  2.2.1. $\geq$ EVBG:  -n=40 sites, 80%  -Amount: +1.45 ± 1.47 mm  2.2.2. < EVBG:  -n=10, 20%  -Amount: - 0.96 ± 0.78 mm  3. The percentage of sites exceeding the expected vertical bone gain and the average bone gain:  3.1. Before T2:  -Sites: n=16, 62.5%  -4.63 ± 2.03 mm  3.2. After T2:  -Sites: n=34, 88.23%  -4.64 ± 1.52 mm  3.3. T2-T3:  -Sites: n=23, 86.95%  -4.62 ± 1.42 mm  3.4. Before T3:  -Sites: n=39, 76.92%  -4.62 ± 1.67 mm  3.5. After T3:  -Sites: n=11, 90.90%  -4.69 ± 1.78 mm  4. Bone level exceeding EVBG (mm):  4.1. After T2 >> Before T2, (P = 0.03)  4.2. Before T2:  -Exceeded sites: 62.5%  -Average bone gain: 4.63 ± 2.03 mm  4.3. After T2:  -Exceeded sites: 88.23%  -Average bone gain: 4.64 ± 1.52 mm  4.4. T2-T3:  -Exceeded sites: 86.95%  -Average bone gain: 4.62 ± 1.42 mm  4.5. Before T3:  -Exceeded sites: 76.92%  -Average bone gain: 4.62 ± 1.67 mm  4.6. After T3:  -Exceeded sites: 90.90%  -Average bone gain: 4.69 ± 1.78 mm (The highest)  5. Bone gain exceeding the EVBG increases over time  6. Correlation:  6.1. A positive correlation (r = 0.2377) between time and the amount of bone level gained relative to the EVBG  6.2. A slightly negative correlation between time and the average vertical bone gained in all sites |
| Sijia Yang et al (2023) / Retrospective cohort | A) 25  B.1.) Mean age: G1 (40.73 ± 12.12 years) > G2 (34.00 ± 12.25 years), (p = 0.184)  C.1.) Total: 8:17  C.2.) G1: 2:9  C.3.) G2: 6:8  C.4.) p = 0.234  *Inclusion criteria:  -Over 18 years of age at  the time of first surgery  -Horizontal alveolar bone atrophy (Identified by CBCT)  -Finished staged implant insertion  *Exclusion criteria:  -Compromised systematic health  -Uncontrolled local inflammations  -Smoking > 20 cigarettes/day  -Recent chemotherapy /radiotherapy in the maxillofacial regions | Radiographically analyzing the effects of tenting screw technique (TS) and onlay bone grafts (OG) in horizontal bone augmentation | G1) TS: Tenting screw (n=11 patients)  G2) OG: Onlay bone grafts (n=14) | A.1.) Horizontal  A.2.) Implant position:  A.2.1.) Anterior:  -G1 (n=12, 29.3%)  -G2 (n=16, 39.0%)  A.2.2.) Posterior:  -G1 (n=8, 19.5%)  -G2 (n=5, 12.2%)  A.3.) Jaw:  A.3.1) Maxilla:  -G1 (n=9, 22.0%)  -G2 (n=15, 36.6%)  A.3.2.) Mandible:  -G1 (n=11, 26.8%)  -G2 (n=6, 14.6%)  B) Leaving the screw head 3–5 mm away from the buccal bone) | A two-stage approach regarding implant placement after 4–8 months of recovery was used for all patients  1. Bone augmentation (T0) (Months):  1.1. Bone perforation  G1:  1.2.1. Insertion of tenting screws (Leaving the screw head 3–5 mm away from the buccal bone)  1.2.2. Filling the gaps underneath the screw were stuffed with a mixture of bone substitutes Bio-Oss® and autoge-  nous bone particles collected from adjacent sites  1.2.3. Covering by collagen membrane  G2:  1.2.1. Anchoring autogenous bone blocks (Harvested from mandibular symphysis or mandibular ramus) to the host bone site using the same fixation screws adopted in G1  1.2.2. Filling over and in the gaps between the autogenous bone blocks and the host bone by bone substitute Bio-Oss®  1.2.3. Covering with collagen membrane  2. Implant placement (T4-8) | A.1.) Size:  -Length: 7–10 mm  -Head diameter: 1.5 mm  A.2.) Tenting/Fixing screw  A.3.) Neodent  B.1.) n=41  B.2.) Size:  -Diameter (mm): G1 (3.85 ± 0.47 mm) > G2 (3.64 ± 0.67), (p = 0.146)  -Length (mm): G2 (12.55 ± 1.82) > G1 (11.18 ± 2.52), (p = 0.062) | 1. CBCT (Pre (T0), immediate postgrafting (T0+), Pre (T4-8) and immediate post-implantation (T4-8+)  *The distance from the buccal plate to the lingual plate was measured by drawing four reference lines (1, 3, 5, and 7 mm beneath the initial crest) perpendicular to the implant axis, and the average was calculated as the horizontal alveolar bone width (The average was calculated as the horizontal alveolar bone width (BW))  *Initial horizontal bone gain (BW1): The difference between alveolar bone widths before and after the grafting  *Horizontal bone gain during the recovery period (BW2): The difference between the horizontal bone widths post-grafting and pre-implantation  *Bone resorption rate: BV%= (BV1 - BV2)/BV1  2. Patient's medical records (Post-operative complications: Swelling, wound dehiscence, exposure of grafted materials (e.g., bone substitute particles, membrane, or tenting screws), infections, and local inflammations) | 1. Complications:  1.1. No grafting failures in either G1 or G2  1.2. After a recovery (Mean: 5.95 months): No early failure of the grafts at all  1.3. First 2 weeks after surgery: Minor swelling in patients of both groups (n=2)  1.4. First 2 weeks after surgery: graft exposure (n=1) and post-surgery inflammation (n=1) in G2  1.5. Screw exposure: G1 (n=2)  1.6. During the postoperative follow-up periods: 8 minor complications, but all were fully resolved  2. None of the basic characteristics of the patients (e.g., age, gender, smoking habits, his-  tory of periodontitis, and healing periods) or implants (e.g., implant position, jaw position, implant diameter, and implant length) showed significant differences  3. Horizontal and volumetric bone gain:  3.1. T0 - T0+:  3.1.1. Horizontal bone gain (BW1): G1 (6.15 ± 2.12) >> G2 (4.86 ± 1.40), (p = 0.001)  3.1.2. Volumetric bone gain (BV1): G2 (811.77 (459.89, 987.84)) > G1 (748.53 (371.58, 1037.84)), (p = 0.128)  3.2. T0 - T4-8:  3.2.1. Horizontal bone gain (BW2): G1 (4.61 ± 2.06) >> G2 (2.87 ± 1.58), (p = 0.000)  3.2.2. Volumetric bone gain (BV2): G1 (607.47 (292.05, 848.25)) > G2 (508.49 (342.9, 645.795)), (p = 0.504)  3.2.3. Bone resorption rate (BV%): G2 (29.38 (24.82, 42.13)) >> G1 (21.34 (1.54, 32.70)), (p = 0.000)  3.3. No significant difference was detected for the two groups regarding 3-dimensional bone gain immediately at the two timepoints through GEE analysis (P > 0.05)  4. Average alveolar bone width:  4.1. T0: G1 (3.69 ± 1.36 mm) > G2 (3.60 ± 1.40 mm)  4.2. T0+: G1 (9.84 ± 2.03 mm) >> G2 (8.45 ± 1.76 mm), (P < 0.05)  4.3. After recovery period: G1 (8.30 ± 2.05 mm) >> G2 (6.47 ± 2.12 mm), (P < 0.05)  4.4. When compared with G2, the favorable bone gain was observed in G1 immediately after the operation and after the recovery period (P < 0.05) |
| Thomas Stumpf et al (2024) / Case series | A) Initial: 279  Final: 228  B) Mean:  B.1.) Men: 52.32 ± 15.11 years  B.2.) Women: 56.71 ± 13.06 years  C) Final: 102:126  *Inclusion criteria:  -At least 1 site with a horizontal and / or vertical ridge defect  -No infection in recipient site  *Exclusion criteria:  -Medical and / or general contraindications for intraoral surgical procedures  -Pregnant  -Nursing patients  *Smoking habit:  -Nonsmoker (n=199)  -Smokers:  < 10 cigarettes/day (n=10)  > 10 cigarettes/day (n=19) | Ridge augmentation using the umbrella (US) technique in different defect morphologies | G1) US1: All USs in situ at reentry (n=201)  G2) US2: Several USs had to be removed prematurely (n=14)  G3) US3: All USs had to be removed prematurely (n=13) | A.1.) Number of sites:  -Initial: 279  A.2.)  Total:  -Vertical (n=25)  -Horizontal (n=167)  -Combined (n=36)  G1:  -Horizontal (n=157)  -Vertical (n=18)  -Combined (n=26)  A.3.) Jaw:  -Maxilla (n=132, 57.89%)  -Mandible (n=96, 42.11%)  B) NS | 1. Ridge augmentation (T0) (Months):  1.1. Placing USs (Providing an additional 1 mm of space)  *No decortication or perforation  *Amount of augmentation: From the bottom of the screw head to the farthest bone margin  1.2. Filling the defect volume with particulate graft material (GM)  *GM compromised either:  -Autologous bone chips (AB) alone (Harvested from ramus)  -A mixture of AB with xenogeneic bone substitute (AB/XBS) (1:1)  -Allogeneic (ABS) Maxgraft alone (Human Spongiosa CHB)  1.3. Mixing GM with A-PRF membrane, cut into pieces in combination with i-PRF  1.4. Covered with native collagen membrane  2. Reentry (T6 (AB or ABS), T9 (AB/XBS)):  Screw removal + Implant insertion  3. Prosthetic restoration  *Baseline: Distance from the head of the US to bone level in the horizontal (US-hd) as well in the vertical dimension (US-vd)  *Reentry: Distance from the head of the US to the newly formed bone (US-hnb and US-vnb)  *Achieved horizontal bone gain (bg-h)= US-hd $-$ US-hnb  (Calculating the vertical bone gain (bg-v) in a similar way)  *Horizontal target performance index (TPI): TPI-h= (bg-h) / (US-hd) $\times$ 100%  (Calculating TPI-v in a similar way) | A.1.) Umbrella tenting screw  A.2.) Surgical steel  A.3.) Size:  -Diameter: 1.2 mm  -Head diameter: 4, 5 or 6 mm  -Length: 8, 10, 12 or 13 mm  A.4.) Number:  -n=1-8 screws per site  -n=463 total  *Most defects (89%) could be augmented with 3 or less USs  A.5.) Brand: Ustomed-Schirmschrauben, Geistlich  B.1.) Brand: Astra EV  B.2.) n=274  B.3.) Implant insertion torque in augmented bone: 29.59 $\pm$ 19.38 Ncm  B.4) Size:  -Diameter: 3.6-4.2 mm  -Length: 9 mm | 1. Clinical evaluation (T6)  2. CBCT (T0 and subsequent implantation)  3. Assessing early (Before prosthetic restoration placement) and late (until 09/2023) implant loss, the insertion torque, and the bone density in all non-simultaneous cases  4. Bone density (On drilling by surgeon’s tactile sense): D1 to D4 (Misch criteria)  5. Insertion torque (By surgical motor)  6. Radiograph (Pre (T0) and subsequent implantation) | 1. Number of patients:  1.1. Initial: 279  1.2. Final: 228  1.3. Reason: Death (n=1) and excluded (n=50) (Implant placement / reentry alio loco (n=11) and reentry pending (n=39))  2. Analysis:  2.1. G2 (n=14)  2.1.1. No second augmentation needed, implantation possible (n=6)  2.1.2. Second augmentation needed, implantation possible (n=8)  2.1.3. Second augmentation needed, no implantation possible (n=0)  2.2. G3 (n=13)  2.2.1. No second augmentation needed, implantation possible (n=3)  2.2.2. Second augmentation needed, implantation possible (n=9)  2.2.3. Second augmentation needed, no implantation possible (n=0)  2.2.4. Patient rejected further treatment (n=1)  3. Simultaneous implantation in 58 cases  4. Healing time:  4.1. AB or ABS: T6 (6 months)  4.2. AB/XBS: T9 (9 months)  5. Augmentation dimension:  5.1. Vertical (5.47 $\pm$ 2.08 mm) (Range 1-12 mm)  5.2. Horizontal (5.10 $\pm$ 1.48 mm) (Range 2-10 mm)  6. Analysis 1:  6.1. Early exposure (US1 + US2 + US3) (Wound dehiscence during the first 2 weeks after surgery):  6.1.1. Cases: n=39, 17.10%  6.1.2. No significant risk: Age (p = 0.208), soft tissue thickness (p = 0.891), smoking habit (p = 0.202), grafting material (p = 0.703) and location of the site of augmentation in the maxilla or mandible (p = 0.837)  6.1.3. Significant risk: Male sex (p = 0.02), vertical defect component (0.000) and a greater extent of vertical augmentation (p = 0.048)  6.2. Late exposure (US1 + US2) (Wound dehiscence after the first 2 weeks after surgery):  6.2.1. Cases: n=15, 6.98%  6.2.2. No significant risk: Age (p = 0.908), sex (p = 0.203), soft tissue thickness (p = 0.696), smoking habit (p = 0.252) and location of the augmentation (p = 0.371)  6.2.3. Material:  -AB (n total=11, n late exposure=1)  -ABS (n total=155, n late exposure=13)  -AB/XBS (n total=49, n late exposure=1)  -n total late exposure=15  -Use of AB/XBS correlates with a lower risk.  6.2.4. Vertical component of defect had a significantly higher risk for late exposure (p = 0.024) independent of the extent of the US-vd (p = 0.214)  6.2.5. Significantly lower risk for premature screw removal if the defect had only a horizontal component (p = 0.000)  6.2.6. The extent of horizontal augmentation increased risk for a late exposure (p = 0.040) significantly  7. Analysis step 2 (G1)  7.1. Mean extent of augmentation:  -Horizontal (US-hd) (n=183): 5.01 $\pm$ 1.49 mm  -Vertical (US-vd) (n=44): 5.32 $\pm$ 1.88 mm  7.2. Mean resorption rate:  -Horizontal (US-hnb): 0.85 $\pm$ 0.91 mm  -Vertical (US-vnb): 1.21 $\pm$ 1.03 mm  -No significant influence: Age (h (p = 0.543), v (p = 0.607), sex (h (p = 0.475), v (p = 0.242)), smoking (h (p = 0.569), v (p = 0.371)), extent of augmentation (h (p = 0.318), v (p = 0.983)), defect morphology and soft tissue thickness (h (p = 0.962), v (p = 0574))  -ABS (1.33 $\pm$ 1.05 mm) > AB/XBS (0.72 $\pm$ 0.83 mm), (p = 0.113)  -GMs showed no significant difference (p = 0.953) in relation to horizontal resorption rate.  -Mandible (1.07 $\pm$ 0.87 mm) >> Maxilla (0.71 $\pm$ 0.91 mm), (p = 0.009)  -Horizontal resorption rate was not influenced by early exposure (p = 0.635)  -Vertical resorption with early exposure (1.88 $\pm$ 0.96 mm) >> without exposure (1.01 $\pm$ 0.97 mm), (p = 0.023)  -Significant correlation between late exposure and greater horizontal (1.31 $\pm$ 0.45 mm), (p = 0.027) and greater vertical (0.97 $\pm$ 0.25 mm), (p = 0.030) resorption.  7.3. Bone gain:  7.3.1. Mean:  -Horizontal (n=183): 4.23 $\pm$ 1.69 mm  -Vertical (n=44): 4.11 $\pm$ 1.99 mm  7.3.2. The older the patient, the less horizontal (p = 0.041) but more vertical (p = 0.024) bone gain  *Inhomogenous distribution by regression (Weak correlation with moderate risk of bias)  7.3.3. Significant influence: Smoking (h (p = 0.022)), thicker soft tissue (Greater horizontal gain (p = 0.037))  7.3.4. No significant influence: Smoking (v (p = 0.543)), sex, thicker soft tissue (v (p = 0.096)) and early exposure (h (p = 0.772), v (p = 0.594))  7.3.5. Horizontal bone gain:  -Maxilla >> mandible, (p = 0.000)  -ABS >> AB or AB/XBS, (p = 0.007)  -Not influenced by late exposure (p = 0.668)  7.3.6. Vertical bone gain:  -Mandible > maxilla, (p = 0.166)  -ABS vs AB or AB/XBS (p = 0.923)  -Presence of vertical component resulted in significantly less horizontal bone gain (0.81 mm less on average), (p = 0.042)  -Correlation between late exposure and less vertical bone gain: 1.67 mm (Mean), (p = 0.035)  7.3.7. The more bone was augmented in a vertical or horizontal direction, the more bone gain was achieved (p = 0.000)  8. Implant loss:  8.1. Early:  8.1.1. Removing 3 of 274 implants after augmentation in a second stage (n=3 patients) before placement of the prosthetic restoration (Reason: infection)  8.1.2. Placing new implants after healing (n=2 cases)  8.1.3. All these implants are still in function  8.2. Late: 8.2.1. n=1 implant after placement of prosthetic restoration until September 2023  9. Bone density:  -D1 (n=11 implants)  -D2 (n=73)  -D3 (n=157)  -D4 (n=33)  10. TPI:  10.1. Vertical:  10.1.1. Number of implants: n=44 (Valid), n=157 (Missing)  10.1.2. Percentage (Mean $\pm$ SD): 75.90 $\pm$ 20.54 %  10.1.3. Absolute (Mean $\pm$ SD): -1.21 $\pm$ 1.03 %  10.1.4. An early exposure was linked to a worse TPI in general and the mean difference of 0.87 in absolute vertical TPI was significant (P = 0.016)  10.2. Horizontal:  10.2.1. Number of implants: n=183 (Valid), n=18 (Missing)  10.2.2. Percentage (Mean $\pm$ SD): 82.35 $\pm$ 19.00 %  10.2.3. Absolute (Mean $\pm$ SD): -0.84 $\pm$ 0.91 %  10.2.4. The extent of horizontal augmentation significantly influenced the percentage TPI horizontally (P = 0.012)  *Other dimensions of the augmentation had no influence on TPI.  10.3. Age, sex, smoking habit and soft tissue thickness had no statistical influence on the TPI.  10.4. Influence of late exposure on TPI:  10.4.1. Vertical percentage: No (n=39) (78.73 $\pm$ 19.21) >> Yes (n=5) (53.83 $\pm$ 18.51), (P = 0.036)  10.4.2. Vertical absolute: No (n=39) (-1.10 $\pm$ 1.02) >> Yes (n=5) (-2.07 $\pm$ 0.69), (P = 0.030)  10.4.3. Horizontal percentage: No (n=176) (83.01 $\pm$ 18.65) >> Yes (n=7) (65.79 $\pm$ 21.59), (P = 0.080)  10.4.4. Horizontal absolute: No (n=176) (-0.79 $\pm$ 0.87) >> Yes (n=7) (-2.11 $\pm$ 1.19), (P = 0.026)  Late exposure was correlated with a highly significant change in TPI. If a late exposure was detected, the mean TPI was 24.9 %worse for the vertical and 17.22 % worse for the horizontal dimension. |
| Nam-Suk Yoon (2025) / Retrospective clinical study | A) 13  B) 52.4 years (Mean) (Range 44 - 71)  C) 5:8  *Inclusion criteria:  -Edentulous posterior mandibular regions with ≥5 mm vertical ridge deficiency (Confirmed by CBCT and panoramic)  -Requiring vertical ridge augmentation to support prosthetic rehabilitation using multiple splinted implants  *Exclusion criteria:  -Uncontrolled systemic conditions affecting bone healing (e.g., diabetes mellitus, osteoporosis)  -Active infection  -History of bisphosphonate use, chemotherapy, or radiotherapy within the past year | Evaluating the clinical and radiographic effectiveness of WHTPS for vertical ridge augmentation, particularly in cases with severe vertical bone deficiency | - | A.1.) Vertical  A.2.) Posterior mandible  B) Minimum vertical bone defect: 5 mm  2. Bone:  2.1. Mean augmented bone height (T0+): 8.86 mm ± 2.59 mm (Mean ± SD), (Range: 5.30 – 13.15 mm)  2.2. Height (T0): 6.1 mm ± 0.7 mm (Mean ± SD), (Range: 5.0 - 7.2 mm)  2.3. Resorption at follow-up: 1.49 ± 0.78 mm (Mean ± SD), (Range: 0.28 – 2.91 mm) (Accounting for 17.79% of the initially augmented height) | 1. Augmentation + implant placement (T0) (Months):  1.1. Under-osteotomy (A drill 1 mm narrower than the implant diameter)  1.2. Placing implants 2 mm subcrestally in the edentulous ridge (As tenting structures)  1.3. Connecting a tenting pole abutment to the implant platform (Torqued to 10 Ncm)  1.4. Placing a wide-head tent-pole screw (WHTPS) at the pontic site into the buccal or lingual cortical bone in (In the area of the most severe vertical defect, where implant placement was avoided to prevent inferior alveolar nerve injury)  *Inserting WHTPS using a 0.48-inch hex driver attached to an implant motor at 40 rpm  1.5. Grafting sticky autogenous bone (Autogenous bone (Harvested from alveolar ridge) + CGF-derived fibrin glue) around the exposed implant threads, WHTPS, and defect area  1.6. Layering over the autogenous bone and around remaining area by sticky bovine bone  *Fully covering both the tenting pole abutment and WHTPS by graft material  1.7. Placing a resorbable collagen membrane over the graft  1.8. Placing CGF membrane  *No membrane tacks or fixation sutures  2. Uncovering surgery + WHTPS removal (T4)  3. Provisional restoration (T5 + 3 weeks later)  4. Zirconia-based definitive prosthesis (T7) | A.1) WHTPS:  -Length: 12-14 mm  -Width/Diameter: 6-8 mm  A.2.) SBB®, Biotem Implant  A.3.) Tenting pole abutment:  -Height: 2 mm  -Torque: 10 Ncm  B.1.) Biotem Implant  B.2.) SANTA, Biotem Implant tent pole abutments | 1. CBCT:  1.1. Preop (T0)  1.2. Immediate postop (T0+): Native and augmented bone heigh  1.3. Uncovering (T4)  2. Panoramic:  1.1. Preop (T0):  1.2. Immediate postop (T0+): Native and augmented bone heigh  1.3. T7: Evaluating augmented bone and resorption over 3–7 months (Mean: 5.5 months)  1.4. Implant uncovering (T4)  1.5. Final prosthesis delivery (T7)  1.6. 2 years after functional loading (T31)  *Reference line: A baseline was drawn parallel to the long axis of the adjacent teeth, with the top of the tent-pole screw  *Measurement 1 (Total bone height): Distance from the lowest point of the mandible (Mandibular base) to the top of the tent-pole screw  *Measurement 2 (Augmented bone height): Distance from the top of the tent-pole screw to the grafted junction  *Extension of Measurement 2 (Pre-augmented bone height): Distance from the mandibular base to the graft junction  3. Follow-up (T11-13)  * follow-up period ranged from 8 to 24 months (mean: 14.9 months) | 1. Complications:  1.1. Soft tissue dehiscence or WHTPS exposure (n=1)  1.2. No neurosensory disturbances (Paresthesia or dysesthesia) and no significant complications (Aside from postoperative swelling at the recipient sites)  1.3. All implants, including the one case that required secondary grafting, were successfully uncovered at the designated time point  1.4. No implants mobility or failure during follow-up period (Mean: 14.9 months, Range: 8 – 24)  1.5. Implant survival rate: 100%  1.6. 12 of the 13 cases healed uneventfully, without signs of infection, inflammation, or peri-implant bone loss  1.7. Partial wound exposure (2 weeks postop) (n=1): Approximately 40% graft resorption (Managed with a secondary grafting procedure performed one month later, and healing thereafter progressed without further complications)  2. Bone:  2.1. Mean augmented bone height (T0+): 8.86 mm ± 2.59 mm (Mean ± SD), (Range: 5.30 – 13.15 mm)  2.2. Height (T0): 6.1 mm ± 0.7 mm (Mean ± SD), (Range: 5.0 - 7.2 mm)  2.3. Resorption at follow-up: 1.49 ± 0.78 mm (Mean ± SD), (Range: 0.28 – 2.91 mm) (Accounting for 17.79% of the initially augmented height)  3. Significant difference between the immediate postoperative and follow-up bone heights (p = 0.0002)  4. Stable integration of the grafted material without signs of collapse, migration, or structural compromise despite minor resorption  5. WHTPS effectively stabilized the graft in pontic regions, supporting predictable vertical ridge augmentation |
| Bach Le (2010) /  Prospective case study | A) 15  B) 50.06 years (Mean) (Range 22 - 69)  C) 2:13  *Inclusion criteria:  -Partially edentulous patients seeking dental implant therapy who required more than 7 mm of vertical alveolar ridge augmentation before implant placement  -Less than 4 mm of bone width (Determined by preoperative examination and CT imaging)  -Before augmentation, all grafted sites were deemed inadequate because of anatomical constraints for placement of an implant of at least 10 mm in length  *Exclusion criteria:  -Smokers  -Diabetic patients  -Any medically  compromised patients | Evaluating the effectiveness of using titanium screws in  combination with particulate human mineralized allograft, in a “tenting” fashion, to augment large vertical alveolar ridge defects for implant placement | - | A.1.) Vertical  A.2.) Jaw:  -Maxilla (n=9 patients)  -Mandible (n=6)  A.3.) 2 or more missing teeth with severe vertical defects of the maxillary  alveolar ridge (n=12)  A.4.) Anterior and posterior  B) Large ( > 7 mm) localized alveolar ridge defects  *Required more than 7 mm of vertical alveolar ridge augmentation  Exposing approximately 5 - 7 mm of screw threads) | 1. Ridge augmentation (T0) (Months):  1.1. Placing titanium screws in the alveolar ridge (Exposing approximately 5 - 7 mm of screw threads)  1.2. Completely covering the screw by mixture of particulate human mineralized allograft (Cancellous particles) with the patient’s blood  1.3. Overcorrecting with particulate material  1.4. Placing a resorbable membrane  2. Uncovering grafted sites + Screw removal + Implant placement (T4-5):  -Adding additional allograft material  -27 implants had single-staged protocol and 5 implants needed uncovering  3. Uncovering 5 implants    *A waiting period of at least 3 months before final restoration  4. Final restoration (T15) | A.1.) Titanium screws  A.2.) 1.5 mm  A.3.) KLS Martin, Jacksonville, FL  B.1.) n=32  B.2.) Implant systems:  -BioHorizons implants (n=6)  -Straumann implants (n=15)  -AstraTech implants (n=7)  -Zimmer implants (n=4)  B.3.) Jaw (Implants):  -Maxilla (n=16)  -Mandible (n=16) | 1. Histologic analysis (Harvesting bone core) (T4-5) (All)  2. Histomorphometric analysis (T4-5) (n=7 specimens)  3. CT (Bone width) (Pre (T0) and postop (T4-5))  4. Panorex (T4-5 and after all implants were restored at follow-up intervals of 6 and 12 months)  5. PA (After all implants were restored at follow-up intervals of 6 and 12 months) | 1. 12 patients underwent extractions of adjacent teeth to allow a more favorable bone attachment to which to graft  2. Complications:  2.1. No postoperative wound infections  2.2. Complete dehiscence of the grafted site with graft and screw exposure requiring subsequent graft (n=1 patient)  *Although there was complete exposure of the graft material, partial graft take was noted upon re-entry after 4 months  2.3. Partial wound dehiscence (n=2)  2.4. Screw head exposure only (n=3)  2.5. Partial graft loss on re-entry with wound complete or partial wound dehiscence (n=4)  2.6. All the complications were treated and no cases were excluded.  3. Complete coverage of the screw head by bone was noted (n=11)  4. Ridge width was clinically evaluated to be larger than 6 mm at all sites of implant placement  5. Follow-up (16.8 months after implant placement, (Range, 4 – 38 months)):  5.1. All implants have been successfully restored in the 15 patients  5.2. Stable and healthy peri-implant tissue and bone levels  6. After 3 to 4 months of integration all implants were noted to be integrated.  7. Histology:  7.1. All cores showed good integrity with a good cancellous bone pattern and good connectivity of the trabeculae  7.2. The new bone formation had surrounded the mineralized allograft particles and had formed bridges resulting in a good cancellous bone pattern  8. High-power images:  8.1. Excellent integration of new bone formation and particles of mineralized allograft  8.2. All grafted sites consisted of viable bone  9. Histomorphometric analysis (n=7): Mean bone content: 43% (Of this percent bone, the mean vital bone content was 81%)  10. Mean vertical augmentation: 9.7 mm |
| Siyuan Wang et al (2025) / Retrospective cohort | A) 42  B) 37.88 ± 13.19 years (Mean)  C) 14:28  *Inclusion criteria:  -Healthy or treated periodontal conditions  -Insufficient bone width (< 4 mm at 1 mm below the alveolar ridge crest at the implant site)  -Patients who received horizontal bone augmentation and simultaneous implantation in the anterior maxilla  -Patients who completed prosthetic procedures  *Exclusion criteria:  -Heavy smokers (≥ 10  cigarettes/day)  -Patients unable to complete prosthetic  procedures or follow- up examinations. | Evaluating the 1- to 5- year outcomes of dental implants placed with the tenting screw (TS) technique and comparing their clinical efficacy with conventional guided bone regeneration (GBR) | G1) TS: Tenting screw (n=20)  G2) GBR: Conventional GBR (n=22) | A.1.) Anterior maxilla  A.2.) Horizontal  B.1.) Bone defect size (Units):  -1–3 (n=31)  -4–6 (n=11)  B.2.) Bone defect classification (Benic horizontal defect classification evaluations)  -Class 2 (n=19)  -Class 3 (n=23)  Protruding approximately 4 mm from the buccal plate)  1. Initial bone (mm) (mean ± SD):  1.1. Width: G1 (2.78 ± 1.06) > G2 (2.68 ± 0.94), (p = 0.736)  1.2. Height: G1 (15.08 ± 1.45) > G2 (14.88 ± 1.09), (p = 0.855)?!  7. Horizontal bone gains (mm):  7.1. BW0:  7.1.1. T0+: G1 (2.92 ± 1.39) > G2 (2.81 ± 1.29)  7.1.2. T0.5: G1 (2.22 ± 1.14) > G2 (1.73 ± 0.93)  7.1.3. Follow up: G1 (1.89 ± 1.13) > G2 (1.32 ± 0.81)  7.2. BW1:  7.2.1. T0+: G1 (3.58 ± 1.63) > G2 (3.29 ± 1.70)  7.2.2. T0.5: G1 (2.85 ± 1.42) > G2 (1.95 ± 1.35)  7.2.3. Follow up: G1 (2.41 ± 1.41) > G2 (1.44 ± 1.09)  7.3. BW3:  7.3.1. T0+: G1 (4.40 ± 1.74) > G2 (3.78 ± 1.69)  7.3.2. T0.5: G1 (3.37 ± 1.79) > G2 (2.06 ± 1.69)  7.3.3. Follow up: G1 (2.78 ± 1.75) > G2 (1.42 ± 1.56)  7.4. BW5:  7.4.1. T0+: G1 (4.25 ± 1.32) > G2 (3.63 ± 1.09)  7.4.2. T0.5: G1 (3.27 ± 1.68) > G2 (2.03 ± 1.75)  7.4.3. Follow up: G1 (2.53 ± 1.69) > G2 (1.28 ± 1.68)  7.5. Horizontal bone resorption (BW3 and BW5): G2 >> G1, (*p* < 0.05) | 1. Implant placement + Augmentation (T0) (Years):  *Primary stability of 15Ncm of all the included implants was guaranteed  1.1. Osteotomy and implant placement  1.2. Installing healing abutment (Acting as a tent­ like support)  1.3. Perforating fenestrated or dehiscent buccal bone plate  G1:  1.4. Inserting tenting screws on the buccal side  of the bony defect (Protruding approximately 4 mm from the buccal plate)  1.5. Inserting margin of absorbable collagen membrane under the palatal mucoperiosteal flap  1.6. Filling the space beneath the tenting screws with a 1:1 mixture of deproteinized bovine bone mineral (DBBM) and particulate autogenous bone (Harvested from adjacent sites)  1.7. Covering with collagen membrane (Double­ layer technique)  G2:  1.4. Inserting margin of the collagen membrane under the palatal mucoperiosteal flap  1.5. Placing a 1:1 mixture of DBBM and particulate autogenous bone (Harvested from adjacent sites) directly into the bony defect  1.6. Placing collagen membrane (Double­ layer technique)  2. Re-entry surgery + Prosthesis delivery (T0.5):  -Undercontoured, screw­ retained provisional crowns  -Mono-lithic or multi- layered zirconia crowns  -Concave shape  -Emergence angle < 30  -For patients with only slight deficiency in the buccal soft tissue volume, a palatal incision was made at the re- entry surgery, pushing the palatal soft tissue toward the buccal side to enhance buccal fullness. For patients with an inadequate keratinized gingiva width (< 2 mm), an autogenous free epithelized mucosal graft harvested from the palate was performed. For patients exhibiting discolorations due to the presence of thin mucosa thickness, a tunnel approach combined with an autogenous connective tissue graft was adopted to thicken the mucosa at implant sites.  *Tenting screws were removed once exposed, without influencing bone augmentation effects. | A.1.) Size:  -Length: 7–10 mm  -Head diameter: 1.5 mm  A.2.) Fixing screw  A.3.) Neodent  B.1.) n=75  B.2.) Healing abutment height: 2 mm  B.3.) Implant brand:  -Straumann (n=46)  -Nobel (n=23)  -Astra (n=6)  B.4.) Implant diameter (mm):  - ≤ 3.75 (n=70)  - > 4 (n=5)  B.5.) Implant length (mm):  - ≤ 12 (n=15)  - > 12 (n=60) | 1. CBCT:  1.1. Preop (T0)  1.2. Immediate postop (T0+): Confirming the  three- dimensional position of the implants  1.3. Post-healing (T0.5): Confirming osseointegration  1.4. Follow-up  *Measurement plane: The one where the implant circumference was maximized  *Horizontal bone widths (At different time points (T0, T0+, T0.5, and follow-up)): At the implant shoulder level and 1, 3, and 5 mm below it  2. Check-up (T0.5, once a year (1-5 years)):  -G1: 3.39 ± 1.37 years  -G2: 3.74 ± 1.32 years  3. Clinical evaluation:  3.1. Surgical (Medical records): Swelling, incisional dehiscence, bone powder or membrane exposure, titanium screw exposure, and infection  3.2. Mechanical (Present or absent): Abutment screw loosening, loss of retention, loss of adjacent contact, chipping of ceramic, and abutment fracture  *Implant survival rate: The percentage of implants remaining in situ  3.3. Biological complication:  3.3.1. Peri- implant health and oral hygiene: Implant mobility, plaque index (PlI), suppuration and/or bleeding on probing (SoP and/or BoP) and probing pocket depth (PPD) at four aspects of each implant (mesio-buccal, buccal, disto­ buccal, and lingual)  3.3.2. Peri­implant mucositis: Presence of suppuration /bleeding by gentle probing and/or registration of fistula in the absence of bone loss after initial bone remodeling  3.3.3. Peri­implantitis: Combination of suppuration/bleeding by gentle probing, probing depth exceeding 5 mm, and a crestal bone level located at least 3 mm apical to the most coronal portion of the intraosseous part of the implant  3.4. Pink esthetic score and white esthetic score (PESWES) (Photographs) (Follow-up): 0 (Worst esthetic outcome) to 20 (Perfect esthetic outcome)  * Recorded variables included the following (2 dimensional):  -Bone width (BWa): horizontal bone width measured at Tn, where ‘a’ represents the implant shoulder level and its respective positions below (1 mm, 3 mm, and 5 mm), and ‘n’ represents T0,T1, T2, and T3.  -BWa gain: horizontal bone augmentation at Tn, representing the difference between the alveolar bone width at each of the four horizontal levels at Tn and the initial alveolar bone width.  -△BwaHeal: horizontal bone resorption during healing periods, calculated as BWgainT1 -BWgainT2.  -△BwaFollow­ up: horizontal bone resorption during follow- up periods, calculated as BWgainT2 –BWgain  *3 dimensional:  -BV gain: site- specific volumetric bone augmentation at each time point, with CBCT at T0 serving as the reference, adjusted for differences in bone defect size by dividing the  measured total volume by the defect units.  -BV%Heal: volumetric bone resorption during healing periods, calculated as (BVgainT1-  ­ BVgainT2)/BVgainT1.  -BV%Follow­ up: volumetric bone resorption during follow- up periods, calculated as (BVgainT2-  ­ BVgainT3)/BVgain | 1. Initial bone (mm) (mean ± SD):  1.1. Width: G1 (2.78 ± 1.06) > G2 (2.68 ± 0.94), (p = 0.736)  1.2. Height: G1 (15.08 ± 1.45) > G2 (14.88 ± 1.09), (p = 0.855)  2. No significant differences in potential variables between G1 and G2, including gender, age, history of periodontitis, smoking habits, gingival biotype, keratinized gingival width, and implant size (*p* > 0.05)  3. During the follow­ up period from T0 to the latest follow­ up (3.39 ± 1.37 years (G1) and 3.74 ± 1.32 years (G2)):  3.1. A total of 75 inserted implants remained stable and functional  3.2. Survival rate: 100% in both  4. Complication:  4.1. Local swelling within 3–5 days post- surgery in all patients (Resolved)  4.2. Bone substitute material exposure (n=1 patient in G2) (Resolved)  4.3. Tenting screw exposure (Over the follow- up period after functional loading (mean: 2.38 ± 1.52 years, (Range: 0.92 ~ 4.50 years))) (n=5 patients in G1) (Removed under local anesthesia)  4.4. Abutment screw loosening (n=1 in G2)  4.5. Veneering ceramic chipping (n=1 in G1)  4.6. All complications were carefully addressed during follow- up examinations, with no adverse effects on the long­ term stability of peri­ implant soft and hard tissues  5. Mean loading time:  -G1: 2.86 ± 1.40 years  -G2: 3.18 ± 1.32 years  6. Radiographic outcomes:  6.1. T0+ - T0.5:  6.1.1. △BW0 Heal (mm): G2 (1.09 ± 0.77) > G1 (0.80 ± 0.79), (p = 0.705)  6.1.2. △BW1 Heal (mm): G2 (1.34 ± 1.04) > G1 (0.73 ± 0.68), (p = 0.374)  6.1.3. △BW3 Heal (mm): G2 (1.72 ± 1.24) >> G1 (1.06 ± 0.87), (p = 0.032)  6.1.4. △BW5 Heal (mm): G2 (1.60 ± 1.05) >> G1 (0.98 ± 0.90), (p = 0.009)  6.1.5. BV% Heal: G2 (29.3% (18.1%, 39.6%)) >> G1 (16.5% (9.32%, 28.7%)), (p = 0.001)  6.2. T0.5 – follow up:  6.2.1. △BW0 Follow­ up (mm): G2 (0.41 ± 0.37) > G1 (0.33 ± 0.36), (p = 0.806)  6.2.2. △BW1 Follow­ up (mm): G2 (0.52 ± 0.39) > G1 (0.45 ± 0.34), (p = 0.961)  6.2.3. △BW3 Follow­ up (mm): G2 (0.64 ± 0.35) > G1 (0.59 ± 0.54), (p = 0.718)  6.2.4. △BW5 Follow­ up (mm): G2 (0.75 ± 0.47) > G1 (0.73 ± 0.51), (p = 0.449)  6.2.5. BV% Follow­ up: G2 (29.5% (19.9%, 34.2%)) >> G1 (20.6% (10.7%, 29.7%)), (p = 0.015)  6.3. T0+ - follow up:  6.3.1. BV%: G2 (50.7% (41.9%, 57.5%)) >> G1 (36.7% (28.2%, 44.9%)), (p = 0.001)  *From 6.1.1 to 6.1.4 and from 6.2.1 to 6.2.4: Comparison between groups using GLMM with group, age, gender, smoking habits, history of periodontitis, follow­ up period, implant brand, implant diameter, and length as fixed effects, including patient random effects.  *6.1.5, 6.2.5 and 6.3.1: Comparison between groups using GLMM with group, age, gender, smoking habits, history of periodontitis, follow- up period, bone defect size, and classification as fixed effects.  7. Horizontal bone gains (Compared with T0) (mm):  7.1. BW0:  7.1.1. T0+-T0: G1 (2.92 ± 1.39) > G2 (2.81 ± 1.29)  7.1.2. T0.5-T0: G1 (2.22 ± 1.14) > G2 (1.73 ± 0.93)  7.1.3. Follow up-T0: G1 (1.89 ± 1.13) > G2 (1.32 ± 0.81)  7.2. BW1:  7.2.1. T0+-T0: G1 (3.58 ± 1.63) > G2 (3.29 ± 1.70)  7.2.2. T0.5-T0: G1 (2.85 ± 1.42) > G2 (1.95 ± 1.35)  7.2.3. Follow up-T0: G1 (2.41 ± 1.41) > G2 (1.44 ± 1.09)  7.3. BW3:  7.3.1. T0+-T0: G1 (4.40 ± 1.74) > G2 (3.78 ± 1.69)  7.3.2. T0.5-T0: G1 (3.37 ± 1.79) > G2 (2.06 ± 1.69)  7.3.3. Follow up-T0: G1 (2.78 ± 1.75) > G2 (1.42 ± 1.56)  7.4. BW5:  7.4.1. T0+-T0: G1 (4.25 ± 1.32) > G2 (3.63 ± 1.09)  7.4.2. T0.5-T0: G1 (3.27 ± 1.68) > G2 (2.03 ± 1.75)  7.4.3. Follow up-T0: G1 (2.53 ± 1.69) > G2 (1.28 ± 1.68)  7.5. Horizontal bone resorption (BW3 and BW5): G2 >> G1, (*p* < 0.05)  8. CON% (After follow up): G1 (99.68% ± 0.71%) > G2 (99.58% ± 0.83%), (*p*  = 0.578)  *Comparable implant- bone contact at the latest follow- up  9. Three­ dimensional bone volume per defect unit:  9.1. T0+: G2 (247.15 (167.96, 334.06) mm3) > G1 (246.90 (182.64, 351.22) mm3)  9.2. After an average healing period of 6.56 ± 1.71 months:  9.2.1. Bone gain: G1 (201.84 (136.76, 312.58) mm3) > G2 (140.31 (105.27, 197.33) mm3)  9.2.2. BV% Heal: G2 (29.3%) >> G1 (16.5%), (*p* < 0.001)  9.3. Over a follow- up period of 1–5 years:  9.3.1. BV% Follow- up: G2 (29.5%) >> G1 (20.6%), (*p* < 0.05)  10. Biological outcomes:  10.1. Overall median PPD (mm): G2 (2.5 (2.25, 2.75)) >> G1 (2.50 (2.25, 2.50)), (p = 0.038)  10.2. Overall median BoP (%): G2 (25 (0, 50)) > G1 (0 (0, 25)), (p = 0.421)  10.3. Overall median PlI (%): G2 (25 (0, 50)) > G1 (25 (0, 43.75)), (p = 0.933)  10.4. Peri- implant mucositis: G2 (n=18, 51.4%)) > G1 (n=18, 45.0%)), (p = 0.727)  10.5. Peri-implantitis: None in both  11. Esthetic outcomes:  11.1. Median PES: 7  11.2. Median PESWES: 14  11.3. An acceptable esthetic level in 87.5 % of G1 and 82.9 % of G2 (P = 0.829) |
| Dipti Daga et al (2017) / Prospective study | A) 20  B) 20-50 years (Range)  C) n=9, (45%) : n=11, (55%)  *Inclusion criteria:  -18 – 60 years  -Clinically resorbed ridges (Clinical and radiographic evaluation)  *Exclusion criteria:  -Debilitating disease  -Systemic autoimmune  disease  -Disturbed coagulation ability  -Allergic reaction to metals, medications  -Smokers | Qualitatively and quantita-  tively assessing bone regeneration potential of tentpole technique using beta tricalcium phosphate bone graft in vertically deficient alveolar ridges | - | A) Vertical  B) NS | 1. Regeneration (T0) (Months):  1.1. Placing titanium screws as tenting screws  1.2. Placing a mixture of 2 ml of autologous blood + b TCP synthetic bone graft around the tenting screws to cover up to the screw head  2. Implant placement (T*)  3. Functional rehabilitation (T**)  Screw removal (T6) | A.1.) Titanium screw  A.2.) Diameter: 1.5 mm  A.3.) Various lengths  B) n=31 | 1. Radiography (Intraoral periapical radiograph, ortho-pantomogram, dentascan) (Bone loss) (Pre (T0), postop (T1, T3 and T6))  *Bone height was measured using dentascan  2. Clinical evaluation (Pre (T0), Postop (T1, T3 and T6) and T*):  2.1. Pain (VAS)  2.2. Discharge, loosening of screws and erythema (Present or absent)  2.3. Bone loss  3. OPG (T* and T**)  4. ELISA (Biochemical analysis of Osteopontin and RANKL) (T0 and T3)  5. Histologic analysis of bone core (H&E) (T6) | 1. Complication:  1.1. Pain score:  1.1.1. 24 h (3.65 ± 0.74 (95%CI = 3.30-4.00)) >> T1 (0.20 ± 0.41 (95%CI = 0.20 0.41)), (p = 0.0001)  1.1.2. T3 and T6: Nil pain  1.2. Erythema score:  1.2.1. 24 h: (0.30 ± 0.47 (95%CI = 0.08-0.52))  1.2.2. T1, T3 and T6: Nil erythema  1.3. No postoperative wound infection, erythema or discharge  1.4. Loosening of screw (n=2)  2. Bone height gain:  2.1. T6 (mean 2.87 ± 0.79 (95%CI = 2.50–3.24)) >> T0, (p = 0.0001)  2.2. T0-T6>T0-T3>T0-T1  3. all 31 implants osseointegrated  4. RANKL and Osteopontin level (T0 – T3): No significant change (p > 0.05)  5. H&E (T6): New bone formation  6. Quantitative measurement of new bone (Image J): 80% new vital bone content |
| Joao Batista César Neto (2020) / Cohort | A) 28  *44 sites  B.1.) Overall: 54.14 ± 14.73 years (Mean) (Range 19 – 79)  B.2.) G1: 52.77 ± 13.09  B.3.) G2: 51.95 ± 18.50  C) 35.70%:64.30%  *Inclusion criteria:  -Systemically healthy patients (ASA I and II)  -Older than 18 years  -Under periodontal maintenance (BOP and  plaque index < 20%)  -At least one site per candidate planned for implant-supported rehabilitation exhibiting less than 4mm of bone width at 1 mm below the bone crest or in need of bone reconstruction as diagnosed at the time of digital implant planning  *Exclusion criteria:  -Ongoing periodontal disease  -Pregnant or lactating women  -Smokers (>10 cigarettes/day)  -Existence of a bone metabolic disease  -Currently taking drugs that might influence bone metabolism  -Use of bisphosphonates in the last 4 years  -History of malignancy,  radiotherapy or chemotherapy for malignancy in the past 5 years | Radiographically evaluating the effect of the adjunctive use of tenting screws (TS) for  primary horizontal GBR | G1) Conventional GBR (Control) (n=18 patients) (n=22 sites)  G2) TS + GBR (Test)  *Initial: (n=18 patients) (n=24 sites)  *Final: (n=16 patients) (n=22 sites) | A.1.) Horizontal  A.2.) Number (Sites)  -Total (n=44)  -G1 (n=22)  -G2 (n=22)  A.3.) Jaw (Sites): -G1 (Maxilla (n=12) and mandible (n=10))  -G2 (Maxilla (n=9) and mandible (n=13))  A.4.) Position (Sites):  -G1 (Anterior (n=11) and posterior (n=11))  -G2 (Anterior (n=6) and posterior (n=16))  B) NS  (Coronal portion of the screw head 1 mm apical of the top of the ridge and 4mm away from the buccal bone) | 1. Augmentation (T0) (Months):  1.1. Multiple perforations of the buccal cortical plate  1.2. Placing DBBM mixed with autogenous chips (Harvested from adjacent sites)  G1:  1.3. Covering with a collagen membrane of porcine origin  G2:  1.3. Inserting adjunctive TS only buccally at the center of the horizontal defect (Coronal portion of the screw head 1 mm apical of the top of the ridge and 4mm away from the buccal bone)  1.4. Covering with a resorbable collagen membrane  2. Implant-supported rehabilitation | A.1.) Size:  -Length: 8 or 10 mm  -Diameter: 1.5 mm  -Head diameter: 3.5 mm  A.2) Fixing screw  A.3.) Neodent  B) NS | 1. CBCT (Pre (T0) and postop (T6-8)):  1.1. Linear horizontal measurements  1.2. Alveolar ridge width (RW) and Ridge width change (RWchange): Assessed at 1, 3, 5 and 7 millimeters below the bone crest  2. Weekly follow-ups during first month:  -Frequency, time and magnitude of membrane and tenting screws exposure  *A baseline grid was drawn onto the baseline image with the following reference lines: vertical line (VL) at the bucco-oral center of the ridge (in cases where ridge anatomy hampers the definition of the center, the first third of the ridge will be used as reference); horizontal lines perpendicular to VL at five different levels (at bone crest, 1mm, 3mm, 5mm and 7mm apical of the bone crest)  *The horizontal line drawn at the level of the bone crest and the vertical line (VL) were used to detect horizontal resorption as well as the degree of vertical crestal resorption. Vertical change was considered resorption when bone level after augmentation was  apical to the original bone crest position, and gain was considered when bone level was coronal to the bone crest | 1. Seven patients had surgery at two different time points and contributed to sites for both groups. However, 2 subjects with 2 defects from G2 had to be excluded because matching of the obatined CBCTs was not possible due to artifacts. This resulted in 16 patients and 22 defects in G2.  2. Complications:  2.1. Local swelling in all patients  2.2. Minor membrane exposure (W2 – W3 postop): One site per group (Both sites healed)  2.3. Temporary sensory loss: In 2 cases at lower premolar sites (Both at G2)  2.4. Tenting screw exposure: None  3. Baseline ridge width (RW) in (mm) at 1,3,5 and 7mm apical of the bone crest:  3.1. RW-1: G1 (3.28 ± 1.53) > G2 (3.18 ± 1.57), (Difference: 0.10 ± 0.07), (p = 0.320)  3.2. RW-3: G2 (5.06 ± 2.44) > G1 (5.01 ± 1.63), (Difference: 0.06 ± 0.04), (p = 0.734)  3.3. RW-5: G2 (6.89 ± 3.14) > G1 (6.05 ± 2.15), (Difference: 0.84 ± 0.59), (p = 0.346)  3.4. RW-7: G2 (8.37 ± 3.47) > G1 (7.10 ± 2.54), (Difference: 1.28 ± 0.90), (p = 0.178)  3.5. Overall: G2 (5.87 ± 2.41) > G1 (5.36 ± 1.65), (Difference: 0.51 ± 0.37), (p = 0.555)  4. Follow-up ridge width (RW) in (mm) at 1,3,5 and 7mm apical of the bone crest:  4.1. RW-1: G2 (6.90 ± 2.99) >> G1 (4.54 ± 2.62), (Difference: 2.36 ± 1.67), (p = 0.004)  4.2. RW-3: G2 (9.03 ± 2.52) >> G1 (7.51 ± 2.11), (Difference: 1.52 ± 1.02), (p = 0.022)  4.3. RW-5: G2 (10.65 ± 3.03) >> G1 (9.00 ± 1.91), (Difference: 1.65 ± 1.17), (p = 0.047)  4.4. RW-7: G2 (11.30 ± 3.34) > G1 (9.78 ± 2.26), (Difference: 1.52 ± 1.07), (p = 0.129)  4.5. Overall: G2 (9.47 ± 2.63) >> G1 (7.70 ± 1.85), (Difference: 1.77 ± 1.23), (p = 0.010)  5. Ridge Width change (RWchange) in (mm) measured at 1,3,5 and 7mm apical of the bone crest: 5.1. RWchange-1: G2 (3.72 ± 2.46) >> G1 (1.25 ± 3.05), (Difference: 2.50 ± 1.75), (p = 0.001)  5.2. RWchange-3: G2 (3.98 ± 2.53) >> G1 (2.50 ± 2.02), (Difference: 1.47 ± 0.69), (p = 0.020)  5.3. RWchange-5: G2 (3.75 ± 2.29) > G1 (2.94 ± 1.95), (Difference: 0.81 ± 0.57), (p = 0.416)  5.4. RWchange-7: G2 (2.92 ± 2.15) > G1 (2.68 ± 2.43), (Difference: 0.24 ± 0.17), (p = 0.986)  5.5. Overall: G2 (3.57 ± 2.38) >> G1 (2.35 ± 1.64), (Difference: 1.22 ± 0.86), (p = 0.009)  6. Follow-up lingual Ridge Width Change (LRWchange) in (mm) at 1,3,5 and 7mm apical of the bone crest (n=19 sites (G2 (n=10) and G1 (n=9) in 10 patients)): 6.1. LRWchange-1: 0.54 ± 1.0  6.2. LRWchange-3: 1.15 ± 0.81  6.3. LRWchange-5: 1.03 ± 0.72  6.4. LRWchange-7: 1.03 ± 1.47  6.5. Overall: 0.94 ± 1.00  7. Horizontal crestal resorption was observed at the first millimeter (Mean):  7.1. G1 (n=12 sites): -1.20 ± 3.80 mm  7.2. G2 (n=2 sites): -1.66 ± 4.30 mm  7.3. No correlation between horizontal crestal resorption and horizontal ridge width at baseline (r = 0.04, p>0.05)  8. Vertical crestal resorption: 8.1. G1 (n=8 sites): -1.11 ± 0.70 mm  8.2. G2 (n=2 sites): -0.90 ± 0.04 mm  8.3. An intra-class correlation coefficient of 0.99 was obtained regarding baseline 1st mm width to vertical crestal resorption  9. Multilevel linear regression analysis:  9.1. Comparing all variables for RWchange-1: Age, gender and arch (i.e. upper and lower) did not influence data variance  9.2. Adjusted for anterior/posterior sites: G2 (1.86 mm more bone gain on average) >> G1, (p <0.001)  9.3. Posterior sites had undergone greater bone gain when compared to the anterior sites (p <0.001), even after the adjustment for groups |
| Bach Le et al (2016) / Retrospective clinical case series | A) 14  B) 36.78 ±13.9 (Mean)  (Range 20-64)  C) 7:7  *Inclusion criteria:  -Treatment of facial bone loss and gingival  recession around at least 1 implant-supported, single-tooth restoration in the maxillary esthetic zone  -Periodontally and  systemically healthy nonsmokers  -At least 18 years of age with a soft tissue dehiscence at the buccal aspect more than 2 years after loading  -Implants had to be free of  periimplantitis  -No interproximal attachment loss for the teeth neighboring the implant  -An unrestored and normally positioned contralateral tooth without a recession defect  -Have completed a 1-year postoperative clinical evaluation  *Exclusion criteria:  -Patients with implants placed at severe labial inclinations or too deep relative to the crestal bone | Use of GBR and coronal advancement flap with resorbable membrane and allograft | - | A.1.) Maxilla  A.2.) Implant location:  -Central (n=6)  -Lateral (n=8)  A.3.) Horizontal  B) NS | 1. GBR + implant placement (T0) (Months):  1.1. Hydrating allograft material and mixed with the patient’s blood  1.2. Packing particulate mineralized bone allograft material (Puros Cancellous Bone Allograft) (Over contoured by approximately 20-30%)  1.3. Covering with a resorbable xenogenic membrane (CopiOs Pericardium)  1.4. Connecting a wide healing abutment to the implant  1.5. Placing a roughened titanium tenting screw 3-4 mm below the implant platform  2. Interim prosthesis (T0)  3. Screw-retained provisional prosthesis (T4): For 4-5 months  4. Definitively restored with either a screw-retained or cement-retained single-tooth restoration | A) Titanium screw  B) Diameter: Range 3.3 - 4.7 mm | 1. Clinical examination (Oral hygiene, soft tissue health, the position and emergence profile of the implant relative to the labial plate and adjacent  teeth, gingival contour, percentage of gingiva visibility when the patient smiled, and the shapes of the prosthetic and clinical crowns) (Pre (T0) and postop (T3))  *Soft tissue thickness: 1.5 mm below gingival margin  *Reference: Unrestored contralateral tooth normally positioned without recession defect  2. PA and CBCT (Pre (T0) and postop (T3)):  2.1. Implant labial bone thickness at the crestal (2 mm from crest) and mid-implant levels  2.2. Dimensions of the peri-implant crestal bone defect  *Implant buccolingual angulation was recorded: Cingulum, incisal, or labial based on the screw access hole position on provisional restorations  3. Intraoral photographs (Pre (T0) and postop (T0+))  *Repeating clinical measurements (T3) | 1. Complications:  1.1. All patients successfully completed periodontal surgery  1.2. One patient presented with 3 implants with mid-facial recession (One implant was removed in this patient so that only 2 implants were treated)  1.3. All other patients presented with a single implant each  1.4. No complications or adverse events during surgery or postoperative healing  2. All postoperative tissue changes from their preoperativenstate were statistically significant (p < 0.05)  3. Cumulative post-operative hard tissue changes (mm) (n=14) (T12): 3.1. Labial Bone Thickness (Midsection of implant):  -Mean: 2.07 ± 0.81, (p = 0.0001)  -95% CI of mean changes: 1.60-2.53  3.2. Labial Bone Thickness (Crest of Ridge) (2 mm from crest):  -Mean: 1.84 ± 0.89, (p = 0.0002)  -95% CI of mean changes: 1.32-2.35  3.3. Soft Tissue Thickness:  -Mean: 1.28 $\pm$ 0.53, (p = 0.0001)  -95% CI of mean changes: 0.97-1.58  3.4. Keratinized Tissue Width:  -Mean: 1.29 $\pm$ 0.81, (p = 0.0001)  -95% CI of mean changes: 0.82-1.75  3.5. Gingival Margin Height:  -Mean: 1.23 $\pm$ 0.53, (p = 0.0001)  -95% CI of mean changes: 0.92-1.53  4. Preoperative soft (table 2) and hard tissue measurements (mm):  4.1. Labial Bone Thickness (Midsection of implant) (Mean): 0.64 mm  4.2. Labial Bone Thickness (Crest of Ridge) (Mean): 0.17 mm |
| Gerardo chacon et al (2024) / Retrospective case series | A) 10  B) 35.1 years (Mean) (Range 15 – 55)  C) 5:5  *Inclusion criteria:  -Vertical regeneration of combined intraosseous and extraosseous defects ( $\leq$ 5 mm) for subsequent placement of implants in adequate prosthetic positions  -Healthy  -Requiring VRA  *Exclusion criteria:  -Smoker  -Excessive alcohol consumer  -Carriers of uncontrolled systemic diseases | Presenting a surgical approach for vertical anterior augmentation using tent-pole screws and collagen membranes for predictable outcomes | - | A.1.) Vertical  A.2.) Anterior  A.3.) Jaw:  -Maxilla (n=8)  -Mandible (n=2)  A.4.) Number of replaced teeth: 1 – 5 per patient  B.1.) Vertical defect depth: 7 mm (Mean) (Range 5-12 mm) | -Implants were left submerged to heal for 3-6 months before uncovering  -Placing definitive restorations within few weeks after implant uncovering  -If the VRA was not able to provide complete defect resolution, implants were placed supracrestal to the preplanned position and a second grafting procedure was performed simultaneously  1. Regeneration (T0) (Months):  1.1. Combining autogenous bone (Harvested from ramus) with DBBM (50:50)  1.2. Medullary perforations  1.3. Placing 1 – 4 screws (According to the extension of the defect) 1 mm deep in relation to the bone crest of the neighboring tooth or the adjacent edentulous ridge  1.4. The bone graft was contained using the membrane as the palatal wall  1.5. Covering with a second layer of the same collagen membrane (Cross linked collagen membrane)  1.6. External fixation of membrane with sutures  *Avoiding provisional any structure for a minimum of 90 days  2. Reentry and implant placement (T9.3 (Mean), (Range 6 - 12.6 months))  3. Fixed provisional restoration (Essix appliance): Seperated from the surgical area by at least 3 – 5 mm | A.1.) Length: 1.2 mm  A.2.) Brand: LeForte system, Jeil medical  A.3.) n= 1-4 screws per defect  B.1.) Torque: 33 $\pm$ 4.21 Ncm (Mean) (Range 25 – 35)  B.2.) Brand (Cases):  -MIS V3 implants (n=9)  -Tapered Plus implant (BioHorizons) (n=1) | 1. Measurement of defect (24 h, T12)  2. CBCT (T9)  3. Flap exposure score: Yes (1) and no (0)  4. Follow-up (24 h, 1and 2 W, T1, T3, T6, T9 and T12)  5. Radiography (T6)  6. Panoramic (T0 and T12)  7. Healing time for reentry and implant placement: 9.3 months (Mean) (Range 6-12.6) | 1. Total bone gain at reentry (T9): 6.2 mm (Mean) (Range 5-9)  2. Complication:  2.1. D15: Flap exposure ( < 5 mm) (n=1)  2.2. All cases healed uneventfully except 1 (Flap exposure < 5 mm) which finally had tissue closure around the sixth week  3. Regeneration:  3.1. Increase in bone gain: 5 - 9 mm of defect resolution in all cases  3.2 Mean defect fill (T12): 6.2 $\pm$ 1.61 mm  3.2. Flap-exposure case had a minimum bone gain of 6 mm out of the original 12-mm vertical defect (50% defect fill)  3.4. Defect contraction index (T12): 0.8 $\pm$ 1.87 mm  4. CBCT (T9): Bone growth  5. Histologic section (T9): New bone formation, few deposits of type I collagen in the fibrous tissue surrounding the trabeculated bone, and fragments of trabecular and lamellar bone |
| Robert E. Marx et al (2002) / Case series | A) 64  B) 65 years (Mean) (Range 44-81 years)  C) 3:61 (95%)  *All women were postmenopausal  *Inclusion criteria:  -At least 3 years of follow-up (mean: 4.9 years)  -Resorbed mandible of 6-mm height or less at its greatest height anterior to the rami  *Exclusion criteria: -History of oral cancer surgery, radiotherapy, or chemotherapy  -Active infections or continuity defects | Long-term data on a novel surgery based on the hypothesis of soft tissue volume maintenance via tenting with dental implants | - | A.1.) Mandible  A.2.) Vertical  B.1.) $\leq$ 6 mm  *Greatest height anterior to the rami  The remaining 9 to 11 mm of their length will project above the native bone level | 1. Implant placement + Bone grafting (T0) (Months):  1.1. Placing implants as tent poles in the buccolingual center of the resorbed mandible  *The remaining 9 to 11 mm of their length will project above the native bone level.  *Placing implants 1 cm apart and parallel to each other.  *The first is placed with its center 5 mm anterior to the emergence of the mental nerve.  1.2. Placing and then compacting the bone graft (Harvested from the posterior ilium) posterior to the mental nerve  1.3. Placing cancellous marrow material from the posterior on each side forward toward the midline (Completely compacting and covering top of implants at the implant region)  *PRP was used in all of the bone grafts in this study  2. Temporary or provisional prosthesis (W2)  3. Uncovery (T3-4):  -Bone profiler to correctly seat the abutments | A) No screws  B.1.) Dental implants as tent poles  B.2. Standard dental implant  B.3.) Number (Implants):  -n=5.6 (Per patient)  -n=356 (Total)  B.4.) Size:  4 mm  -Length:15 mm  B.5.) Form:  -Press-fit (n=128 implants, n=23 patients)  -Threaded (n=228 implants, n=41 patients) | 1. OPG (Bone height) (Pre (T0) and follow-up)  2. Follow-up (3.0 – 10.1 years (Mean: 4.9 years)) | 1. Complications:  1.1. Osseointegration failure: 2 implants in 2 different subjects (Press-fit (n=1, 0.77%), Threaded (n=1, 0.44%)) (Total nonintegration incidence: 0.5%)  1.2. Not using implants by prosthodontists: n=18 of 356 implnats (5.1%)  *In each case the prosthodontist thought the implant was positioned too far buccally  *No case where more than 1 implant remained unused by the prosthodontist, and it never prevented the fabrication of a functional prosthesis.  1.3. No fracture either during surgery or in the subsequent follow-up time.  1.4. No bone resorption below the height of the implants.  *The bone in the regions posterior to the mental foramen where no implant was placed also retained its bone height with no evidence of resorption.  1.5. Short-term (3 weeks) mental nerve paresthesia:  -Unilateral: n=8 cases (12.5%)  -Bilateral: n=3 cases (4.6%)  1.6. No long-term or permanent paresthesia  1.7. Early exposure and loss of an implant: n=1 case (1.6%)  *In this case, the bone graft proceeded to consolidate without a complication and the patient progressed to a fully functional permanent prosthesis.  2. Osseointegration at the time of follow-up:  -Press fit: n=127, 99.2%  -Threaded: n=227, 99.6%  -Total: n=354, 99.5%  3. Vertical mandible height:  3.1. Before surgery (T0): 4.8 mm (Mean) (Range 2 - 6 mm)  3.2. Post surgery follow-up (3-10 years): 15 mm (Mean and range) (The height of the implants)  3.3. Bone height gain: 10.2 mm (Mean) (Range 9 – 13 mm)  3.4. The bone grafts in all cases were observed to maintain their bone height and become more radiographically dense over time.  3.5. Bone grafts that were originally placed above the level of the implants were consistently observed to remodel down to the top of the implants but no further.  4. In all 64 cases a functional overdenture was made and was functional at the time of the most recent follow-up  5. Delaying prosthodontic rehabilitation for more than 18 months (Due to economic reasons): n=11 cases  *In each of these cases the bone height was maintained despite the lack of functional stimulation.  *The radiographic density increased once loading occurred.  6. Secondary surgical procedure in the form of a split-thickness skin graft vestibuloplasty for optimal prosthetic function: n=11 cases (17.2%)  * All of these 11 cases were ones in which a  previous subperiosteal implant or transmandibular  implant with long-term chronic infection (n=6) had  been present and had been removed or HA ceramic  particles (n=5) had been placed and were debrided  before the grafting procedure. All 11 had a more  severe soft tissue loss and scarring from these previ-  ous reconstructive attempts |
