# Supplementary material for: Tenting screw (tent pole) technique in vertical and horizontal alveolar ridge augmentations: a systematic review and meta-analysis
Source: Maxillofac Plast Reconstr Surg. 2026 Aug 14;48(1):22. doi: 10.1186/s40902-026-00522-y (PMC13476430; doi:10.1186/s40902-026-00522-y)
Supplement: Supplementary file 2 [file 40902_2026_522_MOESM2_ESM.docx]

**Supplementary Table 2.** GRADE Certainty Assessment.

**Question:** Tenting Technique compared to Conventional GBR for Horizontal Ridge Augmentation

| **Certainty assessment** | | | | | | | **№ of patients** | | **Effect** | | **Certainty** | **Importance** |
| --- | --- | --- | --- | --- | --- | --- | --- | --- | --- | --- | --- | --- |
| **№ of studies** | **Study design** | **Risk of bias** | **Inconsistency** | **Indirectness** | **Imprecision** | **Other considerations** | **Tenting Technique** | **Conventional GBR** | **Relative (95% CI)** | **Absolute (95% CI)** |  |  |
| **Bone Gain (follow-up: range 9 months to 12 months)** | | | | | | | | | | | | |
| 5 | non-randomised studies | not serious | not serious | not serious | serious^a^ | none | 83 | 90 | - | MD **0.49 mm higher** (0 to 0.97 higher) | ⨁⨁⨁◯ Moderate^a^ |  |

**CI:** confidence interval; **MD:** mean difference

#### Explanations

a. The imprecision is serious due to a predominantly non-randomized body with an effect of 0.49 mm (95% CI = 0.00–0.97, crossing the null) and P = 0.05.
